# Simultaneous Lignin Fractionation and Functionalization under Mild Conditions via Reactive Extraction

**DOI:** 10.1021/acsomega.6c01031

**Published:** 2026-07-17

**Authors:** Davide Rigo, Nadine Kohlhuber, Lukas Fliri, Irina Sulaeva, Daryna Diment, Oliver Musl, Antje Potthast

**Affiliations:** † Department of Bioproducts and Biosystems, 174277 Aalto University, Vuorimiehentie 1, 02150 Espoo, Finland; ‡ Department of Natural Sciences and Sustainable Resources, Institute of Chemistry of Renewable Resources, 232069 BOKU University, Konrad-Lorenz-Strasse 24, 3430 Tulln an der Donau, Austria; § Core Facility Analysis of Lignocellulosics (Alice), 27270 BOKU University, Konrad-Lorenz-Strasse 24, 3430 Tulln an der Donau, Austria

## Abstract

Reactive extraction (REx) is a promising route to simultaneously fractionate and upgrade (functionalize) lignin under mild conditions. In this work, hydrothermally treated wood solids (*T* = 180 °C, *t* = 45 min, liquid-to-solid ratio = 1) were refluxed for 1 h using acetic acid, formic acid, and ethanol as solvent-reagents. In addition, Amberlyst 15 (Amb-15) was tested as a heterogeneous acid catalyst for REx. The design of the reaction setup allowed to minimize product losses, even when Amb-15 was used, which could be easily removed from the reaction mixture. The extracted lignins showed appreciable amounts of native linkages (28.8–46.4/100 Ar), molar masses of 2.8–13.7 kDa (*M*_w_), and total hydroxy group contents of 4.6–5.8 mmol/g. HSQC NMR analysis proved the incorporation of ethoxy, acetyl, and formyl groups in the extracted lignins in 1.2–2.0/100 Ar, 39.2–39.4/100 Ar, and 12.6–15.4/100 Ar, respectively. The molar masses of the residual cellulosic solids ranged from DP_w_ of 2900 to 1000, and ^13^C NMR solid-state and solution-state NMR analyses confirmed the functionalization of the residual cellulosic solids as well, though to a low extent.

## Graphical abstract

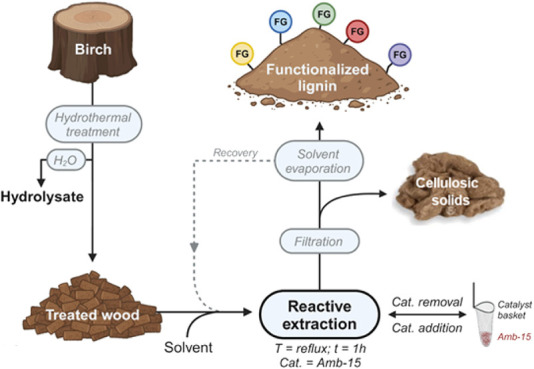

## 1 Introduction

Lignocellulosic biomass is the most promising sustainable feedstock to produce aromatic chemicals, fuels, and materials.^1,2^ Biomass—which is mainly composed of cellulose, hemicellulose, and lignin—is often subjected to chemical fractionation (i.e., pulping), enabling the selective use of its components for different applications. Traditionally, the pulp and paper industry makes use of pulping processes to extract lignin (and hemicelluloses) from woody biomass to produce a variety of (pure) cellulosic materials.^3–5^ While the cellulosic fraction finds its use in material applications, the extracted lignin is incinerated for energy recovery to provide heat and energy for the pulping operation. However, the utilization of technical and biorefinery lignins has become (again)^6,7^ a timely field of investigation, also for high-value applications.^1^ For instance, great efforts have been made to make use of lignins in different applications, e.g., materials,^8,9^ fuels,^10^ energy storage,^11^ and so on.^12,13^ However, most of the currently isolated lignins—especially Kraft lignins—still face serious challenges due to their very condensed and heterogeneous structure even when used in low-value applications.^14,15^

One way to improve the quality—for instance, a high β-O-4 content or a high degree of functionalization—of the extracted lignin is to adapt the extraction process to prevent the excessive changes in lignin structure common to conventional methods, while preserving good quality pulp. For instance, the plethora of new pulping processes aiming to extract a less altered lignin is subsumed under the umbrella term “lignin-first biorefinery”.^16^ Lignin-first processing utilizes stabilization strategies during fractionation (e.g., reductive catalytic fractionation (RCF), aldehyde assisted, diol assisted, oxidations, etc.)^16–19^ to minimize condensation (C–C bond formation) during lignin extraction.^16,20–23^ However, optimal extraction of one component often requires sacrificing the yield or quality of another, rendering holistic process design rather cumbersome. The ability to tune the structural characteristics of all the extraction products (lignin, cellulose, and hemicellulose simultaneously) is thus another important feature new processes should implement to demonstrate their competitiveness compared to conventional pulping.^20–27^ The “quality” of the isolated lignins and cellulosic products can also be improved via chemical functionalization. Common lignin modifications include alkylation, etherification (e.g., alkoxylation), esterification (e.g., acetylation, formylation), amination, and phenolation to introduce new functionalities or mask existing ones.^28–30^ Similarly, cellulose functionalization has been widely investigated.^31,32^ Modification often improves the performance of lignin and cellulose in several applications.^1,32–38^

Despite the obvious benefits, conventional modification protocols require isolated lignin or cellulose and often involve highly reactive, corrosive, and/or toxic chemicals, which pose health and safety hazards in addition to sustainability issues. In addition, for instance, total conversion of lignin functional groups or a very high degree of functionalization is generally targeted, even though this may not always be required to maximize performance.^1,39,40^ The reactive extraction approach allows partial modification of lignin already during its extraction from biomass while using only commonly used solvents and reagents. In this way, different degrees of substitution can be achieved very efficiently, which are suitable for improving the lignin properties as needed.^1,39,40^ Moreover, reactive extraction (REx) differs from organosolv pulping as the latter requires longer times and higher temperatures and pressures. For example, formic acid has been used in organosolv processes to extract lignin from biomass;^41–44^ however, the severe pulping conditions used have often led to the isolation of a fairly degraded and condensed lignin instead of a modified one.^45^

REx has been mainly carried out under homogeneous catalysis conditions using HCl,^35^ supercritical ammonia–water mixtures,^46^ sulfuric acid,^39,47^ and metal salts.^48^ Although homogeneous catalysis is powerful, it poses drawbacks such as complicated catalyst separation and recycling, e.g., neutralization of acids and bases prior to the isolation of the products.^49^ Alternatively, functionalized lignin can be extracted via precipitation or the use of ion exchange resins. However, traces of the catalyst remained in the isolated products. On the other hand, in general, heterogeneous catalysis facilitates the separation of the catalyst from the reaction mixture and offers the opportunity to be reused after washing/reactivation.^50,51^ Despite such advantages, heterogeneous catalysis has been mainly applied for the depolymerization of lignins (i.e., reductive catalytic fractionation) so far,^52^ with little attention given to their functionalization.

Our group recently introduced the AquaSolv Omni (AqSO) process as an example of a new biorefinery generation that aims for a holistic valorization of all biomass fractions.^53,54^ The AqSO biorefinery concept comprises the hydrothermal treatment of birch wood at a low liquid-to-solid ratio and different severities, followed by acetone extraction of the treated solids. We have also recently implemented REx^39^ as an upgrade within the AqSO biorefinery concept (Figure 1A), replacing acetone with ethanol and H_2_SO_4_ as the solvent/reagent and catalyst, respectively.^39,53,54^ This allowed for further tuning of the characteristics of the extracted lignin and residual cellulosic solids by chemical modification.

**Figure 1:**
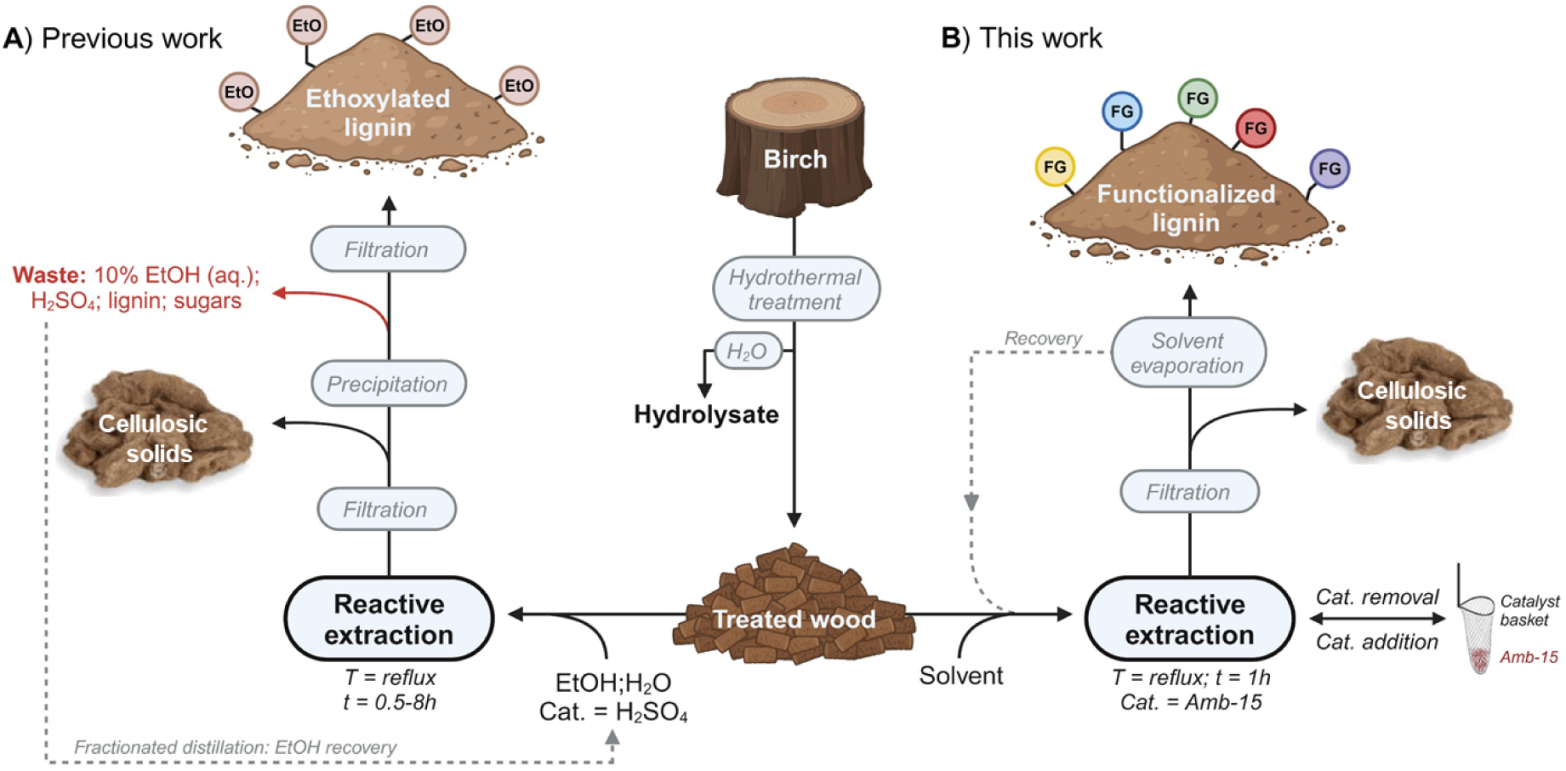
REx approaches. (A) Our reported work^39^ on the ethanol-mediated REx under homogeneous catalysis (H_2_SO_4_). (B) Present work on the solvent-mediated (EtOH, AcOH, or HCOOH) REx under catalyst-free conditions or heterogeneous catalysis (Amb-15). The new approach facilitates catalyst recovery and allows to fully isolate the product with no waste produced.

In this context, we expanded the scope of REx by the use of acetic acid (AcOH) and formic acid (HCOOH) to include lignin acetylation and formylation in addition to ethoxylation with ethanol (EtOH). We also tested Amberlyst 15 (Amb-15)—a strongly acidic cationic ion-exchange resin—as a heterogeneous acid catalyst for REx to replace H_2_SO_4_ or other potential homogeneous acid catalysts (Figure 1B). Overall, the purpose of this work is to show a representative mild REx condition using different solvents to afford different functionalizations of lignin and cellulose.

## 2 Results and Discussion

### 2.1 Reaction Setup and Product Yields

Initially, AqSO biorefinery^53,54^ was applied to woody biomass. A hydrothermal treatment of birch wood sawdust was performed as described earlier^39,53,54^ at a severity factor (P-factor; see Section 4) of 500 (*T* = 180 °C; *t* ∼ 40 min) and a liquid-to-solid ratio of 1. A simple water extraction allowed to partially separate the hemicellulose fraction from the residual treated solids (more details are given in our previously reported works), which thus mainly contained lignin and insoluble cellulose fibers, and facilitated the subsequent lignin REx. The compositional analysis of the hydrothermally treated wood solids is reported in Table S1.

REx was then carried out. In our recently reported work^29^ the use of H_2_SO_4_ as a catalyst posed some drawbacks. First, H_2_SO_4_ is a corrosive chemical which imposes higher standards for reactor materials and occupational safety. Second, H_2_SO_4_ is a homogeneous catalyst, and thus its separation is considerably complicated. For instance, isolation of the ethoxylated lignins after REx was incomplete as a certain lignin fraction remained in solution after precipitation by solvent shifting,^39^ reducing the lignin yield by 5–10 wt %. This is a common issue in homogeneous catalysis. Thus, in this work we applied either catalyst-free conditions or Amb-15 as the heterogeneous acid catalyst (Table 1). For catalyzed reactions, facile separation of Amb-15 was achieved by removing the catalyst basket from the mixture (Figure 1B). Amb-15 was chosen as the catalyst since it has been widely proven effective for several acid-catalyzed reactions, such as esterification,^55,56^ acetalization,^57,58^ Michael addition,^59^ among others.^60^ Amb-15 was also chosen since it does not show size selectivity as other catalysts such as zeolites do.

**Table 1:** Summary Table of the Reaction Conditions and Samples Labeling of the Extracted Lignin (EL) and the Cellulosic Solid (CS) Samples^a^

	conditions			
entry	solvent	catalyst	EL	CS
1	EtOH	—	EL-1	CS-1
2		Amb-15	EL-2	CS-2
3	AcOH	—	EL-3	CS-3
4		Amb-15	EL-4	CS-4
5	HCOOH	—	EL-5	CS-5
6		Amb-15	EL-6	CS-6

a Reaction conditions: *t* = 1 h, *T* = reflux (78 °C, 118 °C, and 101 °C for EtOH, AcOH, and HCOOH, respectively).

Two fractions were obtained after REx: extracted lignin (EL) and cellulosic solid (CS). The hemicellulose fraction is not present within the products of REx since it has been almost fully removed by water extraction after the initial hydrothermal treatment (*vide supra*). The lignin fraction (EL) comprises all of the products that are soluble in the organic solvent, while the insoluble fraction refers to CS. Since some lignin samples (ELs) contained residual carbohydrates, as confirmed by methanolysis,^61,62^ carbohydrate-corrected lignin yields were also calculated (Table S4).

The yield of all products was mainly dependent on the used solvent, while only a minor effect of the catalyst was observed (Figure 2B and C). EtOH (p*K*_a_ = 15.9)^63^ afforded lower EL yields (14–15 wt %) and a lower degree of delignification (51–62 wt % of residual lignin, RL) compared to the other used solvents. Using the more acidic AcOH (p*K*_a_ = 4.75)^64^ showed some increase in EL yields (18–22 wt %) and a higher degree of delignification (43–44 wt % of RL). Further increasing the acidity of the solvent by using HCOOH (p*K*_a_ = 3.75)^65^ showed a significant increase in EL yields (42–43 wt %) and degree of delignification (6–10 wt % of RL). Remarkably, HCOOH allowed for almost full delignification of pretreated birch wood under mild conditions (*T* = 101 °C, reflux temperature of HCOOH; *t* = 1 h; Figure 2C and Tables S1 and S2). However, the purity of the EL fraction was impaired by certain amounts of lignin–carbohydrate complexes (LCCs) and sugars, as confirmed by HSQC NMR and methanolysis,^62^ which explains the ca. 160% yield of the EL fraction (corrected yield considering carbohydrate content for EL-6 = 109 wt %: Table S4) with respect to the initial lignin content (Figures 2C and 3, and Table 2). Moreover, the total recovery of products (ca. 105 wt %) exceeded the original sample mass which could be related to a high degree of functionalization, i.e., formation of esters of formic acid in lignin and polysaccharides (*vide infra*). The mass balance (EL + CS) was quantitative in all cases, improving an issue that was encountered in our previous work.^29^ Notably, the solvent-reagents could be distilled and recovered.

**Figure 2:**
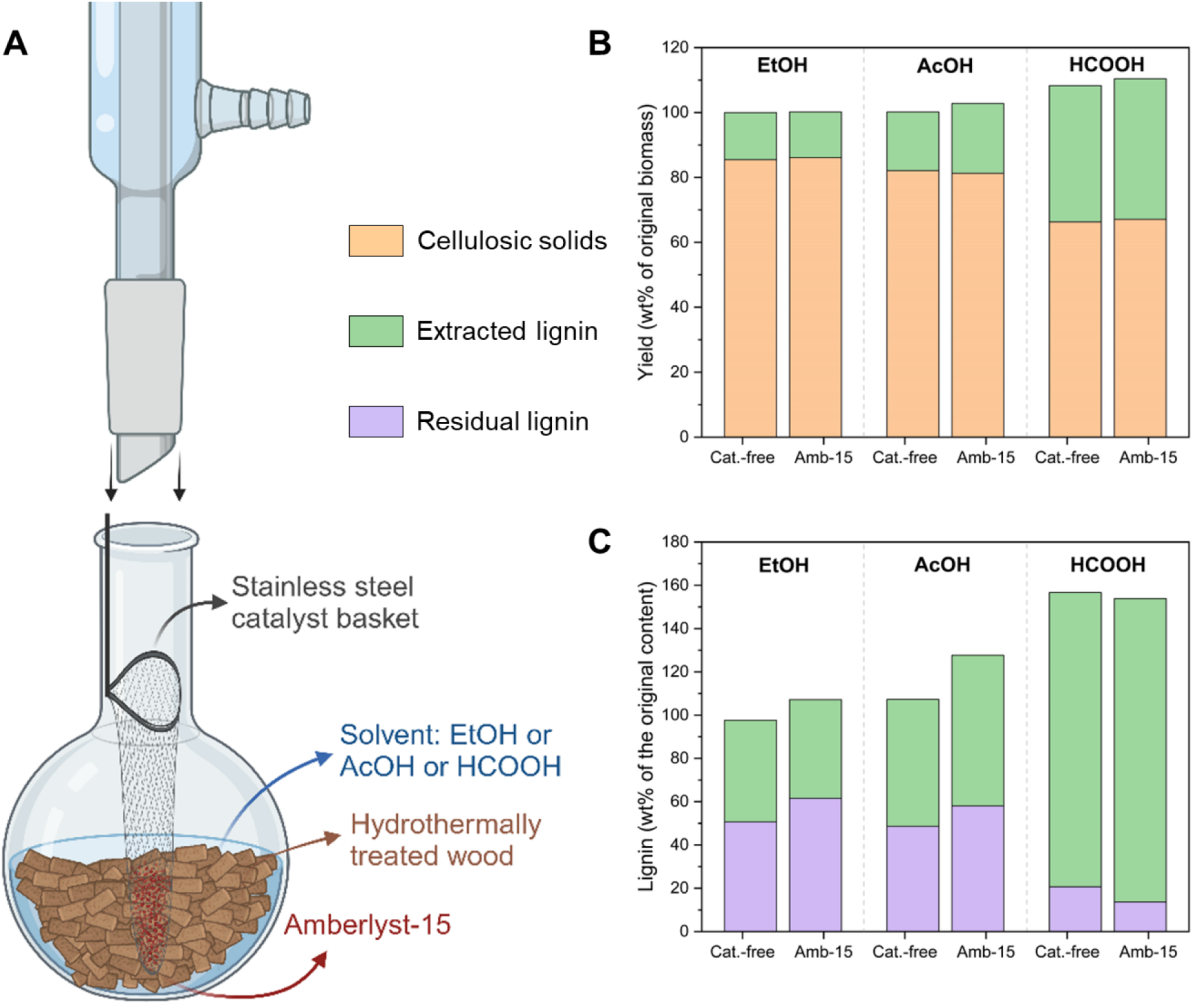
(A) The apparatus used to carry out the Amb-15 reactive extraction of lignin. A stainless steel catalyst basket was filled with Amb-15 and placed inside a round-bottom flask with the hydrothermally treated wood solids (S-500) and the solvent of choice. At the end of the reaction, the catalyst basket could be easily removed. The catalyst basket was not used in the case of catalyst-free experiments. (B) Yields of CS and EL with respect to the weight % of the original dry biomass (S-500). (C) Yields of EL and residual lignin in CS with respect to the lignin content in the original biomass (S-500). The hemicellulose fraction has been almost fully water-extracted after hydrothermal treatment and thus is not present within the products of REx. Data are reported also in Table S2. Compositional analysis of CS is reported in Table S1. Corrected lignin yields considering carbohydrate content are reported in Table S4.

**Table 2:** Quantification of Lignin Units via HSQC NMR Analysis and Their Integration Ranges^a^,^e^

		integration range (ppm)		lignin sample					
entry	lignin unit	δ_H_	δ_C_	EL-1	EL-2	EL-3	EL-4	EL-5	EL-6
1	S/G ratio	S_2,6_: 5.6–7.7 G_2_: 6.1–7.6	S_2,6_: 99.3–109.0 G_2_: 109.2–113.6	2.6	2.7	2.9	2.8	2.5	2.3
2	Conj S_2,6_	5.8–7.6	104.1–107.8	6.0	7.4	7.8	7.4	7.6	7.5
3	β–O–4/α-OH (α)	4.5–5.0	69.2–73.8	23.0	23.4	23.9	21.7	15.0	11.7
4	β–O–4/α-CO (β)	5.0–5.3	81.5–84.1	0.5	1.4	0.4	0.4	0.5	0.4
5	β–O–4/α-Ac (α)	5.4–6.0	71.1–80.6	n.a.	n.a.	9.2	14.9	n.a.	n.a.
6	β–O–4/γ-Ac (γ)	4.1–4.5	59.7–66.5	n.a.	n.a.	6.2	4.9	n.a.	n.a.
7	β–O–4/γ-formate (γ)	4.1–4.5	59.7–66.5	n.a.	n.a.	n.a.	n.a.	15.4	12.6
8	Total β–O–4^b^			24.7	26.8	33.5	37	30.9	24.7
9	β–5 (phenyl coumaran) (α)	5.2–5.6	85.1–88.9	1.9	2.1	2.4	1.9	2.2	1.8
10	β–β (resinol) (α)	4.5–4.8	83.3–86.8	7.0	7.3	7.6	7.5	3.1	2.3
11	Methoxy	3.2–4.9	53.0–57.4	123.4	133.3	136.7	135.7	147	143.4
12	Acetyl	1.6–2.0	17.9–22.1	2.7	3.1	39.4	39.2	21.1	22.0
13	Vinyl–alkyl	5.7–6.4	123.7–133.6	9.7	8.6	5.4	5.8	3.9	3.7
14	Vinyl–aryl	6.6–7.2/7.4–7.7	123.8–130.3/126.8–134.5	9.8	12.4	5.8	5.1	5.0	4.7
15	Ar–CHO	9.5–10.0	189.2–192.5	3.7	4.7	5.0	4.4	3.5	2.6
16	Fur–CHO	9.4–9.7	176.1–179.6	1.7	1.9	2.3	1.0	2.9	0.7
17	Fur–Ox	7.4–7.7	149.6–156.7	1.3	2.0	1.4	1.2	0.3	0.0
18	Benzyl ethers	4.5–4.9	79.6–83.3	4.9^b^	5.3^c^	5.9	2.2	n.a^.c^	n.a.^d^
19	Glucuronic ester	4.6–4.7	99.4–101.4	0.3	0.3	0.3	0.4	n.a.^d^	n.a.^d^
20	Internal carbs			3.9	4.5	4.6	4.2	31.2	27.2
21	Terminal carbs			1.4	1.6	1.5	1.7	108.4	100.4
22	Total carbs^d^			5.3	6.1	6.1	5.8	139.6	127.6
23	CH_3_–CH_2_–OR	0.9–1.2	12.2–15.9	1.2	2.0	n.a.	n.a.	n.a.	n.a.
24	HCOOR	7.7–8.6	158.3–164.5	n.a.	n.a.	n.a.	n.a.	11.0	9.8

a The lignin units are expressed per 100 Ar.

b For EL-1 and EL-2: β–O–4/α-OH + β–O–4/α-CO + β–O–4/α-OEt; for EL-3 and EL-4: β–O–4/α-OH + β–O–4/α-CO + β–O–4/α-OAc; for EL-3 and EL-4: β–O–4/α-OH + β–O–4/α-CO + β–O–4/γ-formyl.

c In the case of EL-1 and EL-2, benzyl ethers include not only LCC linkages but also ethoxy-ethers.

d Signal overlap did not allow for quantitation.

e Sum of internal and terminal polysaccharides. The reaction conditions for lignin sample preparation are *t* = 1 h; *T* = reflux. EL-1: EtOH; EL-2: EtOH/Amb-15; EL-3: AcOH; EL-4: AcOH/Amb-15; EL-5: HCOOH; EL-6: HCOOH/Amb-15. n.a. = not applicable.

**Figure 3:**
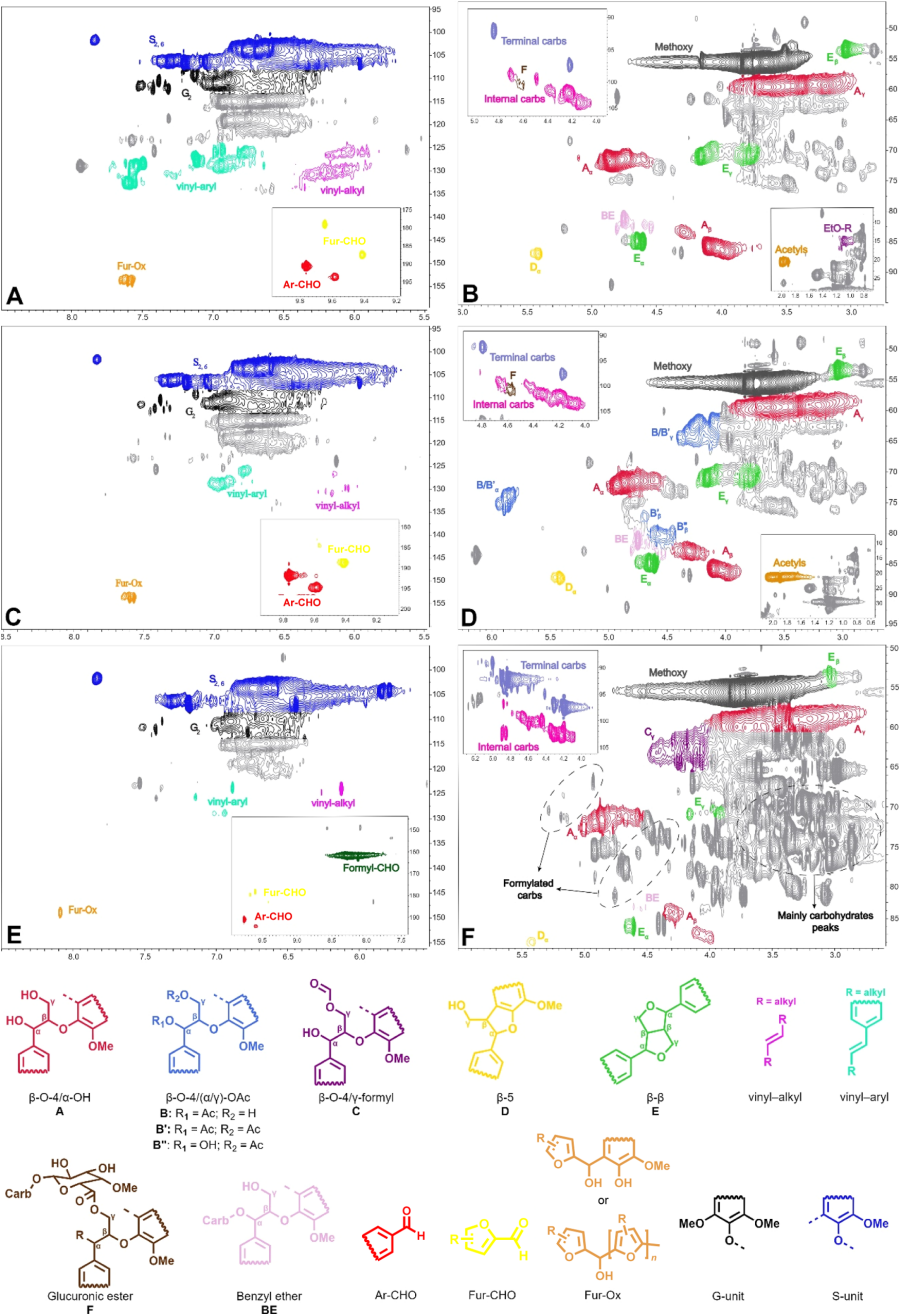
HSQC spectra of ELs. The aliphatic region of EL-2 (A), EL-4 (C), and EL-6 (E) and the aromatic region of EL-2 (B), EL-4 (D), and EL-6 (F) are reported. The presence of Fur-Ox and **F** structures (in the bottom) was tentatively assigned based on earlier studies.^53,54^

### 2.2 Chemical Structure of Extracted Lignins

^1^H–^13^C heteronuclear single quantum coherence spectroscopy (HSQC) NMR analysis elucidated the main structural units present in EL after the REx. Two-dimensional HSQC NMR provided detailed structural information on lignin with good spectral resolution and enabled the quantification and relative comparison of specific functional motifs in structurally similar lignin samples, though it did not provide quantitative data at an absolute level. Interestingly, in some cases, our group found good agreement between HSQC and quantitative ^13^C NMR analyses, for example, in the quantification of β-O-4 linkages.^39,53^ The results of the semiquantitation of lignin structural motifs are reported in Table 2.

HSQC NMR spectra of samples obtained under catalyzed conditions using EtOH, AcOH, and HCOOH, respectively (EL-2, EL-4, and EL-6, respectively), are reported in Figure 3. The spectra of the noncatalyzed samples can be found in the Supporting Information (SI) and show strong structural similarities with the catalyzed samples (Figures S1, S3, and S5).

The syringyl-to-guaiacyl ratio (S/G) of the EL ranges from 2.3 to 2.9 which is close to the native ratio of about 3.^66^ The S/G ratio was more affected by the choice of solvent (±4–21%) than by the presence of Amb-15 (±3.5–8.5%). For instance, using HCOOH led to a ca. 15% lower S/G ratio compared to EtOH and AcOH, which was consistent with a preferred extraction of S-unit lignins at lower severity/acidity, as we recently observed.^53,54^ The prevalence of native C–O bonds in the EL (i.e., β–O–4, β–5, and β–β) is another good indicator of the low process severity. Under the acidic conditions of REx, lignin is primarily degraded by hydrolytic cleavage of the β–O–4 bonds; thus the ELs show significantly lower amounts in the range of 24.7–37.0/100 Ar compared to their MWL counterpart (66/100 Ar).^66^ Noteworthy, partial hydrolysis of β–O–4 units occurs during the hydrothermal treatment carried out prior to REx.^53,54^ β–5 bonds showed a less intense loss, ranging from 1.8 to 2.4/100 Ar (entry 9) compared to a prevalence of 3/100 Ar in silver birch MWL.^66^ Similarly, β–β structures showed only a minor decrease (7.0–7.6 and 9/100 Ar for EL and MWL,^66^ respectively) when using EtOH or AcOH. In contrast, a pronounced loss of β–β moieties (2.3–3.1/100 Ar, entry 10) was observed when using HCOOH, indicating a formic acid-catalyzed cleavage of either C–O or C–C bonds in β–β structures.

Summing up the amounts of β–O–4, β–5, and β–β structures, AcOH-extracted ELs retained more of their native bonds (43.5–46.4/100 Ar) than EtOH (33.6–36.2/100 Ar) or HCOOH (28.8–36.2/100 Ar) compared to MWL (78/100 Ar).^66^ Such discrepancies are ultimately related to the isolation of different lignin fractions and to the occurrence of degradation and/or condensation reactions during REx. For EtOH, a lower β–O–4 content correlated with a lower molar mass compared to AcOH (Figure 4A and Table 3). In contrast, HCOOH did not show such a correlation, instead the molar mass was comparable to AcOH. This could indicate that, in the case of HCOOH, the formation of new linkages (i.e., condensation reactions) has compensated for the degradation of β–O–4 bonds. However, the presence of coextracted hemicelluloses in the HCOOH samples could also distort their distribution toward higher molar masses.

**Table 3:** Results of SEC-MALS_785 nm_-RI and SEC-MALS_488 nm_-RI Analyses of Lignin (EL) and Cellulose (CS) Samples, Respectively, Extracted under Different Conditions^a^

entry	sample	*M*_n_ (kDa)	*M*_w_ (kDa)	*M*_*z*_ (kDa)	Đ
1	EL-1	1.500	2.805	5.000	1.87
2	EL-2	1.625	3.345	7.535	2.06
3	EL-3	2.970	13.780	72.300	4.64
4	EL-4	2.755	10.380	53.870	3.77
5	EL-5	2.835	11.760	93.260	4.15
6	EL-6	3.055	12.370	94.010	4.05
7	CS-1	n.a.	n.a.	n.a.	n.a.
8	CS-2	30.97	339.5	1004	10.96
9	CS-3	54.74	470.3	1240	8.59
10	CS-4	30.02	331.9	1053	11.06
11	CS-5	56.57	277.9	660.7	4.91
12	CS-6	24.21	166.5	497	6.88

a n.a. = not available.

**Figure 4:**
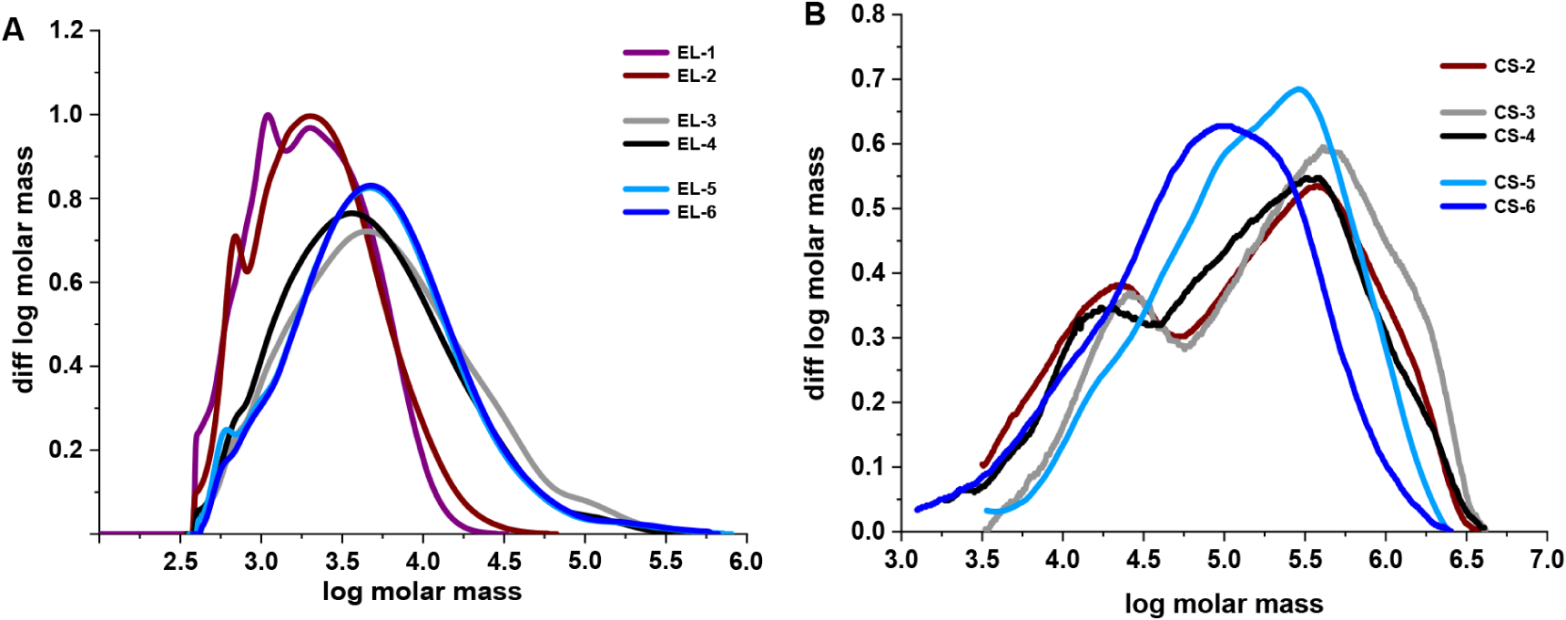
Molar mass distribution profiles of ELs (A) and CSs (B) measured by SEC-MALS_785 nm_-RI in DMSO/LiBr and SEC-MALS_488 nm_-RI in DMAC/LiCl, respectively. The CS-1 sample was not measured due to incomplete solubility.

Certainly, as mentioned, condensation and elimination reactions affected the structure of all ELs during extraction and during the initial hydrothermal treatment, irrespective of their molar mass. For instance, vinyl–aryl and vinyl–alkyl bonds—the products of dehydration reactions—were present in all the ELs (4.7–12.4/100 Ar and 3.7–9.7/100 Ar, respectively; entries 13–14). EtOH showed a pronounced presence of these new structures compared to AcOH, while HCOOH showed a lower presence. The amount of benzaldehyde-type structures (Ar–CHO) increased for EtOH and AcOH ELs (3.7–5.0/100 Ar) compared to MWL (3/100 Ar).^66^ In contrast, α-oxidized β–O–4 structures were less common in all ELs (0.4–1.4/100 Ar) than in MWL (3/100 Ar).

^31^P NMR gave additional insights into the OH group composition and structure of the ELs (Table 4). Unfortunately, the values determined for HCOOH are not reliable due to the pronounced presence of polysaccharides (i.e., overestimated aliphatic and underestimated phenolic OH). Under acidic conditions, the cleavage of β–O–4 bonds depletes aliphatic OH groups while producing new phenolic OH end groups. Concurrently, condensation and elimination reactions alter the OH group content and composition of the lignin as well. For EtOH and AcOH, the aliphatic-to-phenolic OH ratio was close to 1 (0.89–1.10), and their ratio between C5-substituted (=condensed + S-units) and C5-free (=catechols + G-units) phenolic OH groups was about 3.2, which was notably higher than the determined S/G ratios (2.6–2.9). For HCOOH, the C5-substituted-to-C5-free ratio was even higher around 4.7–4.8 compared to S/G ratios of 2.3–2.5, which was another good indication of a higher degree of condensation in this case. HSQC NMR was also used to determine the relative content of lignin carbohydrate complexes (LCCs) (structures **F** and **BE** in Figure 3, entries 20–21) and other carbohydrates.^53^ As mentioned, much higher amounts of carbohydrate moieties were found in HCOOH (127.6–139.6/100 Ar, entry 22) compared to EtOH and AcOH (5.3–6.1/100 Ar), which was confirmed by methanolysis (Figure S39 and Table S3). Evidently, the HCOOH extraction was much less selective toward lignin than EtOH and AcOH, as EL5–6 showed a carbohydrate content of ca. 22–23 wt % (Figure S39 and Table S3). The polysaccharides certainly underwent further degradation during the extraction, as indicated by the presence of furfural-type structures (i.e., Fur–CHO, Fur–Ox, entries 16–17, Figure 3).^53^ HSQC NMR indicated the presence of LCCs of the benzyl ether (BE) and γ-ester type in all ELs. However, for EtOH, signals for benzyl ether LCCs overlap with ethoxylated benzyl ether signals in the same region.^39^

**Table 4:** Functional Group Compostion of Extracted Lignins Evaluated from ^31^P NMR^a^

sample	aliphatic OH (mmol/g)	phenolic OH (mmol/g)	C5 subst. (mmol/g)	C5 free (mmol/g)	H-units (mmol/g)	total OH (mmol/g)	carboxylic OH (mmol/g)
EL-1	3.15	2.68	1.99	0.61	0.09	5.83	0.28
EL-2	2.51	2.93	2.18	0.66	0.10	5.44	0.17
EL-3	2.45	2.18	1.60	0.51	0.07	4.63	0.12
EL-4	2.62	2.70	2.01	0.60	0.09	5.32	0.14
EL-5	5.10	2.12	1.70	0.35	0.07	7.22	0.25
EL-6	4.17	1.99	1.59	0.34	0.07	6.17	0.27

a C5 subst. = phenolic OH of condensed and S-units. C5 free = phenolic OH of catechols + G-units.

### 2.3 Functionalization of Extracted Lignins

An important advantage of REx is the simultaneous functionalization of lignin during its extraction from biomass. In this way, REx enables the efficient production of lignins with favorable properties for material applications. In this study, the three tested solvents (supported by Amb-15) tailored the functionalization during lignin extraction: (i) EtOH for ethoxylation of benzylic α-OH groups, (ii) AcOH for acetylation of α- and γ-OH groups, and (iii) HCOOH for formylation of γ-OH groups. The occurring reactions are shown in Figure S41.

Ethoxylation was verified by the presence of the cross peak in HSQC NMR assigned to CH_3_– groups in ethoxy alkyl ethers at δ_H_/δ_C_ = 0.9–1.2/12.2–15.9 ppm^39^ (Figure 3B). However, it occurred only to a small extent, even when using the acidic catalyst Amb-15 (1.2–2.0/100 Ar; Table 2, entry 23). In comparison, in our recent work, we reported a degree of substitution of up to 40/100 Ar when using H_2_SO_4_ as a catalyst.^39^ Evidently, Amb-15 was unable to ensure the strongly acidic conditions needed for ethoxylation to occur, most likely due to concomitant mass transfer challenges of the H^+^ ions and too low acidity of the catalyst, as lignin functionalization often required stronger acid systems.^67^

In contrast, the higher acidity of the other two used solvents afforded functionalized lignins, with a similar low effect of Amb-15 in these cases most likely due to mass transfer/low acidity issues. Acetylation occurred extensively (39/100 Ar) when using AcOH, as supported by the presence of a strong peak in HSQC NMR at δ_H_/δ_C_ = 1.6–2.0/17.9–22.1 ppm assigned to the CH_3_– group in acetyl moieties (Figure 3D).^68^ In addition, the clusters at δ_H_/δ_C_ = 5.4–6.0/71.1–80.6 ppm and δ_H_/δ_C_ = 4.1–4.5/59.7–66.5 ppm confirmed the presence of acetylated β–O–4 moieties at the α- and γ-position, respectively.^68^ For AcOH, using Amb-15 as a catalyst led to higher amounts of α-acetylated β–O–4 structures which increased the degree of substitution from 0.27 to 0.40 (Table 2, entries 5–6). Acetylation of the β–O–4 γ-OH occurred at degrees of 0.19 and 0.13 for AcOH and AcOH + Amb-15, respectively. The total number of acetyl groups in β–O–4 moieties (15.4–19.8/100 Ar) did not match the total number of acetyl groups present in the lignin samples, suggesting that other lignin moieties with aliphatic or phenolic OH could have been acetylated as well. However, aliphatic and aromatic acetyl groups could not be distinguished due to signal overlap in the HSQC NMR spectra. Acetylation of polysaccharides could occur during the extraction and thus contribute to the total acetyl group content as well. Acetyl groups are also naturally present in mannans^69^ (*vide infra*: presence of sugars and lignin–carbohydrate complexes in ELs). Catalyst reusability was assessed using AcOH as the solvent; however, the catalytic activity of Amb-15 was found to be minimal already after the first recycle. The observed catalyst deactivation is most likely associated with ion-exchange processes between the Brønsted acidic H^+^ sites of the resin and metal cations (M^+^) naturally present in woody biomass.^70^

Formylation was verified by the presence of an intense peak in HSQC NMR at δ_H_/δ_C_ = 7.7–8.6/158.3–164.5 ppm, assigned to the CH– group in formate esters (Figure 3E). In contrast to acetylation, formylation solely occurred at the γ-position of β–O–4 structures supported by a broad peak at δ_H_/δ_C_ = 4.1–4.5/59.7–66.5 ppm, assigned to γ-formate esters.^71^ The absence of signals in the region δ_H_/δ_C_ = 4–5/60–67 ppm indicated that formylation did not occur at the α-position of β–O–4 structures. Using HCOOH, a higher degree of substitution could be reached compared to AcOH with approximately half of the β–O–4 structures being formylated in the γ-position (compare entries 3 and 7).

Overall, unexpectedly, the catalyst resulted in only minor functionalization of the lignin samples under the conditions investigated, most likely due to both mass transfer issues and the too low acidity of Amb-15.

### 2.4 Comparative Assessment of Different Protocols

A systematic comparison of different solvent-assisted lignin extraction and functionalization methods—representative literature protocols—employing ethanol, acetic acid, and formic acid has been performed. The comparison focuses on reaction conditions, lignin yield, preservation of β–O–4 linkages, and the degree of solvent-induced functionalization (Table 5).

**Table 5:** Comparison of Different Organosolv Processes for Biomass Fractionation Mediated by EtOH, AcOH, and HCOOH

						yield (wt %)			
entry	solvent	ref	solvent/conditions	*T* (°C)	*t* (h)	initial lignin	initial biomass	β–O–4 content	functionalization/degree
1	EtOH	^72^	4:1 EtOH/H_2_O; 0.24 M HCl; walnut shells	80	72	32		37 (per 100 Ar)	α-ethoxylation/n.r.
2		^73^	95:5 EtOH/H_2_O; 0.2 M HCl; beech, walnut and Douglas fir	reflux	6		beech: 4.1	beech: 63	α-ethoxylation/65–84%
							walnut: 6.1	walnut: 58	
							Douglas fir: 5.5	Douglas fir: 52	
3		^39^	hydrothermal treatment of birch then EtOH:H_2_O (70–99%); H_2_SO_4_ (0.15–1.2 M)	reflux	0.5–8	27–52		26–34 (per 100 Ar)	α-ethoxylation/up to 40.8 (per 100 Ar)
4		this work	hydrothermal treatment of birch then EtOH (99%); Amb-15/cat.-free	reflux	1	45.6–46.9		24.7–27 (per 100 Ar)	ethoxylation/1.2–2.0 (per 100 Ar)
5	AcOH	^74^	AcOH:H_2_O = 80:20 (v/v); 3.5% H_3_PO_2_; birch	150	0.5–2	56–65		9–14 (per C_900_)^75^	acetylation/n.r.
6		this work	hydrothermal treatment of birch then AcOH (99%); Amb-15/cat.-free	reflux	1	58.6–69.6		33.5–37 (per 100 Ar)	acetylation/39.2–39.6 (per 100 Ar)
7	HCOOH	^76^	step 1: HCOOH:H_2_O = 88:12 (v/v)	100 (both steps)	3 (each step); total = 6	19.7 (EtOAc-soluble fraction)		n.r.	formylation/n.r.
			step 2: HCOOH:H_2_O = 70:30 (v/v)						
8		^77^	formic acid extraction of steam-explosion lignin residue	reflux	0.5	18–65		n.r.	formylation/n.r.
9		this work	hydrothermal treatment of birch then HCOOH (99%); Amb-15/cat.-free	reflux	1	105–109^a^		24.7–30.9 (per 100 Ar)	formylation/12.6–15.4 (per 100 Ar)

a Corrected for the carbohydrate content.

Overall, the approach developed in this work offers advantages over previously reported strategies while also presenting some inherent limitations that merit discussion. A general advantage of this work is the use of mild reaction conditions across three different solvent systems (EtOH, AcOH, and HCOOH). Hence, in all cases, lignin extraction was carried out under reflux for 1 h, without strong acid catalysts, while literature protocols generally require longer reaction times (6–72 h), mineral acids such as HCl, and higher temperatures (up to 150 °C), representing a significant advantage compared to previously reported works.

#### 2.4.1 Lignin Yields

Overall, our method provides competitive or superior lignin yields under reduced severity. The ethanol-based extraction affords lignin yields of ca. 46 wt % which are comparable to or even higher than literature protocols involving strong acids and/or harsher conditions (27–52 wt %; compare entries 1–3 with 4). Using AcOH, our protocol delivered yields up to ca. 70 wt % (entry 6), slightly higher than the selected literature case (up to 65 wt %; entry 5) which was carried out at *T* = 150 °C. In formic acid, yields exceeding 100 wt % (complete delignification, *vide supra;* entry 9) indicate the effectiveness of the designed protocol to achieve high lignin yields while preserving a certain amount of β–O–4 linkages (25–30 per 100 Ar). This outcome is rarely achieved in conventional acidic systems, where uncontrolled degradation frequently happens when quantitative delignification occurs.^78^ Hence, in the two selected literature examples—where formylated lignins possessing a comparable number of β–O–4 linkages were isolated—lignin yields were ca. 2–5 times lower than in our work (18–65 wt %; compare entries 7–8 with 9).

#### 2.4.2 β–O–4 Linkages

Good preservation of β–O–4 linkages in the range of 25–37 per 100 aromatic units was achieved through our protocol, independent of the used solvent. For instance, this exceeds those reported for the selected AcOH fractionation method, where 9–14 linkages per C_900_ ^78^ were achieved (entry 5). Unfortunately, a thorough comparison was not accessible, as in some cases the number of β–O–4 linkages was not reported.

#### 2.4.3 Functionalization

Our approach enables efficient and solvent-specific functionalization, with extensive acetylation and formylation achieved using AcOH and HCOOH. We quantified acetyl and formyl groups in the ranges of 39–40 and 12–15 per 100 Ar, respectively (entries 6 and 9), while typical values were generally not reported in the literature. Conversely, ethoxylation occurred only to a minor extent via our REx approach (1–2 EtOH per 100 Ar; entry 4), which was significantly lower than our previously reported protocol (40.8; entry 3) and another literature example (65–85% degree of α-substitution in β–O–4 structures; entry 2) that both used H_2_SO_4_ as a strong homogeneous catalyst.

#### 2.4.4 Limitations

The REx method that we propose presents some limitations as well. In our approach, an initial hydrothermal treatment is needed prior to solvent extraction to facilitate lignin extraction. Only a subtle effect of the catalyst on lignin functionalization was observed, most likely due to H^+^ ion transfer limitation (poor stirring) and/or insufficient acidity of the catalyst. This aspect will be further investigated in the follow-up studies.

Overall, despite these limitations, the protocol developed in this work represents a highly efficient and versatile approach for lignin extraction and functionalization under mild conditions with or without the use of heterogeneous catalysis.

### 2.5 Chemical Structure of Cellulosic Solids

#### 2.5.1 Molar Masses and Molar Mass Distributions of Cellulosic Solids

REx affected the residual cellulosic solids as well and caused structural changes dependent on the used solvent and the presence of Amb-15 as a catalyst. For instance, the CS after AcOH and EtOH extraction showed a broad bimodal distribution (Figure 4B); the low-molar-mass fraction was typically related to the presence of xylan. In contrast, the solids obtained after HCOOH extraction show a very different molar mass distribution. In the absence of a catalyst, the profile is slightly trimodal, which is typical for pulps produced under acidic conditions, like sulfite pulps, and is related to the degradation of xylan.^79^ NMR analyses of the solids further supported this, indicating also lower amounts of hemicelluloses in the case of HCOOH.

For all solvents, with Amb-15 present, the longer chains suffer considerably and the DP_w_ drops to about 1000. In contrast to the minor effects observed on the yields of the lignin fractions, the addition of the catalyst heavily impacted the molecular weight distributions of the isolated cellulosic solids. The loss of hemicelluloses and the more pronounced cellulose degradation during HCOOH extraction indicate the pronounced hydrolysis of glycosidic bonds caused by the stronger acidity of formic acid compared to AcOH and EtOH. A partial removal of hemicelluloses through HCOOH treatment was also supported by solution-state NMR. The relative intensities of the peaks associated with hemicellulose decreased in the diffusion-edited ^1^H NMR spectra (see Figure S32). On the basis of the molar mass distribution alone, an application scenario cannot be projected, but the DP range of 2000–3000 for the milder variants could find application in paper or packaging.

#### 2.5.2 Functionalization of Cellulosic Solids

Polysaccharides exhibit a large number of OH groups that are potentially accessible for ethoxylation with EtOH or esterification with AcOH and HCOOH. Modification of OH groups greatly impacts the material properties and is, thus, of critical importance for applications.^80,81^ We therefore attempted to determine the extent of functionalization in cellulosic solids after extraction.

Both solid-state ^13^C NMR measurements and solution-state NMR experiments after dissolution in a [P_4444_]­[OAc]:DMSO-*d*_6_ (1:4 wt %) electrolyte were performed.^82^ The determination of functionalization relied on the qualitative and semiquantitative information available from the diffusion-edited ^1^H and solid-state ^13^C NMR experiments, since no quantitative information could be obtained due to strong peak superposition with hemicellulose resonances and the high viscosity of the samples. Since the cellulosic solids showed only partial solubility in the applied NMR electrolyte—presumably due to the minor presence of residual wood or insoluble lignin—an additional bleaching step was conducted to produce a purer, dissolvable fraction (bleached cellulosic solids) as described previously.^39^

First, ethoxylation of the cellulosic solids was not observed when using Amb-15 as an acidic catalyst. In our previous REx study, ethoxylation of the cellulosic solids was observed, albeit only under severe reaction conditions with high H_2_SO_4_ concentrations and high temperatures.^39^ In contrast, the use of organic acids promoted esterification of the cellulosic solids, as signals assigned to acetyl or formate esters emerged in the diffusion-edited ^1^H NMR spectra (Figure 5). For AcOH-treated CS, a signal emerged in the acetate spectral region at 2.02 ppm (Figure 5A). This peak was recently assigned to a regioselective modification of cellulosic C6-OH moieties.^83^ However, this peak was directly superimposed with acetate signals already present in the blank spectrum before AcOH treatment (Figure 5A). These are likely caused by naturally occurring partially acetylated xylan structures. Consequently, it was not possible to distinguish if the additional acetylation occurred on the cellulose or hemicellulose backbone. The intensity of the peaks increased when using Amb-15, suggesting that the catalyst promoted esterification, in contrast to what was observed for lignin substrates. This behavior can be rationalized by the distinct responses of carbohydrates and lignin to acid catalysis. Hence, while carbohydrates are generally activated by moderate Brønsted acidity,^84,85^ effective lignin functionalization typically requires stronger or more aggressive acid systems due to its inherent recalcitrance.^67^ With HCOOH, formate esters showed one sharp singlet around 8.24 ppm in the diffusion-edited ^1^H NMR spectra (Figure 5B). Free HCOOH was also observed in the ^1^H NMR spectra, as a strong peak at 8.61 ppm,^82^ but not in the solid-state NMR spectra. It was reported that cellulose formate esters are partially unstable at elevated temperatures,^86^ and thus the cleavage of the bond during solution-state NMR measurements at 65 °C appears possible. Moreover, saponification in the alkaline electrolyte with residual H_2_O in the system is conceivable. Given that follow-up reactions of the formate esters are likely during the dissolution step, we are reluctant to assign the peak at 8.24 ppm to a certain preferential modification during the REx step. Unfortunately, the high viscosity of the NMR samples did not allow us to get further insights on this issue from ^13^C or 2D NMR experiments.

**Figure 5:**
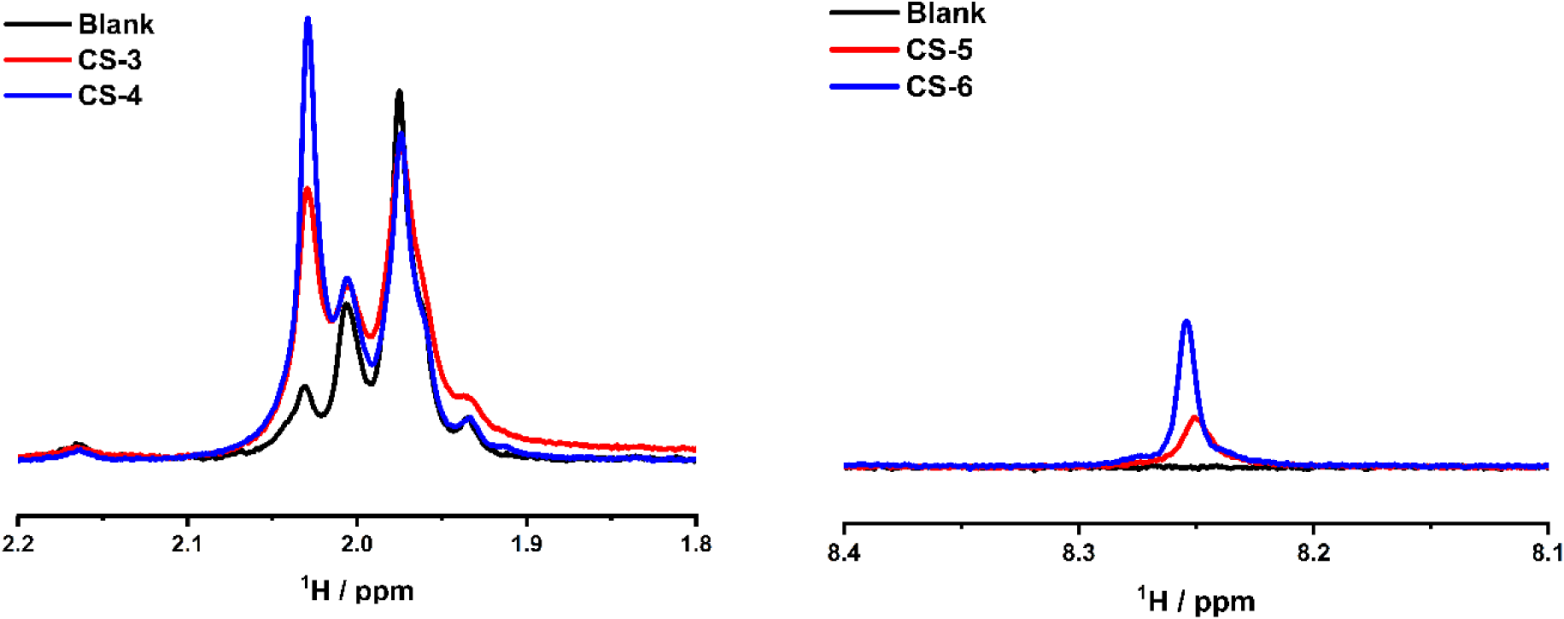
Overlay of diffusion-edited ^1^H NMR spectra of the bleached CS obtained from the treatments with AcOH (left) and HCOOH (right). In both cases, a reaction with the carboxylic acid was apparent, either by an increase in the resonance around 2.02 ppm (acetyls) or by the emergence of a peak around 8.24 ppm (formyls). Addition of the catalyst increased the relative intensity. No other significant changes were apparent in the spectra (see the SI).

Overall, the NMR study showed that the residual cellulosic solids were affected by the REx protocol to a small degree. Esterification of the cellulose and/or hemicellulose constituents was observed irrespective of using Amb-15, but peak intensities increased when the catalyst was present. However, we were not able to obtain quantitative information on the degree of substitution of the modified cellulosic solids, but it could be expected to be minimal on the basis of ^1^H NMR. While the rather strong peaks in the diffusion-edited ^1^H NMR experiments confirm a covalent modification, caution is advisable when interpreting the spectra on a quantitative basis. This NMR experiment strongly overestimates the signals of minor constituents, which is especially relevant when investigating complex and chemically heterogeneous samples like the CS analyzed herein.

## 3 Conclusions

Reactive extraction proved to be a flexible approach for the simultaneous fractionation and functionalization of lignin and cellulosic solids under mild conditions. The choice of solvent-reagent (i.e., EtOH, AcOH, HCOOH) used in REx determined the extraction severity and selectivity, thereby decisively influencing lignin yield, purity, molecular structure, molar mass, and functionality. With AcOH and HCOOH, lignin esterification occurred to a degree of substitution of up to 0.5 or higher. Though the use of Amberlyst 15 as a heterogeneous catalyst in REx was slightly effective for lignin functionalization especially when using AcOH as the solvent (possibly due to mass transfer issues, too weak acidity, or too mild conditions), it clearly modified the structure and molar masses of the residual cellulosic fractions. Hence, albeit derivatization of the cellulosic solids appeared to be minimal, degradation by acidic hydrolysis was pronounced in the presence of the catalyst, and even more pronounced when using HCOOH. Overall, the isolated functionalized lignin and cellulose streams may find application in specific downstream material applications (e.g., thermoplastics, coatings, etc.) or as substrates for further depolymerization to monomers considering the relatively high content of β–O–4 linkages. We hope the setup developed in this work may inspire future studies on the use of different heterogeneous catalysts for the simultaneous fractionation and functionalization of lignin and cellulose under mild conditions.

## 4 Experimental Section

### 4.1 General

VTT Technical Research Centre of Finland (Espoo, Finland) kindly supplied silver birch (*Betula pendula*) wood chips. The wood chips were finely ground and screened into sawdust (0.55–0.15 mm particle size) as described in our recent paper.^39^ The acidic cation exchange resin Amberlyst 15 (Amb-15, >50 mesh) used as a catalyst, was purchased from Sigma-Aldrich (Merck). Ethanol (EtOH; >99%), formic acid (HCOOH, >99%), acetic acid (AcOH, >99%), acetone, dimethyl sulfoxide (DMSO, HPLC grade), DMSO-*d*_6_, CDCl_3_, pyridine, *N*,*N*-dimethylacetamide (DMAc, HPLC grade), 2-chloro-4,4,5,5-tetramethyl-1,3,2-dioxaphospholane, and LiBr were purchased from Sigma-Aldrich (Merck) and used without any further purification. LiCl was purchased from VWR International (Avantor). The direct dissolution electrolyte (tetra-*n*-butylphosphonium acetate [P_4444_]­[OAc]: DMSO-*d*_6_, 1:4 wt %) for solution-state NMR of the cellulosic fractions was either prepared according to published procedures^82^ or sourced from Innotope.

#### 4.1.1 Hydrothermal Treatment (HTT) of Birch Wood

Prior to reactive extraction, an HTT of birch wood was performed as described earlier.^39,53,54^ In brief, birch wood sawdust (31.6 g with 4.9% moisture content corresponding to 30.0 g of dry wood) was placed inside a stainless-steel autoclave together with the required amount of water (28.5 g) to reach a liquid-to-solid ratio (L/S) of 1. The reactor was then closed and set to react until the desired process severity (P-factor) of 500 was reached (*T* = 180 °C; *t* ∼ 40 min). More details about the P-factor calculation are reported in our previous works.^53,54^ Once the reaction was complete, the reactor was cooled down fast (from 180 °C to *T* < 100 °C within 2 min) and the treated solids were exhaustively washed with deionized water. The hydrothermally treated solids (P-factor = 500) were labeled S-500.

#### 4.1.2 Amberlyst-15 Catalyzed Reactive Extraction

REx was performed under different conditions with the apparatus depicted in Figure 2A. The experimental design was chosen to maintain the conditions mild and simple. In a typical experiment, a round-bottom flask was filled with S-500 (4 g dry matter), the solvent-reagent of choice in an amount able to provide consistent reflux conditions (EtOH, HCOOH, or AcOH; purity >99%; 50 mL), and Amb-15 as the catalyst (0.8 g; 20 wt %). The catalyst was placed in a stainless-steel basket to facilitate its removal from the mixture after the reaction. The catalyst basket was prepared by folding a stainless-steel net (mesh 0.3 mm) into a cylindrical shape. The mixture was refluxed for 1 h. Then, the cellulosic solids (CS) were filtered through a glass crucible and exhaustively extracted with aqueous acetone (50 mL, 75% aq. acetone) to maximize the lignin yield.^39,53,54,87^ The extracted lignin (EL) was isolated by rotary evaporation and its yield was evaluated gravimetrically. ELs were analyzed by 2D HSQC NMR, ^31^P NMR, and SEC–MALLS. The following formula has been applied to calculate the lignin yield with respect to its initial content in biomass:

$$\mathrm{Lignin}\;\mathrm{yield}\;(\%\;\mathrm{initial}\;\mathrm{content})=\frac{\mathrm{EL}\;\mathrm{yield}\;(\mathrm{wt}\;\%)}{\mathrm{lignin}\;\mathrm{content}\;\mathrm{in}\;\mathrm{biomass}\;(\%)}$$

where EL yield is the yield of the lignin fraction with respect to the dry wood biomass and the lignin content in biomass (%) is the percentage of lignin in woody biomass evaluated via compositional analysis (see SI). Each experiment was performed once.

### 4.2 Analysis of the Extracted Lignins

#### 4.2.1 Nuclear Magnetic Resonance (NMR) Spectroscopy

*HSQC NMR method.* The 2D NMR spectra were recorded on a Bruker AVANCE 600 NMR spectrometer equipped with a CryoProbe following a procedure previously adopted by our group.^39,53,54^ About 30 mg of the sample were dissolved in 0.6 mL of DMSO-*d*_6_. The acquisition time of 77.8 ms was set for the ^1^H-dimension, and 36 scans per block were collected using the 1024 collected complex points. For the ^13^C dimension, the acquisition time was 3.94 ms and 256 time increments were recorded. The 2D HSQC NMR data were manipulated with 1024 × 1024 data points applying the Qsine function for both ^1^H and ^13^C dimensions. The DMSO peak at δ_C_/δ_H_ = 39.5/2.49 ppm was used for the calibration of the chemical shifts. The cross-peaks were assigned based on previous reports.^88–91^ The quantity of different lignin and LCC signals was normalized using an assumption of:


$${G\;+\;S=G}_{2}{+S}_{2,6}/2=100\mathrm{Ar}$$

This assumption implies that the condensation (substitution) at the positions of G_2_ and S_2,6_ in lignin is insignificant. However, this is still valuable for relative comparison with literature data, as this normalization is used when only HSQC spectra of lignins are available. HSQC spectra are reported in the SI.

^*31*^ *P NMR method.* The amounts of different hydroxyl groups were determined by ^31^P NMR spectroscopy^92^ in accordance with the literature.^93^ Spectra were recorded on a Bruker AVANCE III HD 400 NMR spectrometer. Dry lignin samples (40.0 mg) were completely dissolved in a CDCl_3_/pyridine solution (0.4 mL, 1:1.6 v/v) in a 1.5 mL glass vial. Endo-N-hydroxyl-5-norbornene-2,3-dicarboximide (e-HNDI) was added as internal standard (100 μL, c = 20 mg/mL in CDCl_3_/pyridine, 0.3 μmol/mg lignin). Chromium­(III) acetylacetonate (Cr­(acac)_3_) was used as relaxation agent (50 μL, c = 11.4 mg/mL in CDCl_3_/pyridine). Prior to the ^31^P NMR measurement, 100 μL of phosphitylation reagent (2-chloro-4,4,5,5-tetramethyl-1,3,2-dioxaphospholane) was added to the mixture and shaken vigorously. The acquisition time and the relaxation delay were 1 and 5 s, respectively; 128 scans were collected. ^31^P NMR spectra were evaluated according to the literature^92,93^ and can be found in the SI.

#### 4.2.2 Molar Mass Characterization of Lignin by SEC-MALS_785 nm_-RI in DMSO/LiBr (0.5% w/v)

The molar mass characterization was done according to Zinovyev et al.^94^ In brief, 10 mg of the dry lignin samples were dissolved in 1 mL of the eluent (DMSO/LiBr, 0.5% w/v) and filtered through a 0.45-μm PTFE syringe filter prior to the measurement. The following SEC setup was used for analysis: a G1312A HPLC pump and a G1313A autosampler (both from Agilent Technologies, Waldbronn, Germany), and a Dawn Heleos II MALS detector, λ = 785 nm, and an Optilab T-rEX differential refractive index detector, λ = 660 nm (both from Waters|Wyatt Technology, Santa Barbara, CA, USA). For the separation, a PolarGel M guard column (7.5 × 50 mm) and three PolarGel M columns (7.5 × 300 mm, 5 μm particle size) were used. The SEC system was operated under the following conditions: 0.5 mL/min flow rate; 5 μL injection volume; 65 min run time; 35 °C column temperature. DMSO/LiBr (0.5% w/v), filtered through a 0.02-μm filter, was used as the eluent. The data evaluation was done with Astra 7.1 software (version 7.3.0, Waters|Wyatt Technology, Santa Barbara, CA, USA).

#### 4.2.3 Carbohydrate Content and Composition by GC–FID after Acidic Methanolysis

EL2-6 were subjected to acidic methanolysis according to the literature.^61,62^ EL1 was excluded because it was not available for repeat measurements or validation, and its inclusion would have prevented reproducibility and compromised data quality. Prior to analysis, the samples were thoroughly dried in a vacuum oven at 40 °C and stored under an inert atmosphere. In brief, 15 mg of sample were digested in 2 M acidic MeOH for 4 h at 100 °C before quenching with pyridine and evaporating under N_2_-sparging. After drying, an internal standard (sorbitol) was added to the sample before dissolution in pyridine and silylation using TMCS and BSTFA (10% TMCS) overnight. Derivatized samples were diluted with EtOAc and centrifuged prior to injection into the GC-FID system. Digestions were performed in triplicate. Xylan standards were subjected to the same conditions to determine recovery rates.

The GC–FID system (7890B, Agilent Technologies, USA) was equipped with an HP-1 capillary column (30 m × 0.32 mm ID, 0.25 μm film thickness, Agilent Technologies, USA). High-purity hydrogen gas (5.0) was used as the mobile phase for separation. GC parameters: column flow rate, 0.8 mL/min at 0.97 bar; injector temperature, 250 °C; temperature gradient, 100 °C (1 min hold) followed by 4 °C min^–1^ to 170 °C, then 12 °C min^–1^ to 300 °C (7 min hold); detector temperature, 320 °C; sample injection in split mode (1:25), 10 μL aliquots were injected. Peak assignment, data acquisition, and quantification were performed using MSD ChemStation (Agilent Technologies, USA). Peak identification was done by comparison with retention times of authentic reference compounds. Quantification was done by calibration with authentic reference compounds.

### 4.3 Analysis of the Residual Cellulosic Solid Fraction

#### 4.3.1 Compositional Analysis

The compositional analysis of CS included the quantification of acid-insoluble lignin (AIL), acid-soluble lignin (ASL), and carbohydrate composition using standard protocols.^8^ A dry sample (ca. 0.3 g) was hydrolyzed with 3 mL of 72% H_2_SO_4_ at 30 °C for 1 h. The acid solution was then diluted to 4% H_2_SO_4_ concentration and autoclaved at 121 °C for 1 h. The amount of AIL was determined gravimetrically upon filtration, while ASL was evaluated by UV spectrophotometry at 205 nm (absorptivity 90–110 L·g^–1^·cm^–1^).^95^ The carbohydrate monomers released by the acid hydrolysis were analyzed using a Dionex ICS 3000A ion chromatography system equipped with a Dionex CarboPac PA1 column (both from Thermo Fisher Scientific Inc., Finland). The compositional analysis of CS is reported in Table S1.

#### 4.3.2 NMR Spectroscopy

*Solid-state* ^*13*^ *C*. cross-polarization/magic-angle spinning (CP/MAS) NMR spectra were recorded on a Bruker Avance III HD 400 spectrometer with a resonance frequency of 400.13 and 100.61 MHz for ^1^H and ^13^C, respectively, equipped with a 4 mm dual broadband CP/MAS probe. Data were acquired at a spinning rate of 12 kHz, with a CP contact time of 2 ms and SPINAL 64 ^1^H decoupling. Chemical shifts were referenced externally against the carbonyl signal of glycine at d = 176.03 ppm. Spectra are reported in the SI.

*Solution-state NMR spectra*. Spectra of the cellulosic bleached and unbleached solids were obtained after dissolution in a direct dissolution electrolyte on a Bruker NMR AV III 400 spectrometer at an acquisition temperature of 65 °C. The materials were characterized by quantitative ^1^H and diffusion-edited ^1^H experiments. The NMR samples were prepared in a [P_4444_]­[OAc]:DMSO-*d_6_* (1:4 wt %) electrolyte at a measuring concentration of 2.5 wt % closely following the reported protocol.^82^ Similar to our previous report, only partial dissolution was observed when dissolving the obtained raw cellulosic fractions.^39^ Only after bleaching, a completely soluble material was obtained. The recorded spectra are summarized in the SI.

#### 4.3.3 Molar Mass Characterization of Cellulose by SEC-MALLS_488 nm_-RI in DMAc/LiCl (0.9 w/v %)

To improve dissolution, samples containing cellulose (appr. 5–10 mg of dry weight per sample) were activated using a solvent exchange procedure from ethanol to DMSO.^96^ 1 mL of DMAc/LiCl (9% w/v) was then added to the sample and stirred for 24 h at room temperature. The samples were filtered through a 0.45-μm PTFE syringe filter and subjected to SEC analysis. The following SEC setup was used for the analysis: a G1312B HPLC pump and a G1367B autosampler (both from Agilent Technologies, Waldbronn, Germany), a Dawn DSP MALS detector with a diode laser, λ = 488 nm (Waters|Wyatt Technology, Santa Barbara, CA, USA), and a Shodex RI-71 refractive index detector (Showa Denko K.K., Tokyo, Japan). For the separation, an Agilent guard column (PL gel, 7.8 mm i.d., 50 mm length, 20 μm) and four SEC columns, Styragel HMW6E Mixed Bed, 20 μm, 7 × 300 mm (Waters|Wyatt Technology, Santa Barbara, CA, USA), were used as the stationary phase. Operating conditions: 1.0 mL/min flow rate, 100 μL injection volume, and 45 min run time. DMAc/LiCl (0.9%, w/v), filtered through a 0.02-μm filter, was used as the eluent. Data were evaluated using Astra 4.7, Grams 7, Access, and OriginPro 2020 software. Sample CS-1 could not be analyzed due to its partial solubility in the DMAc/LiCl solvent.

## Supplementary Material



## Notes

Additional information about the reaction conditions and compositional analysis for REx, HSQC, and ^31^P NMR spectra of the lignin samples, solid-state and solution-state NMR spectra of the cellulosic solids, carbohydrate content and composition of the lignin samples, corrected lignin mass balances and yields, and functionalization reactions during REx (PDF)
