# Supplementary material for: Simultaneous Lignin Fractionation and Functionalization under Mild Conditions via Reactive Extraction
Source: ACS Omega. 2026 Jul 17;11(30):44826–40. doi: 10.1021/acsomega.6c01031 (PMC13449205; doi:10.1021/acsomega.6c01031)
Supplement: Supplementary file 1 [file ao6c01031_si_001.pdf]

Electronic Supporting Information (ESI)

Simultaneous lignin fractionation and functionalization under mild conditions via reactive extraction

Davide Rigo[a,*], Nadine Kohlhuber[b], Lukas Fliri[a,b], Irina Sulaeva[c], Daryna Diment[a], Oliver Musl[b,*], and Antje Potthast[b]

[a]Department of Bioproducts and Biosystems, Aalto University, Vuorimiehentie 1, Espoo, 02150 (Finland)
[b]BOKU University, Department of Natural Sciences and Sustainable Resources, Institute of Chemistry of Renewable Resources, Konrad-Lorenz-Strasse 24, 3430 Tulln and der Donau, Austria
[c]BOKU University, Core Facility Analysis of Lignocellulosics (Alice), Konrad-Lorenz-Strasse 24, 3430 Tulln and der Donau, Austria

Contents

1.1. The reaction conditions and compositional analysis for REx 2
1.2. HSQC NMR spectra of the lignin samples 3
1.3. ^{31}P NMR spectra of lignin samples 6
1.4. Solid state NMR spectra of the cellulosic solids 7
1.5. Solution state NMR spectra of the cellulosic solids 10
1.6. Carbohydrate content and composition by GC–FID after acidic methanolysis 23
1.7. Corrected lignin mass balances and yields 24
1.8. Functionalization reactions during REx 25

1.1. The reaction conditions and compositional analysis for REx

Table S1. Summary table for the reaction conditions used to carry out REx and compositional analysis of the cellulose-rich residual solids (RS)

Entry	Label	Conditions		Compositional analysis (wt%)			
		Solvent	Catalyst	AIL	ASL	Total lignin	Total carbs
1	S-500	n.a.	n.a.	29.2	1.7	30.9	69.1
2	RS-1	EtOH	No	18.3	1.0	18.3	69.5
3	RS-2		Yes	20.3	1.8	22.1	71.9
4	RS-3	AcOH	No	15.0	1.6	16.5	76.1
5	RS-4		Yes	15.0	1.4	16.4	73.7
6	RS-5	HCOOH	No	8.9	0.8	9.7	79.3
7	RS-6		Yes	5.7	0.6	6.3	81.6

S-500: hydrothermally treated solids as the starting material for REx. n.a. = not applicable. AIL = acid insoluble lignin. ASL = acid soluble lignin.

1.1. The yield of extracted lignin, residual solids, and residual lignin

Table S2. The yields of extracted lignin (EL), residual solids (RS), and residual lignin (RL) obtained via the Amb-15 catalyzed REx

Entry	Label	Conditions		Yield (wt% of original biomass)[a]		Yield (wt% of original lignin)[b]	
		Solvent	Catalyst	RS	EL	RL	EL
1	RS-1	EtOH	No	85.5	14.5	50.7	46.9
2	RS-2		Yes	86.1	14.1	61.5	45.6
3	RS-3	AcOH	No	82.1	18.1	44.0	58.6
4	RS-4		Yes	81.3	21.5	43.0	69.6
5	RS-5	HCOOH	No	66.3	42.0	20.7	136.0
6	RS-6		Yes	67.1	43.3	13.6	140.2

[a]wt% yield of products with respect to the initial dry biomass (S-500). [b]wt% yield of lignin with respect to the initial lignin content in the biomass (S-500).

1.2. HSQC NMR spectra of the lignin samples

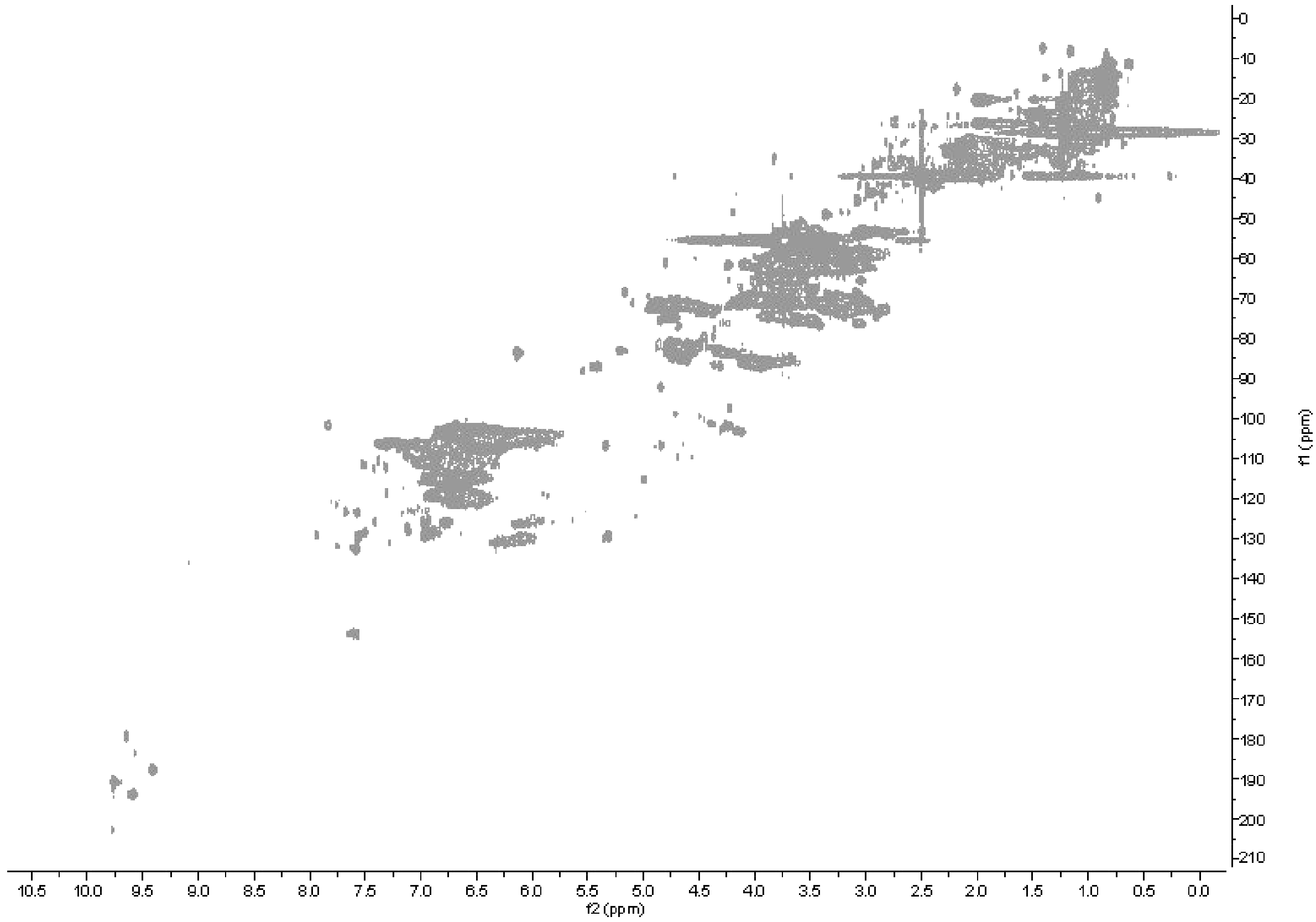

Figure S1. HSQC NMR spectrum of EL-1. Solvent: DMSO-d_6.

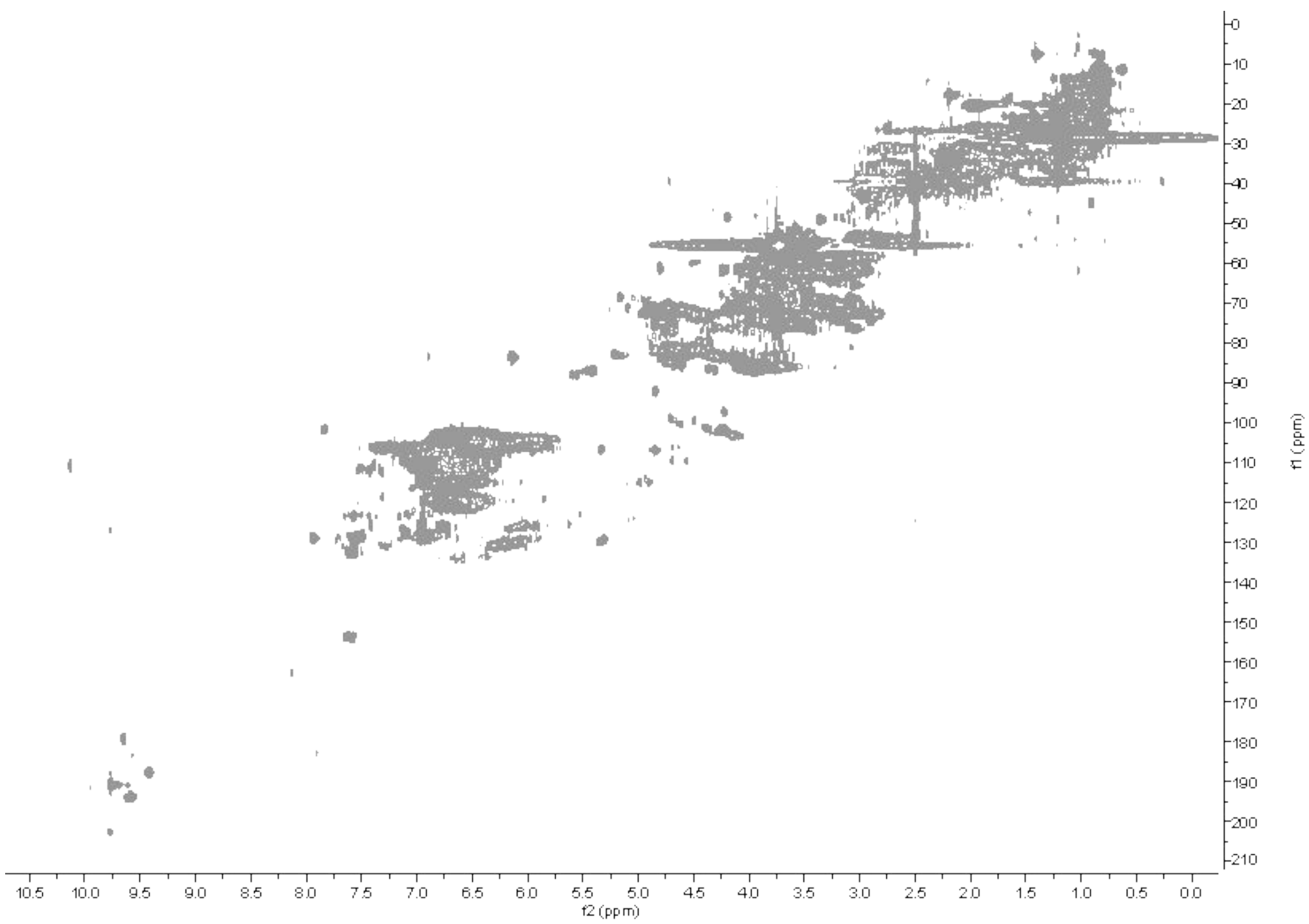

Figure S2. HSQC NMR spectrum of EL-2. Solvent: DMSO-d_6.

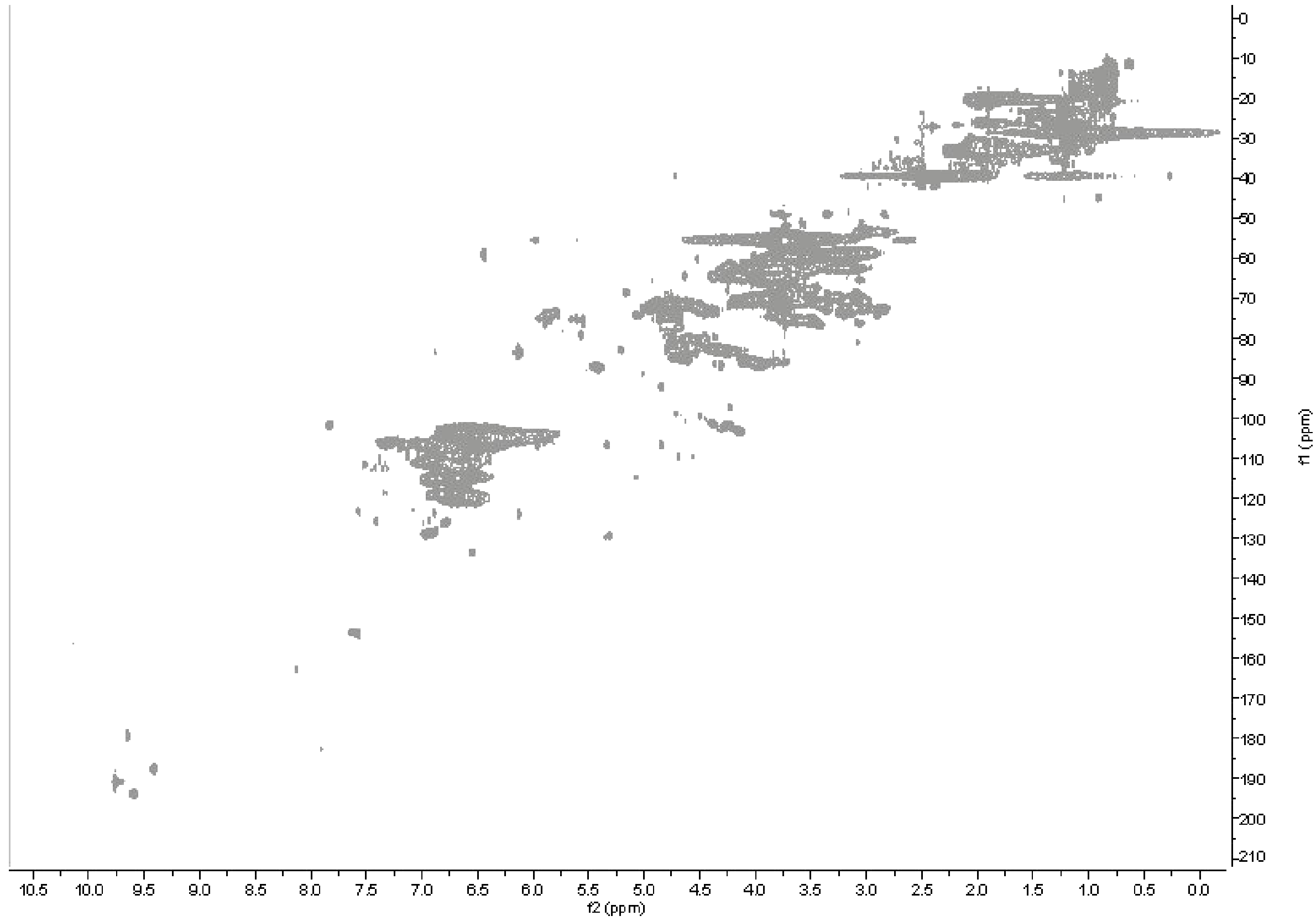

Figure S3. HSQC NMR spectrum of EL-3. Solvent: DMSO-d_6.

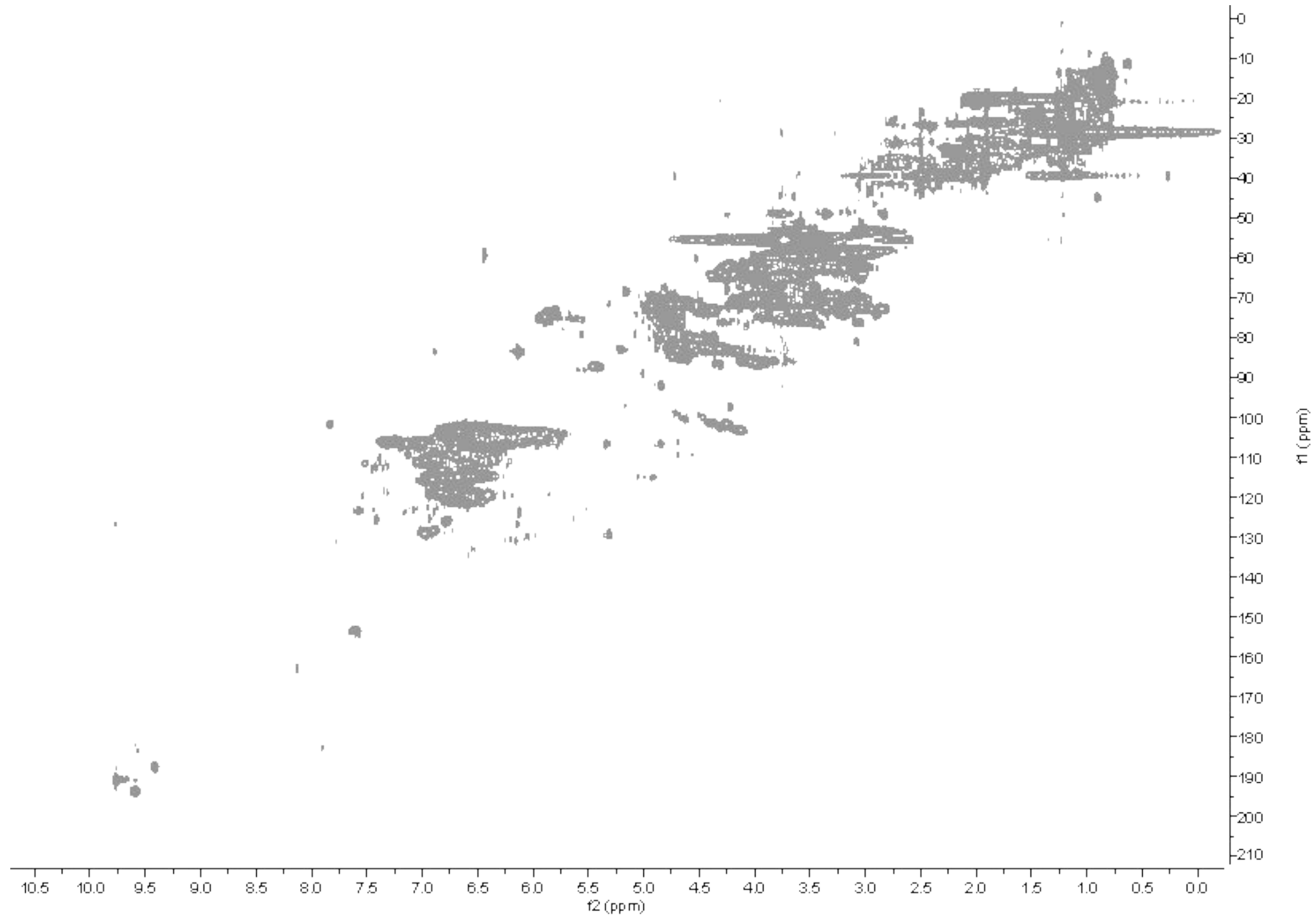

Figure S4. HSQC NMR spectrum of EL-4. Solvent: DMSO-d_6.

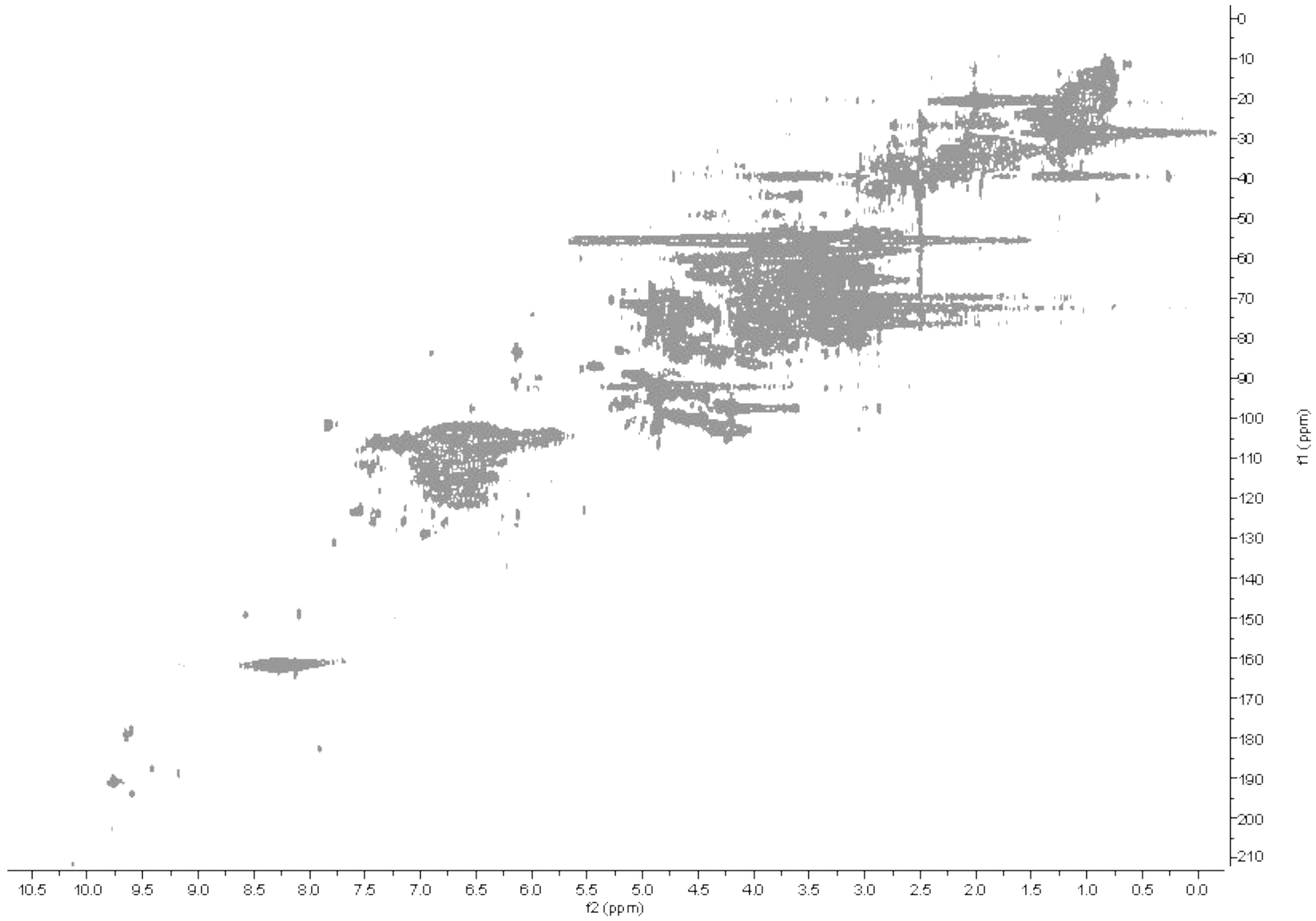

Figure S5. HSQC NMR spectrum of EL-5. Solvent: DMSO-d_6.

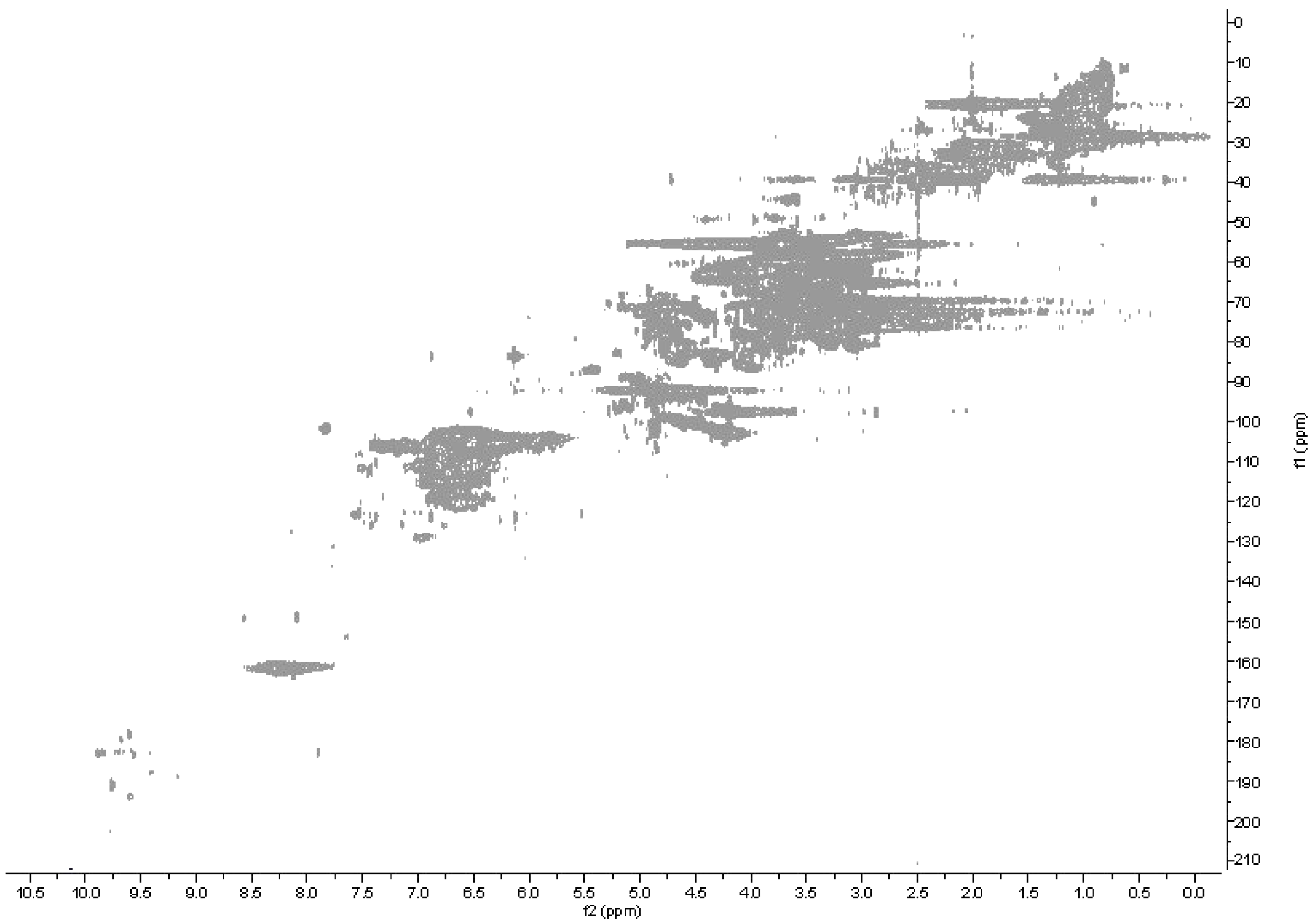

Figure S6. HSQC NMR spectrum of EL-6. Solvent: DMSO-d_6.

1.3. ^{31}P NMR spectra of lignin samples

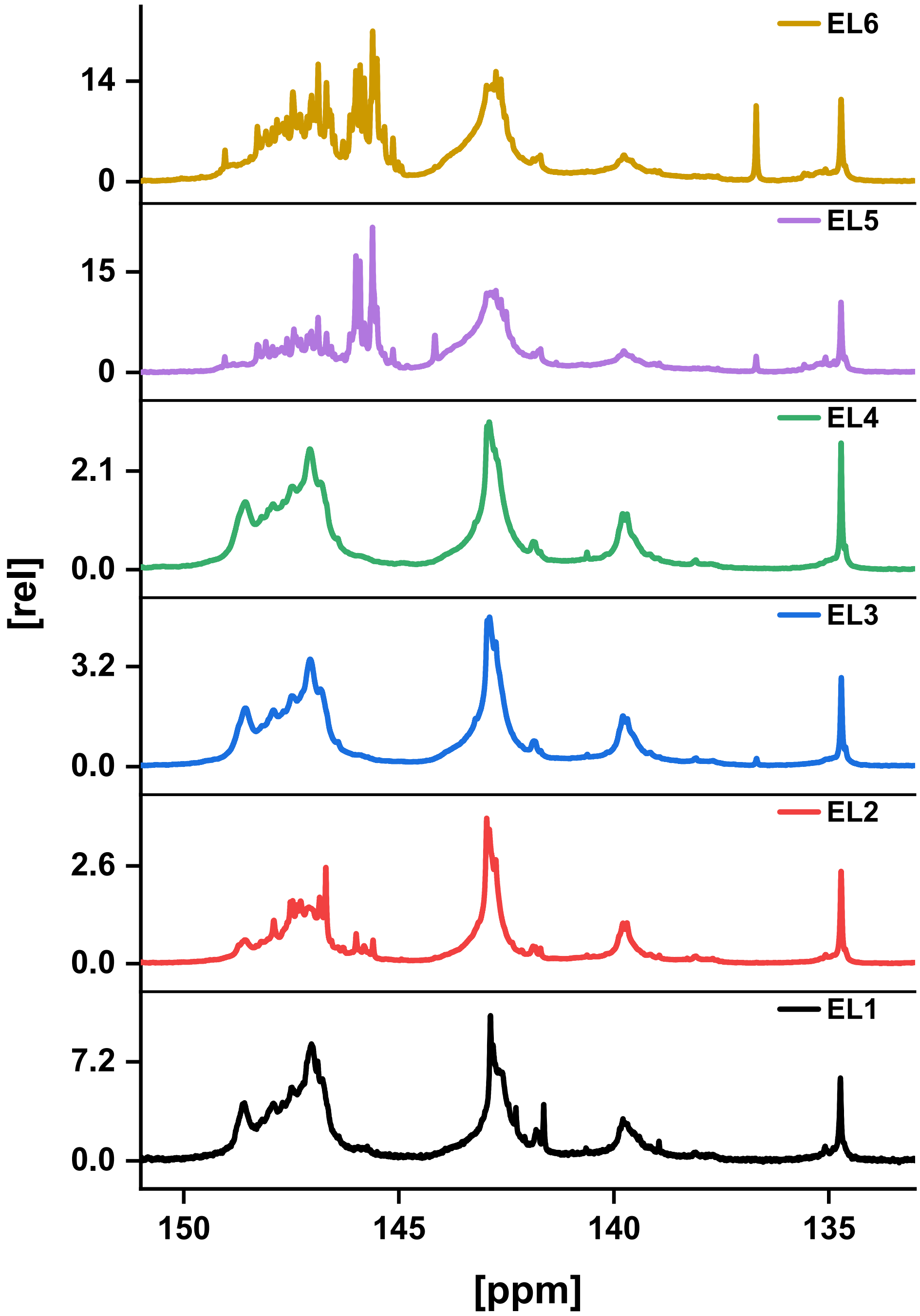

Figure S7. ^{31}P NMR spectra of lignin samples.

1.4. Solid state NMR spectra of the cellulosic solids

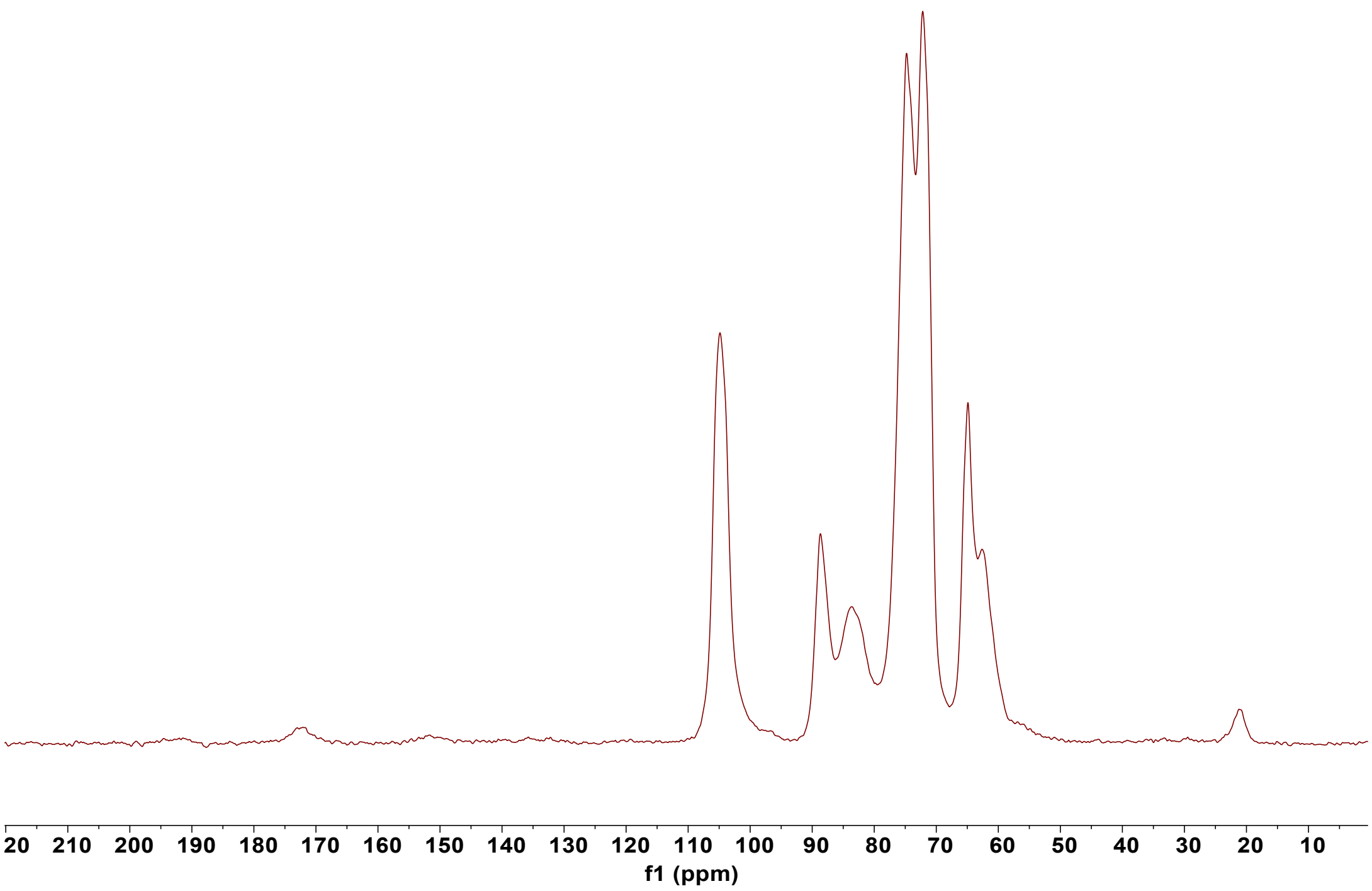

Figure S8. Solid state ^{13}C CP/MAS NMR spectrum (100 MHz) of CS-2.

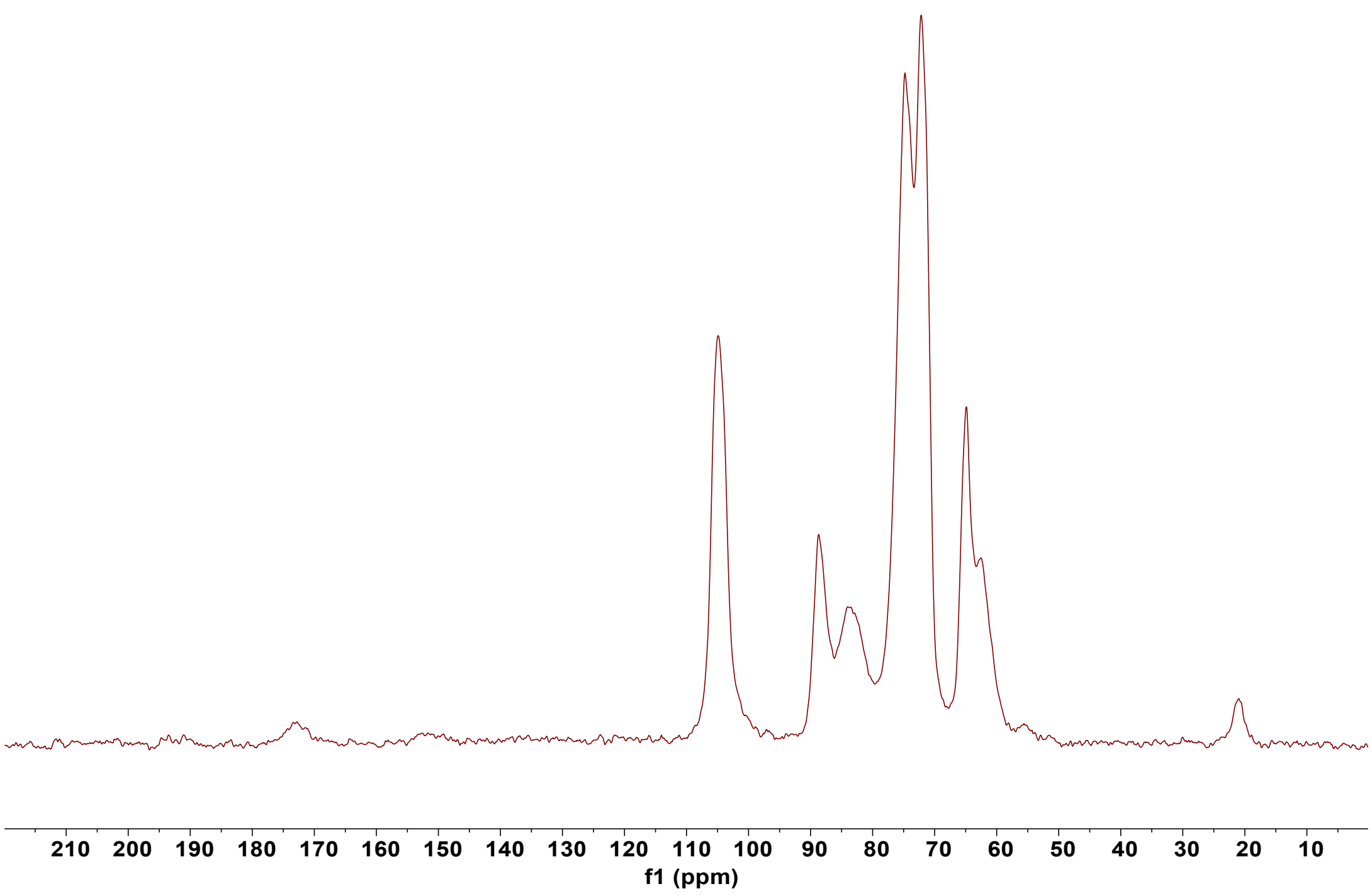

Figure S9. Solid state ^{13}C CP/MAS NMR spectrum (100 MHz) of CS-3.

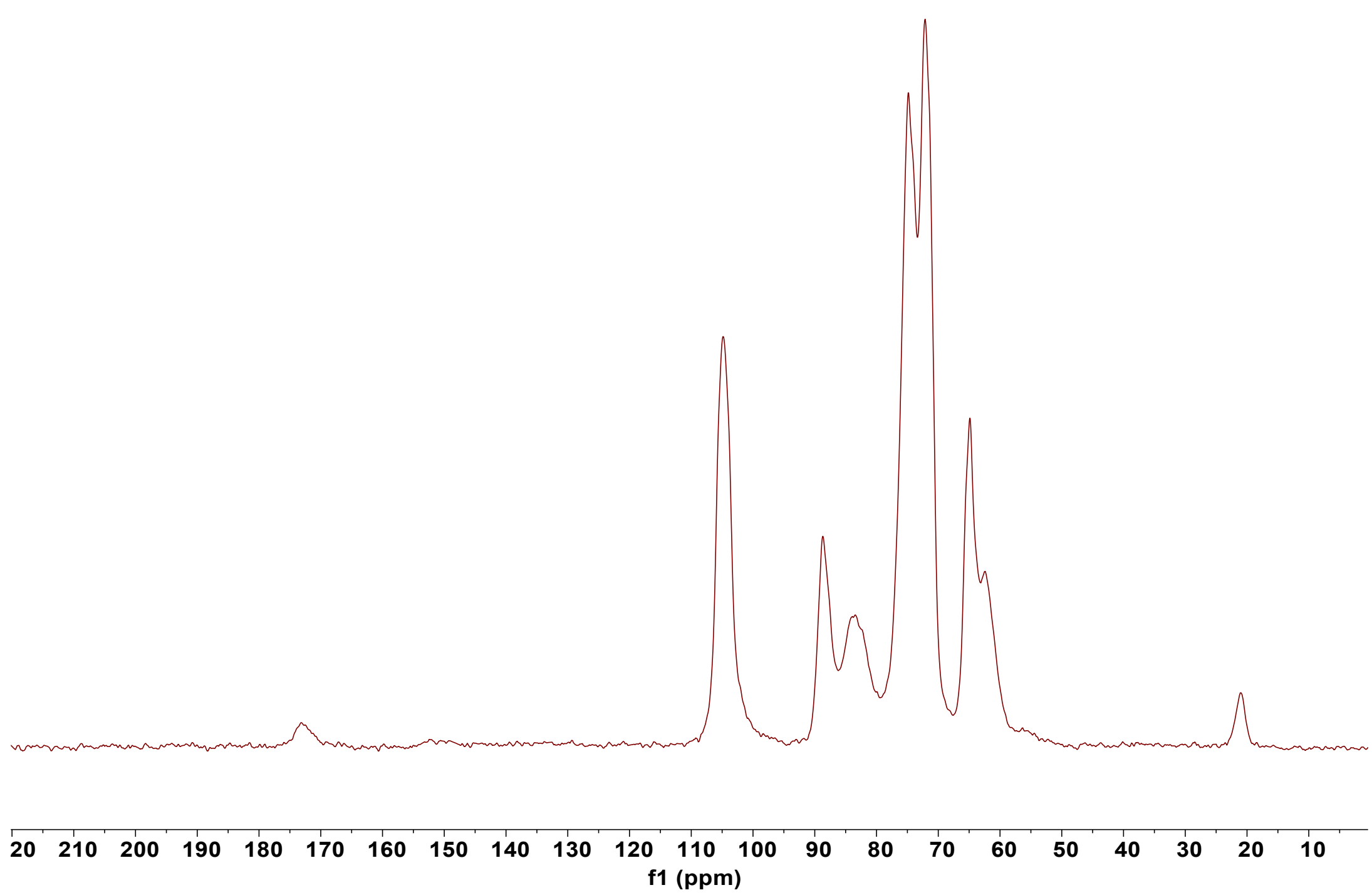

Figure S10. Solid state ^{13}C CP/MAS NMR spectrum (100 MHz) of CS-4.

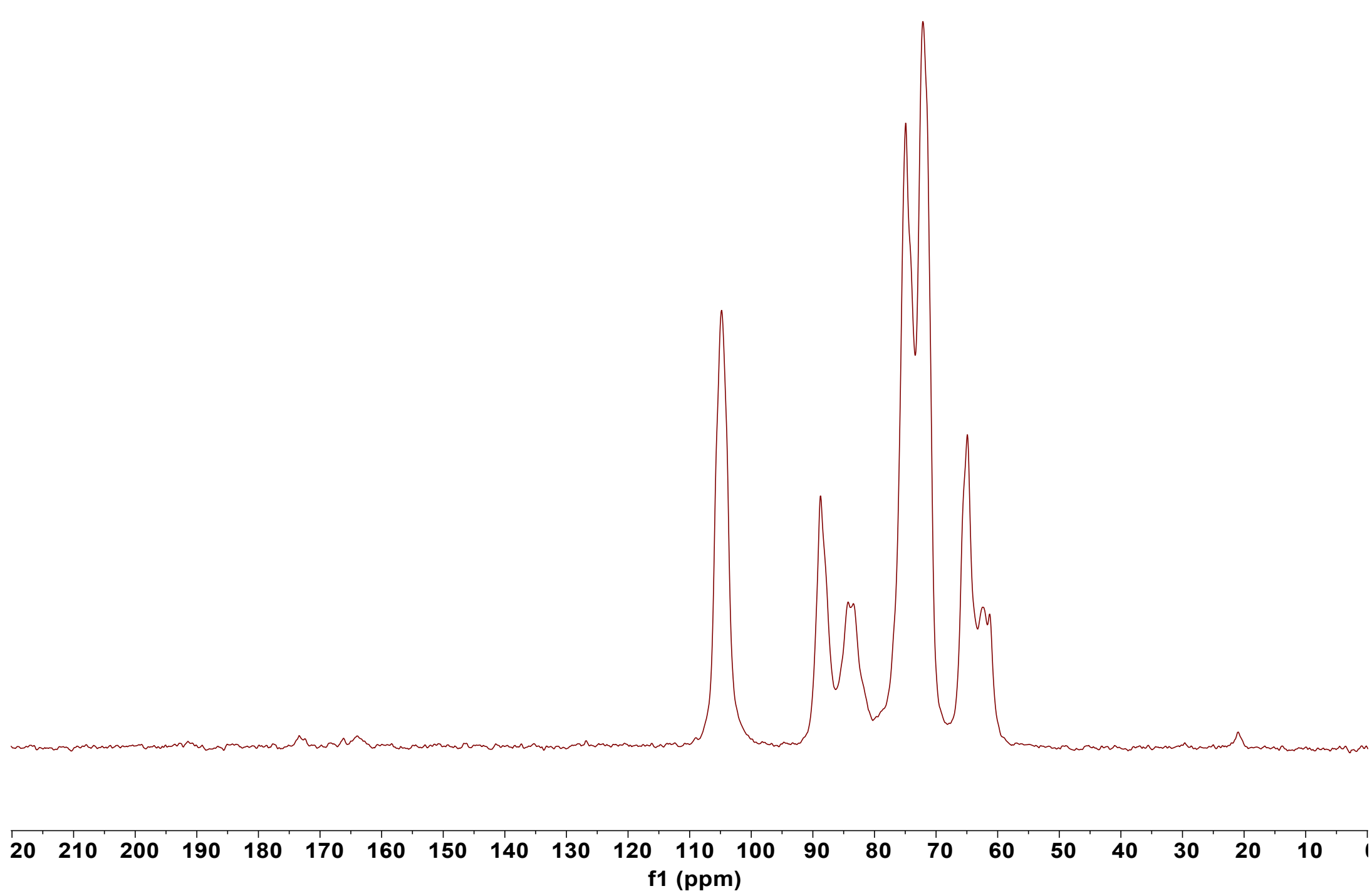

Figure S11. Solid state ^{13}C CP/MAS NMR spectrum (100 MHz) of CS-5.

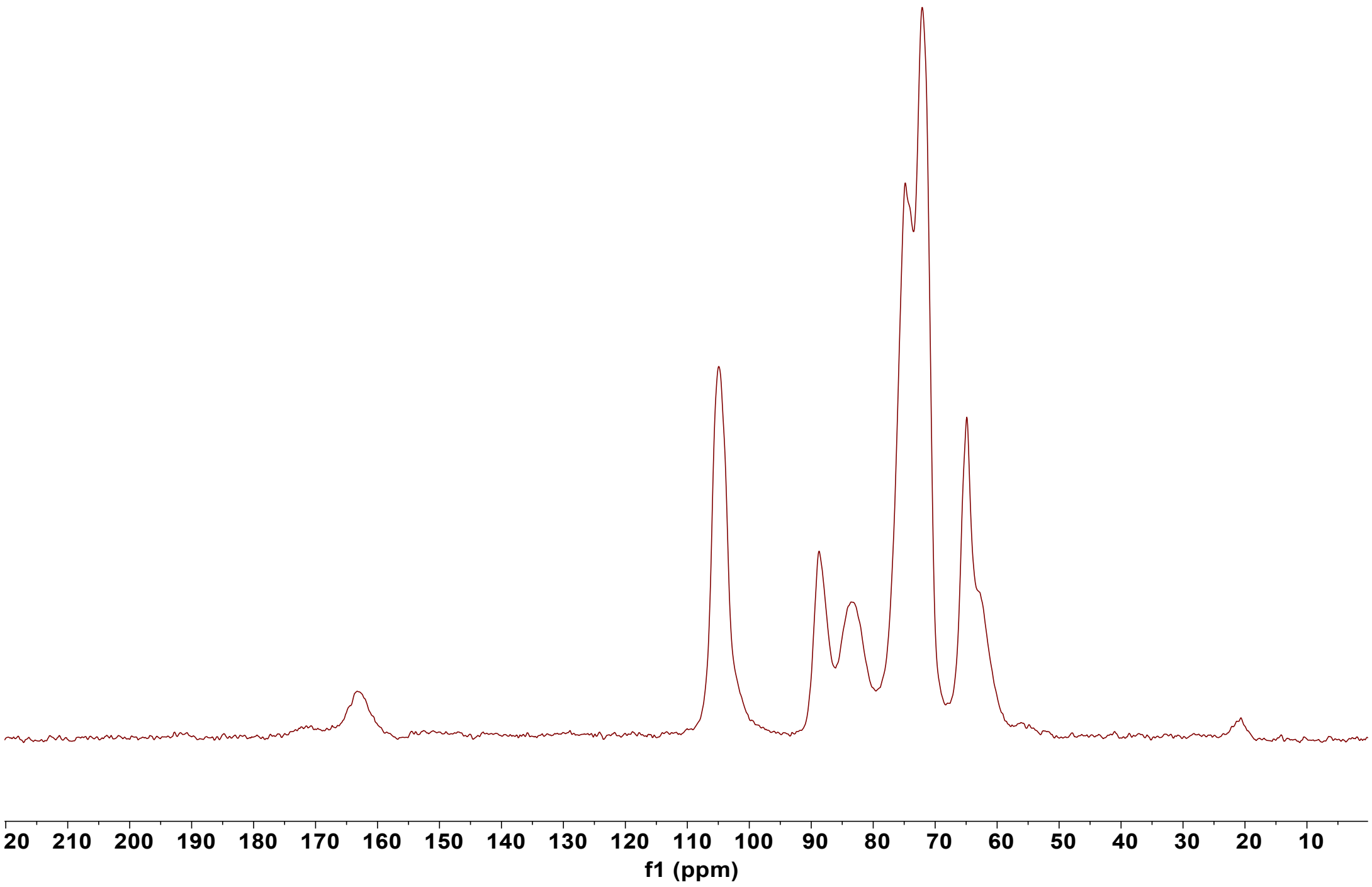

Figure S12. Solid state ^{13}C CP/MAS NMR spectrum (100 MHz) of CS-6.

1.5. Solution state NMR spectra of the cellulosic solids

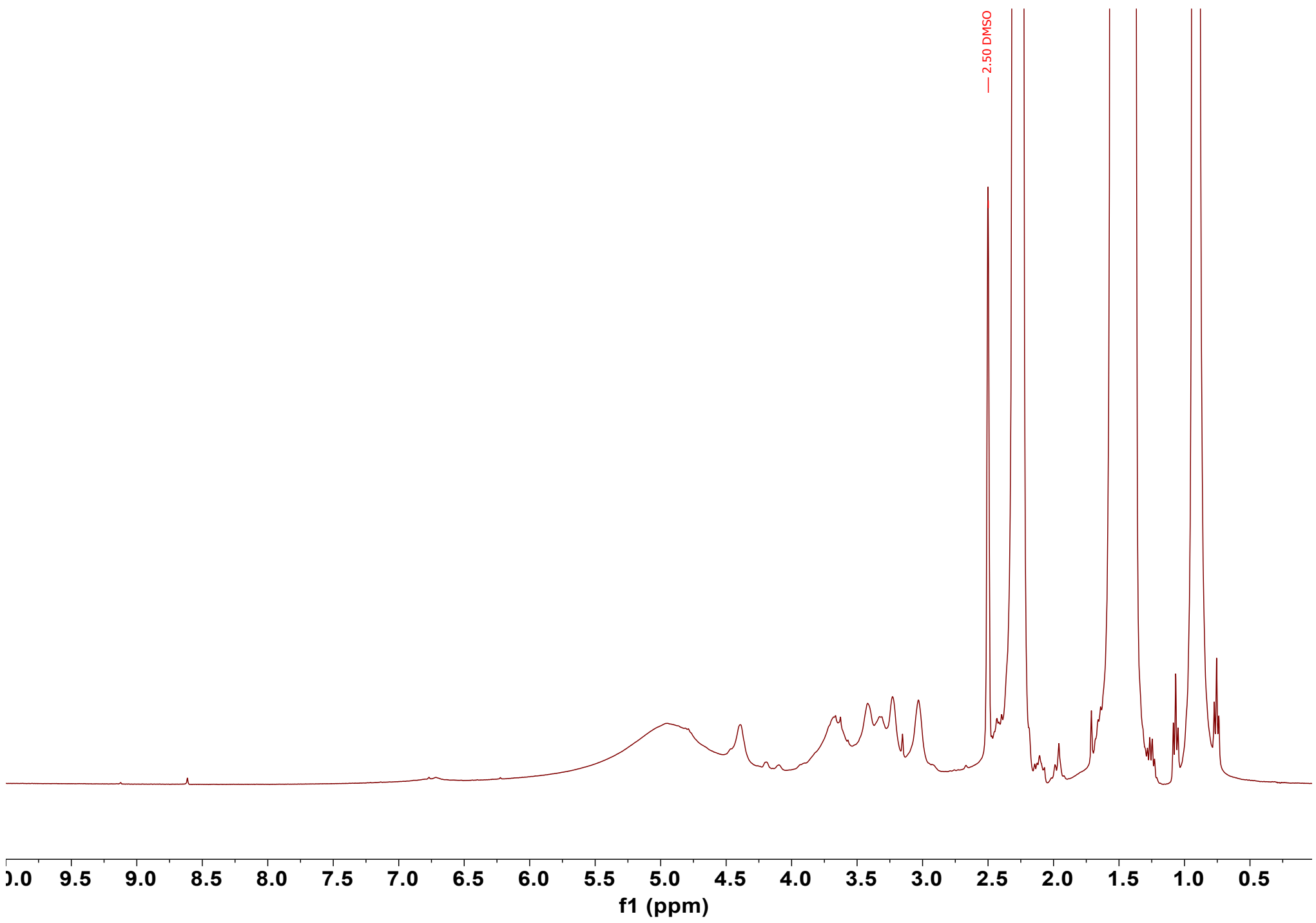

Figure S13. Quantitative ^{1}H NMR spectrum ([P_{4444}][OAc]:DMSO-d_6, 1:4 wt%; 400 MHz; 65°C) of the hydrothermally treated solids (2.5 wt%) used as the starting material for REx. Some fractions remained insoluble.

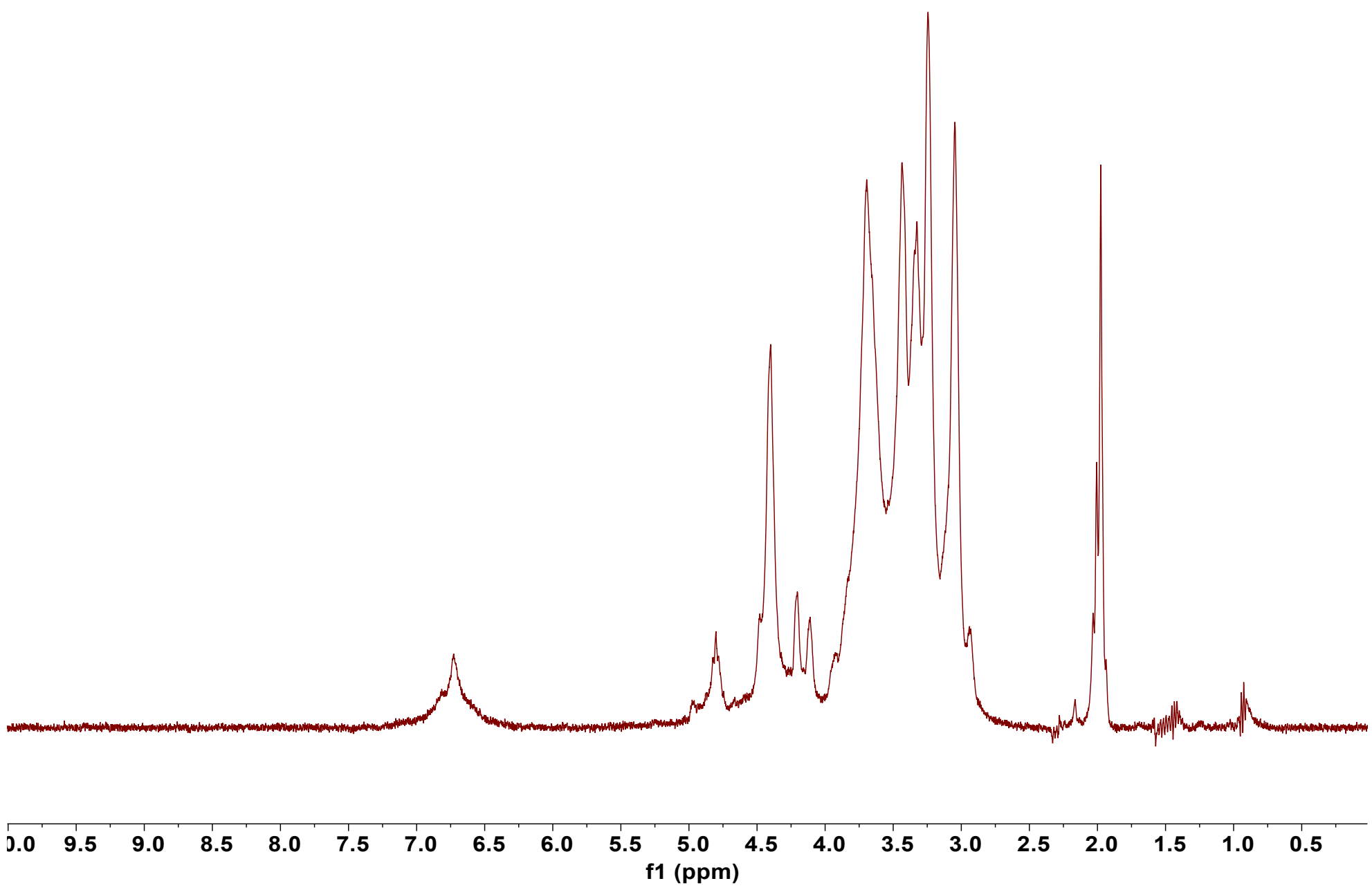

Figure S14. Diffusion edited ^{1}H NMR spectrum ([P_{4444}][OAc]:DMSO-d_6, 1:4 wt%; 400 MHz; 65°C) of the hydrothermally treated solids (2.5 wt%) used as the starting material for REx. Some fractions remained insoluble.

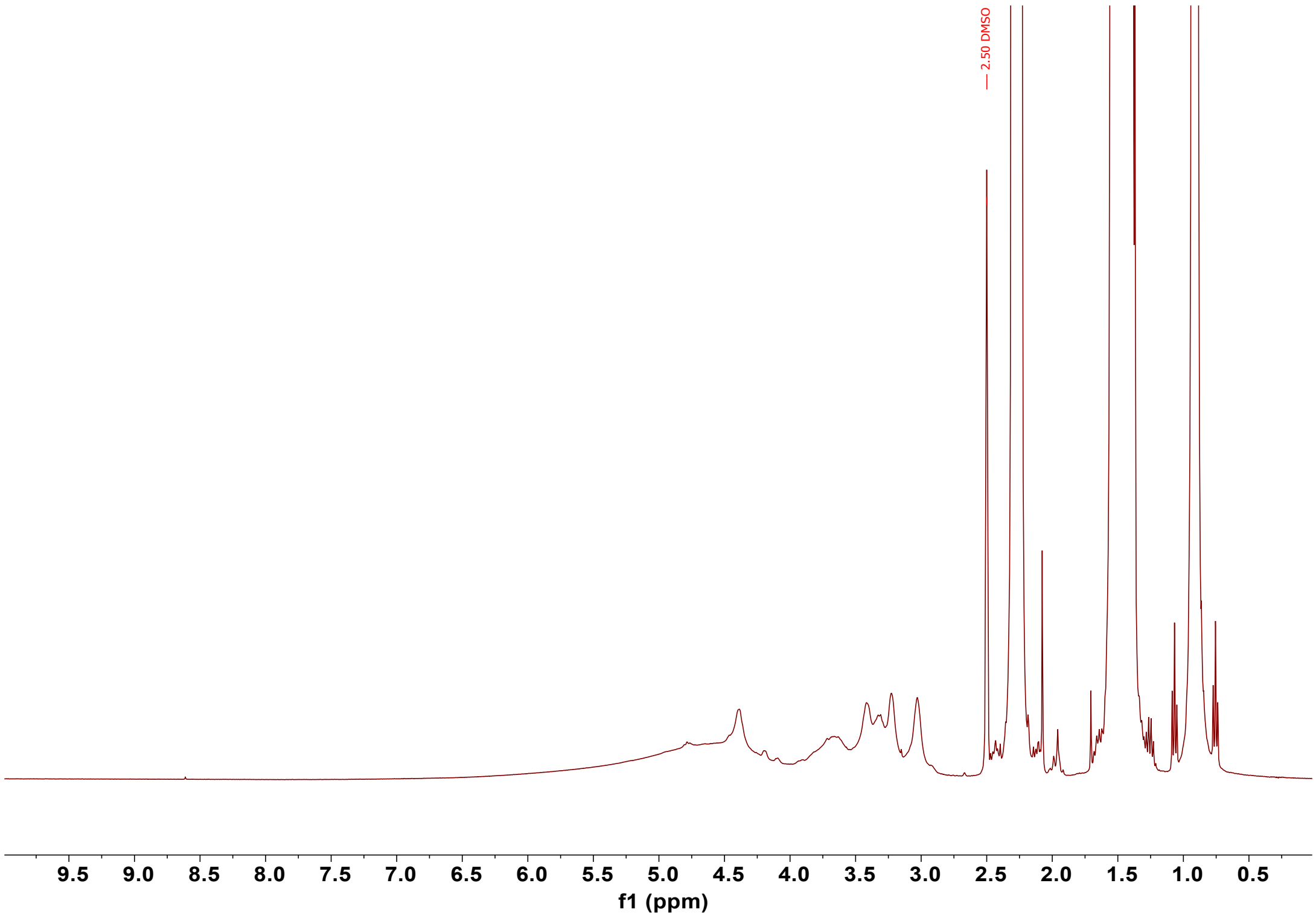

Figure S15. Quantitative ^{1}H NMR spectrum ($[P_{4444}][OAc]$:DMSO-d_6, 1:4 wt%; 400 MHz; 65°C) of the bleached hydrothermally treated solids (2.5 wt%).

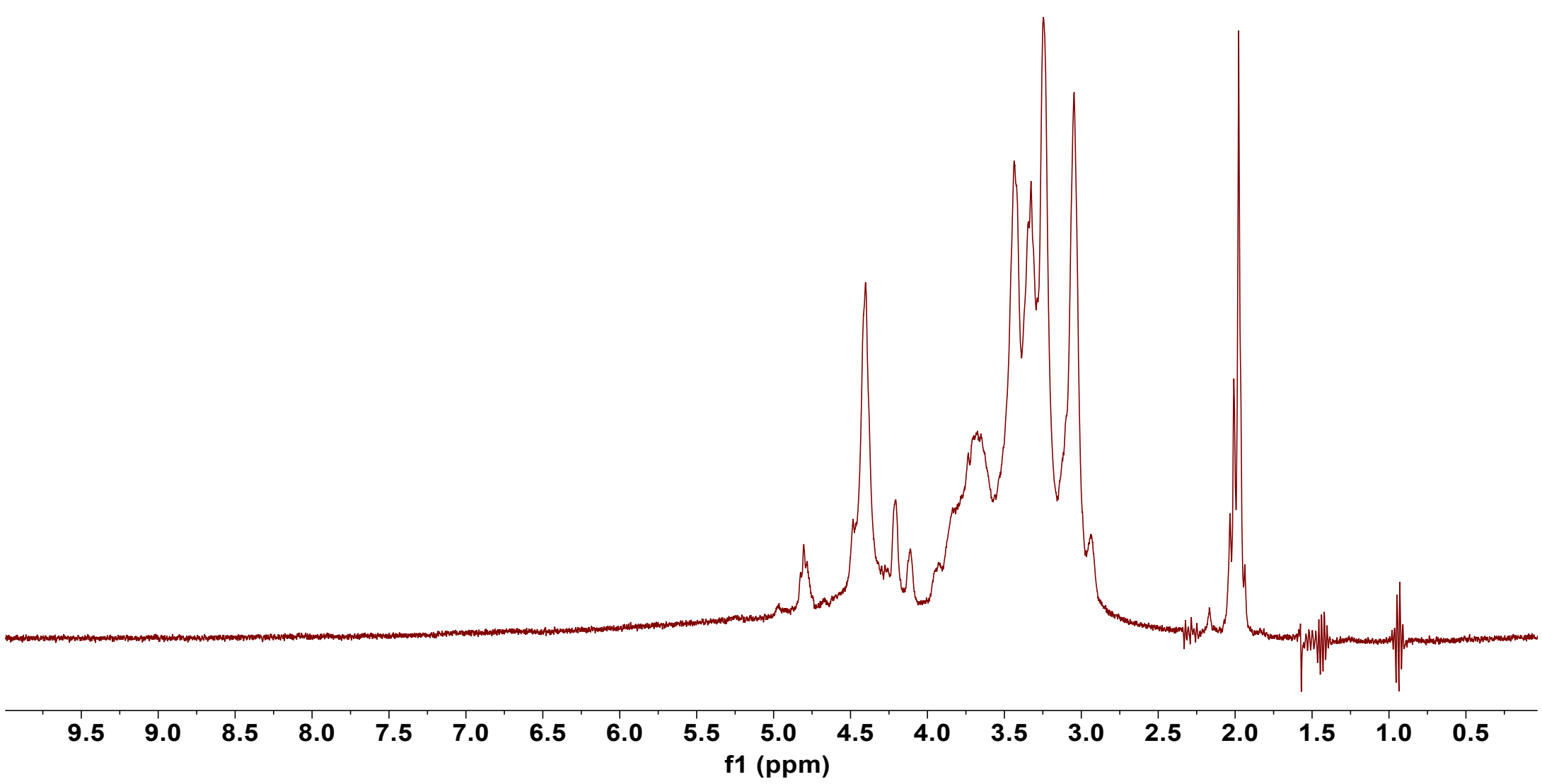

Figure S16. Diffusion edited ^{1}H NMR spectrum ($[P_{4444}][OAc]$:DMSO-d_6, 1:4 wt%; 400 MHz; 65°C) of the bleached hydrothermally treated solids (2.5 wt%).

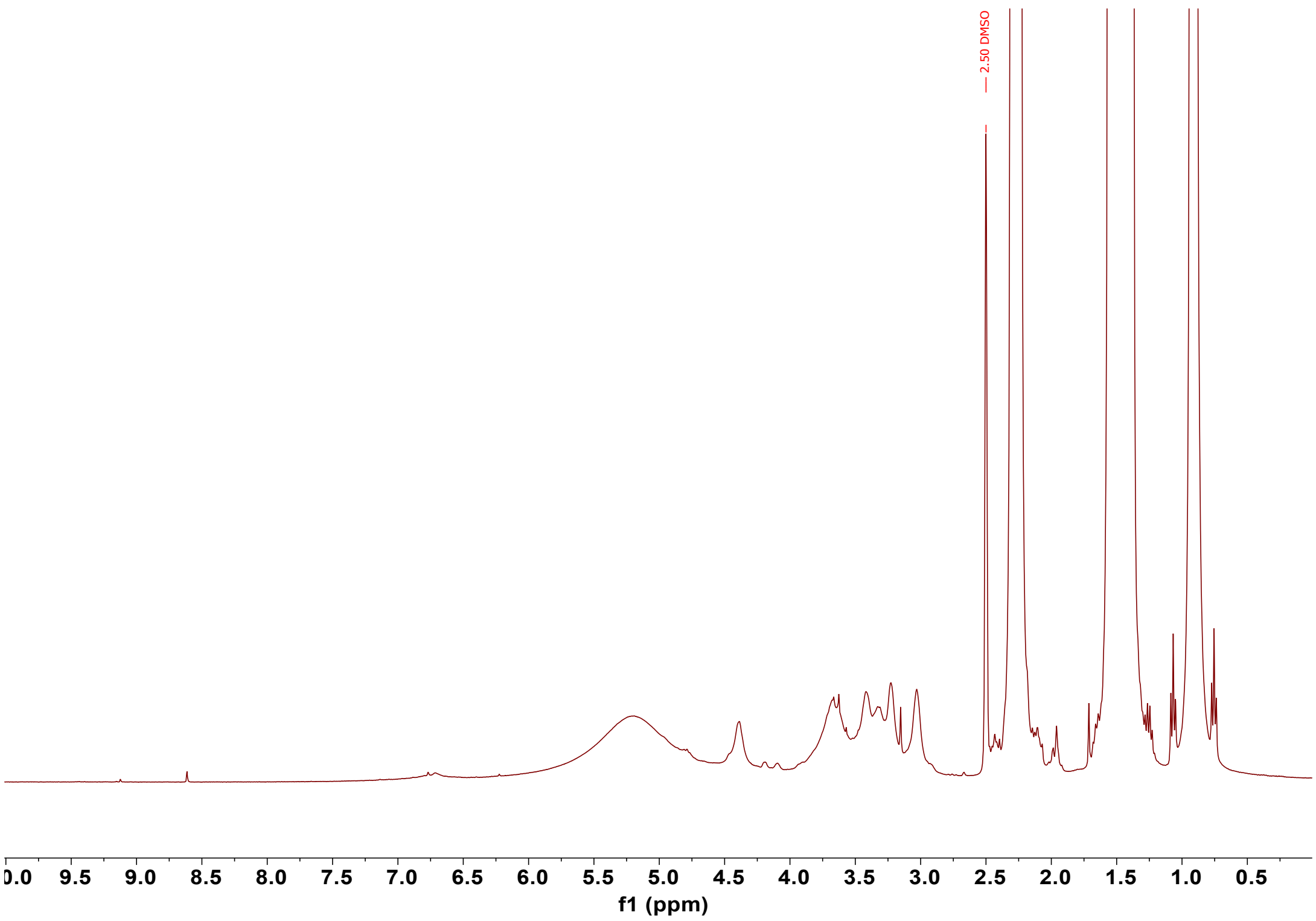

Figure S17. Quantitative ^{1}H NMR spectrum ([P_{4444}][OAc]:DMSO-d_6, 1:4 wt%; 400 MHz; 65°C) of CS-2 before bleaching (2.5 wt%). Some fractions remained insoluble.

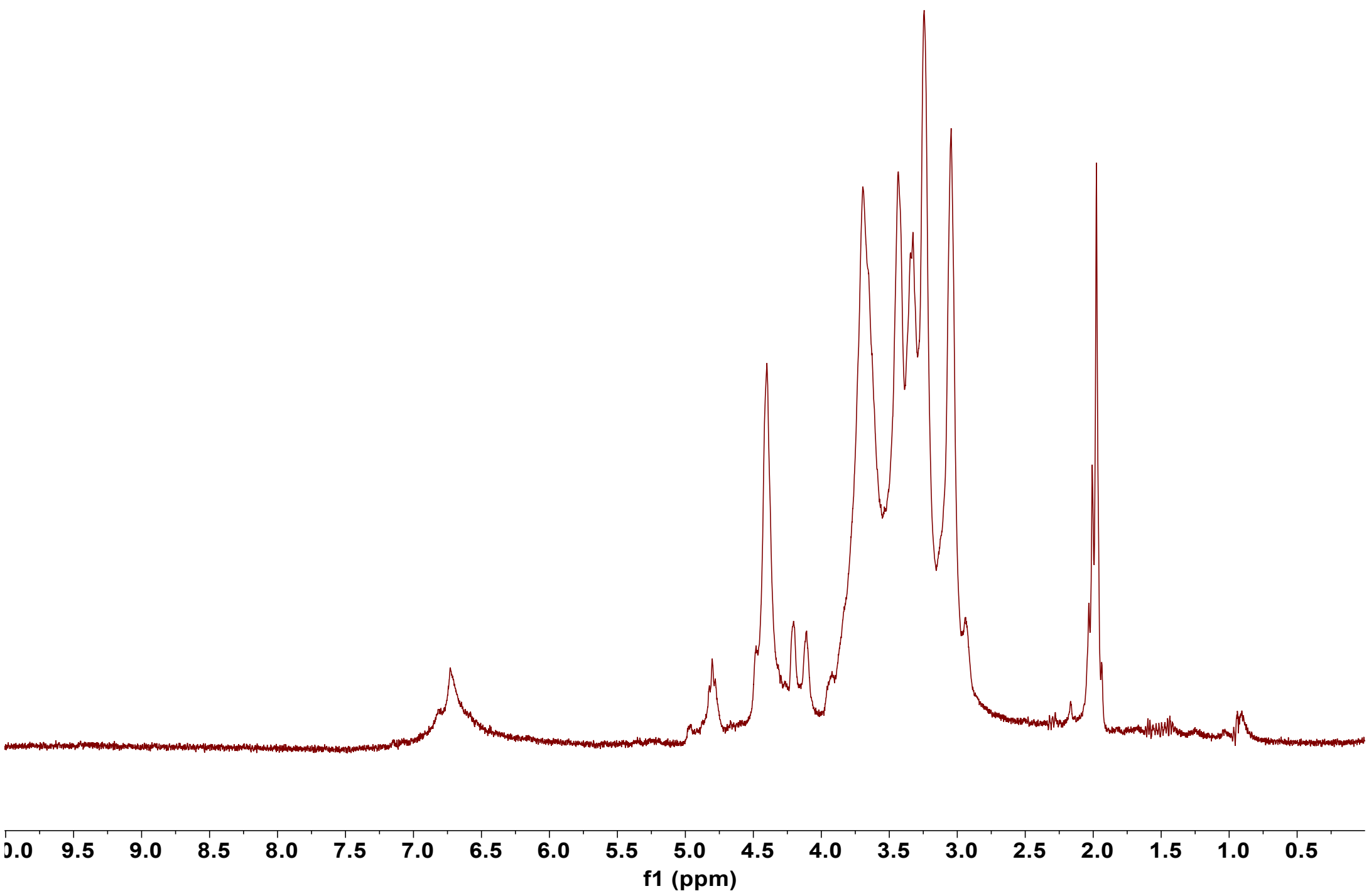

Figure S18. Diffusion edited ^{1}H NMR spectrum ([P_{4444}][OAc]:DMSO-d_6, 1:4 wt%; 400 MHz; 65°C) of CS-2 before bleaching (2.5 wt%). Some fractions remained insoluble.

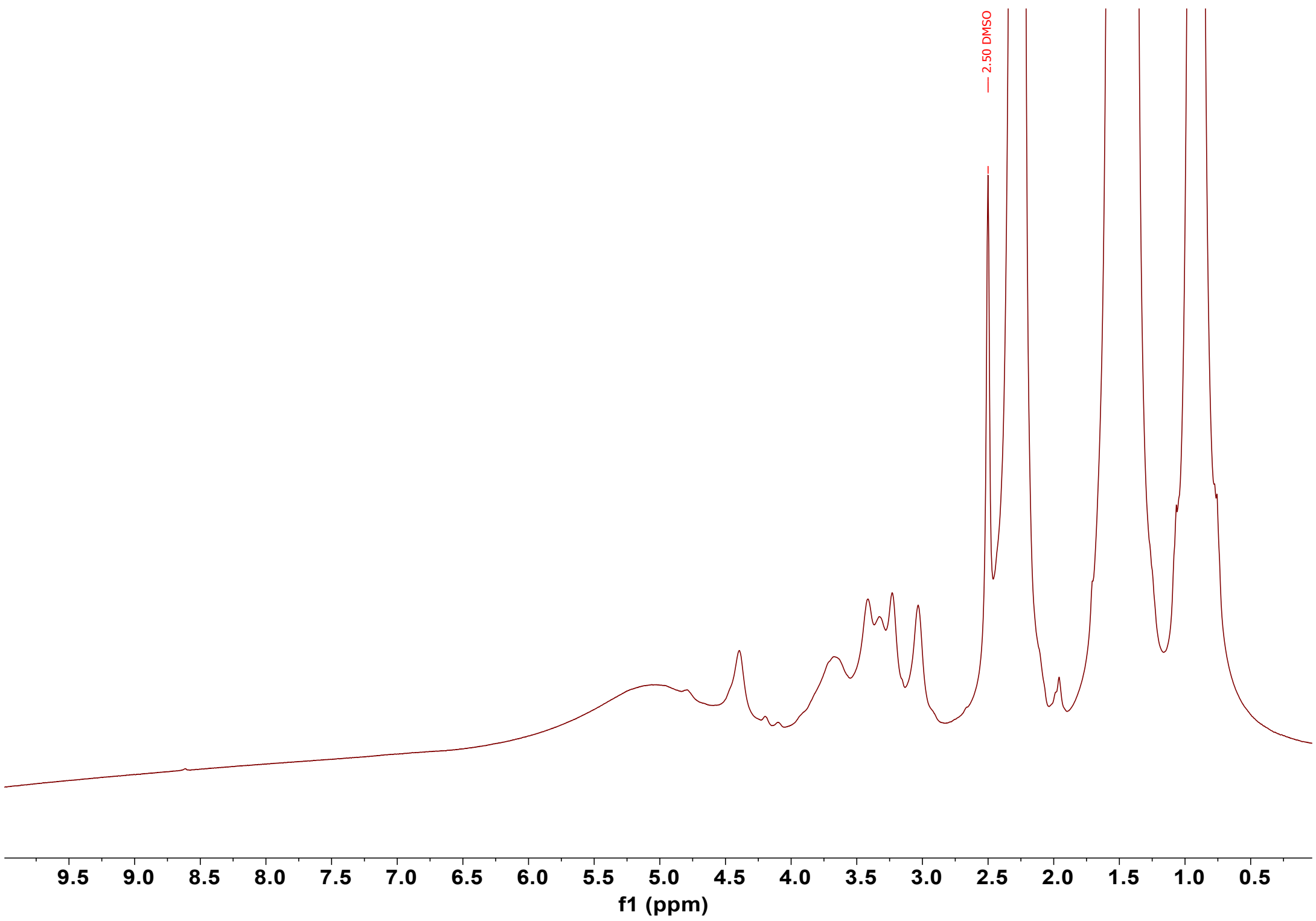

Figure S19. Quantitative ^{1}H NMR spectrum ($[P_{4444}][OAc]$:DMSO-d_6, 1:4 wt%; 400 MHz; 65°C) of CS-2 after bleaching (2.5 wt%).

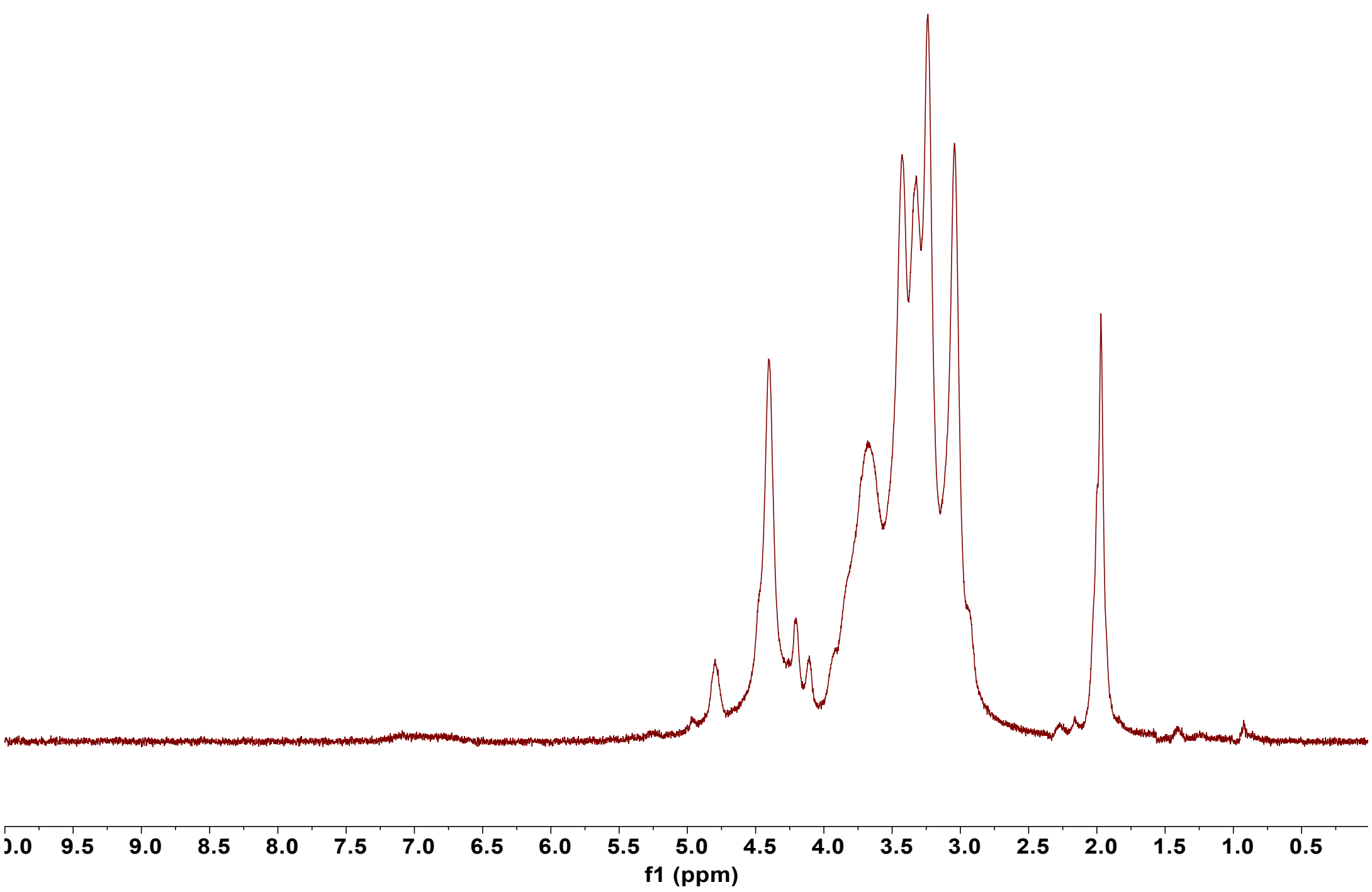

Figure S20. Diffusion edited ^{1}H NMR spectrum ($[P_{4444}][OAc]$:DMSO-d_6, 1:4 wt%; 400 MHz; 65°C) of CS-2 after bleaching (2.5 wt%).

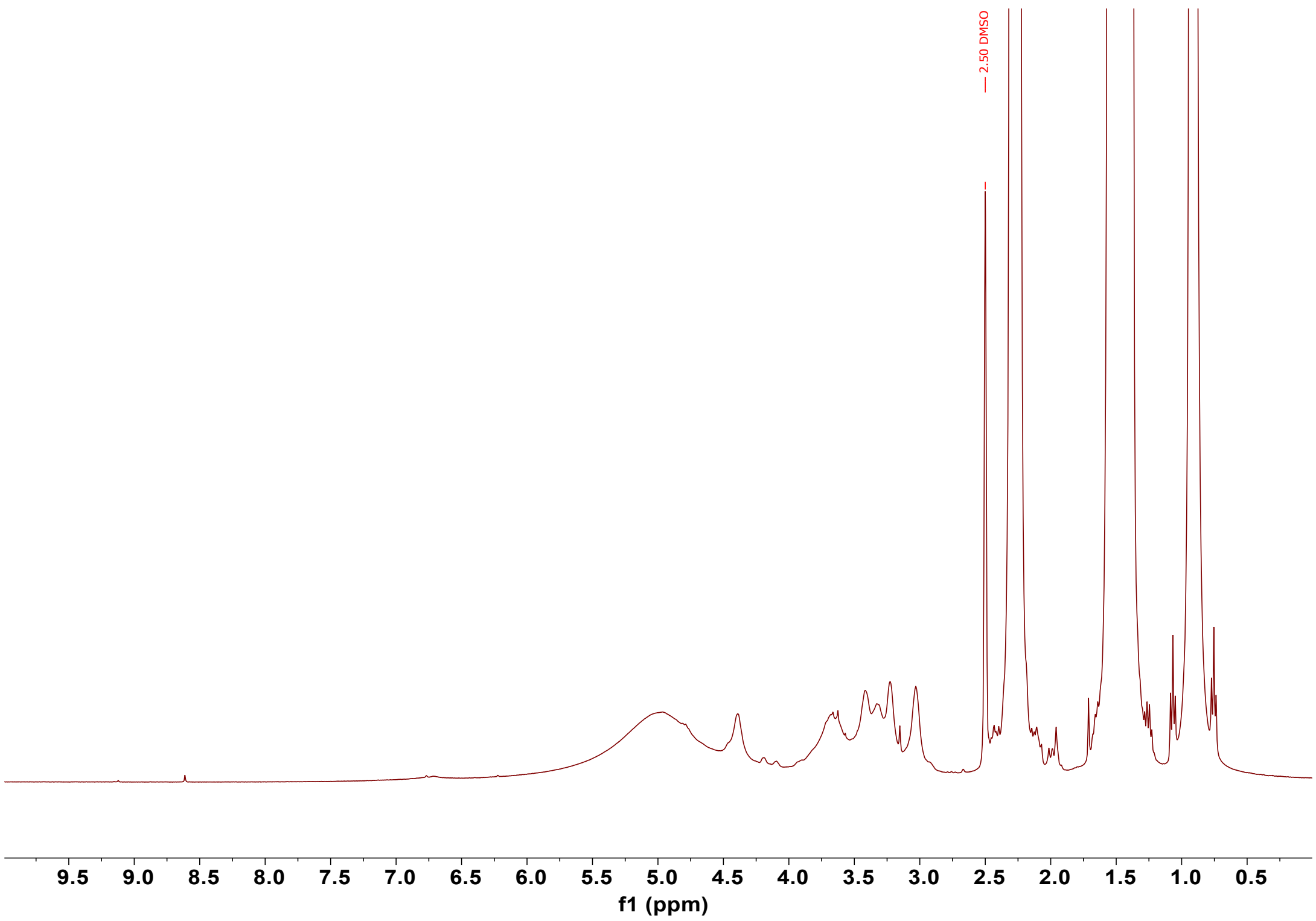

Figure S21. Quantitative ^{1}H NMR spectrum ([P_{4444}][OAc]:DMSO-*d*$_6$, 1:4 wt%; 400 MHz; 65°C) of CS-3 before bleaching (2.5 wt%). Some fractions remained insoluble.

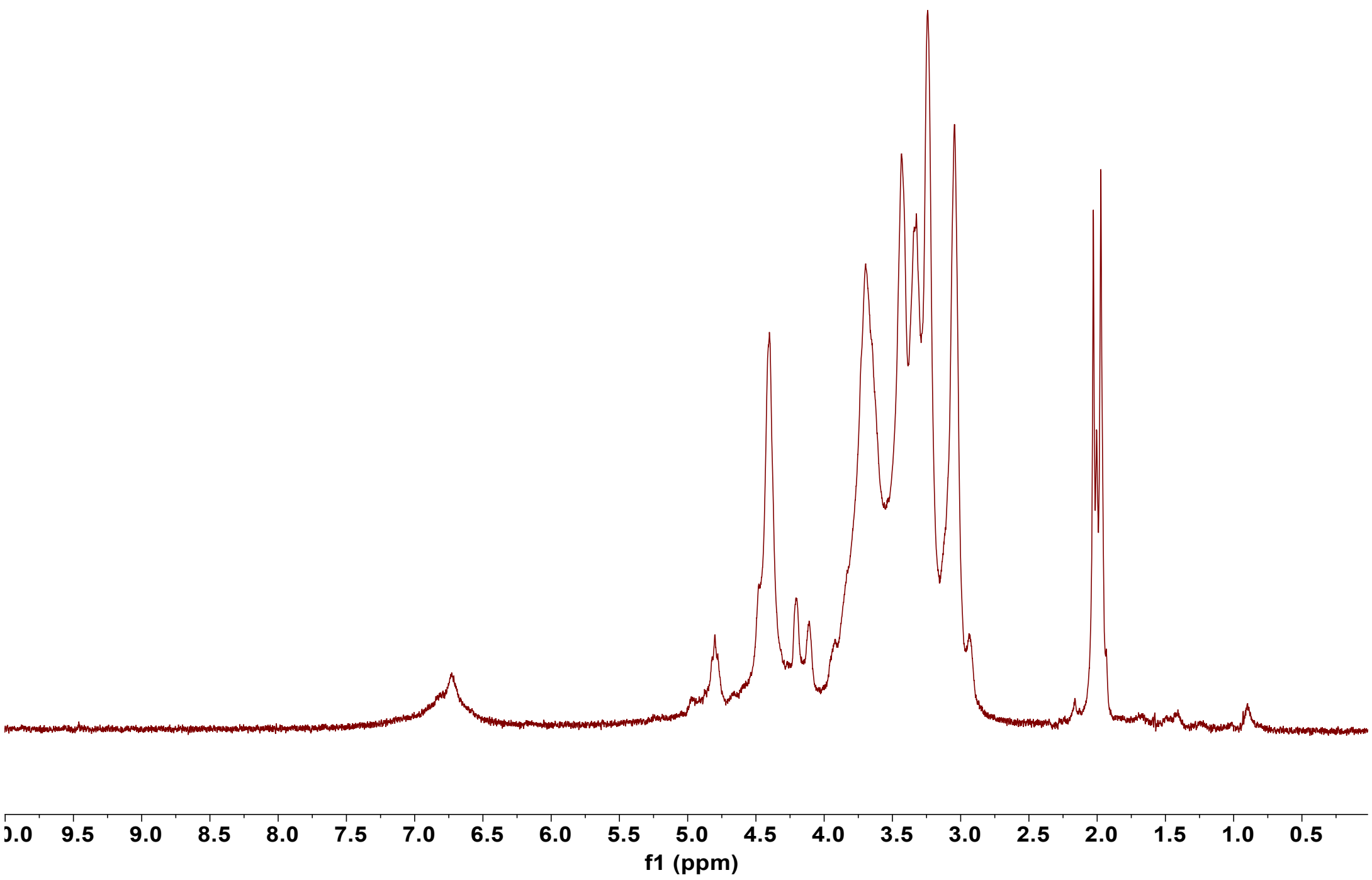

Figure S22. Diffusion edited ^{1}H NMR spectrum ([P_{4444}][OAc]:DMSO-*d*$_6$, 1:4 wt%; 400 MHz; 65°C) of CS-3 before bleaching (2.5 wt%). Some fractions remained insoluble.

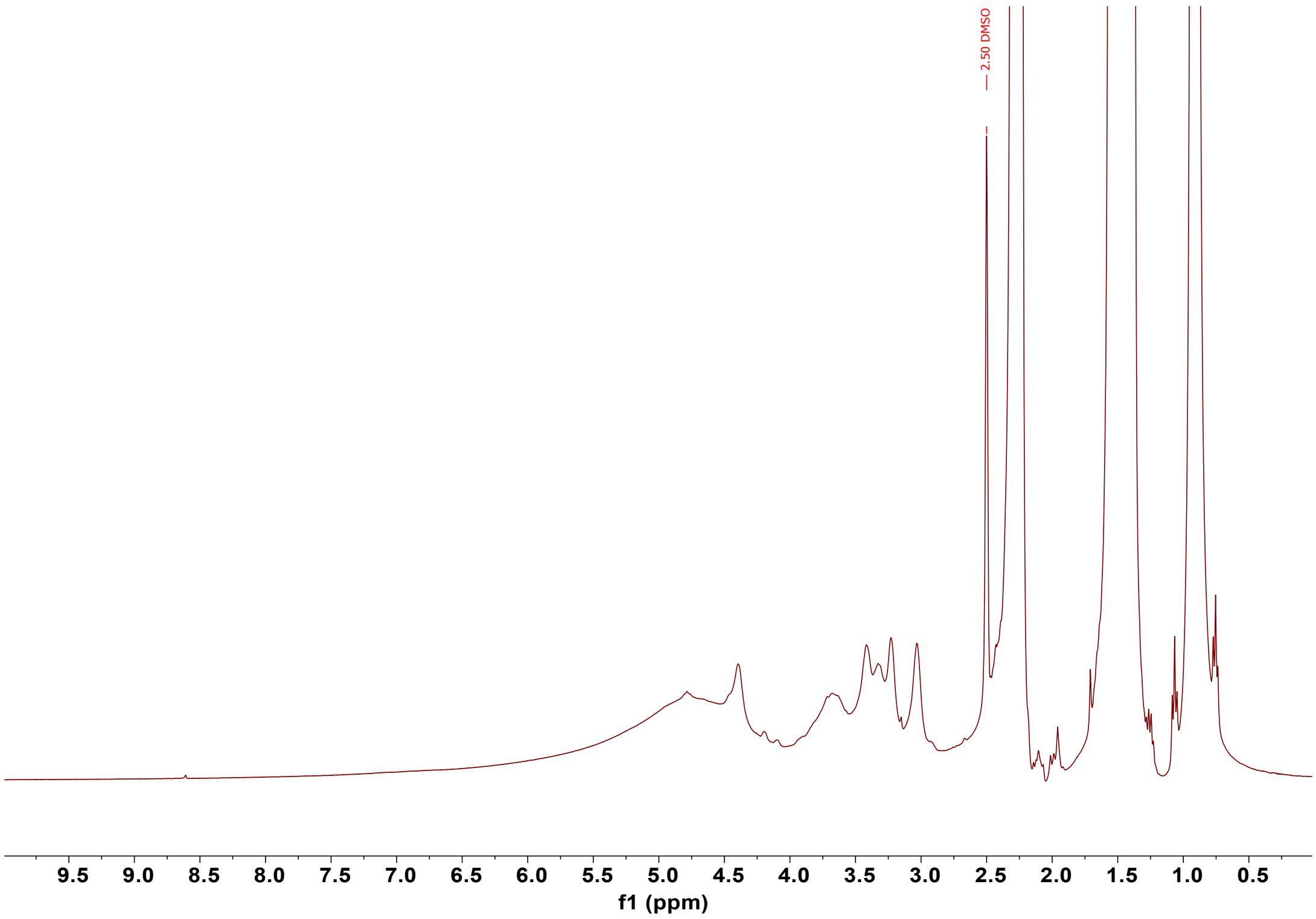

Figure S23. Quantitative ^{1}H NMR spectrum ([P_{4444}][OAc]:DMSO-d_6, 1:4 wt%; 400 MHz; 65°C) of CS-3 after bleaching (2.5 wt%).

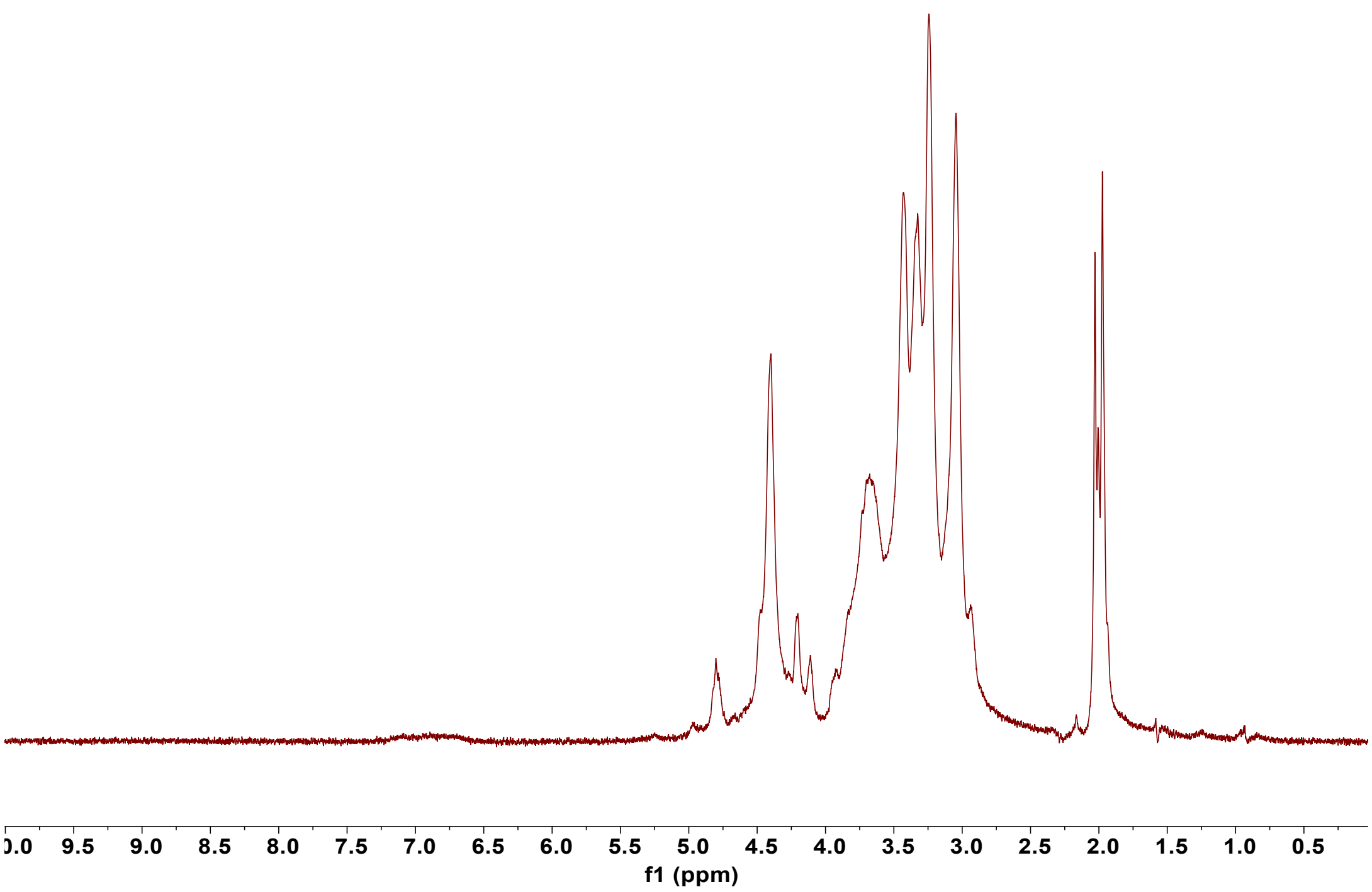

Figure S24. Diffusion edited ^{1}H NMR spectrum ([P_{4444}][OAc]:DMSO-d_6, 1:4 wt%; 400 MHz; 65°C) of CS-3 after bleaching (2.5 wt%).

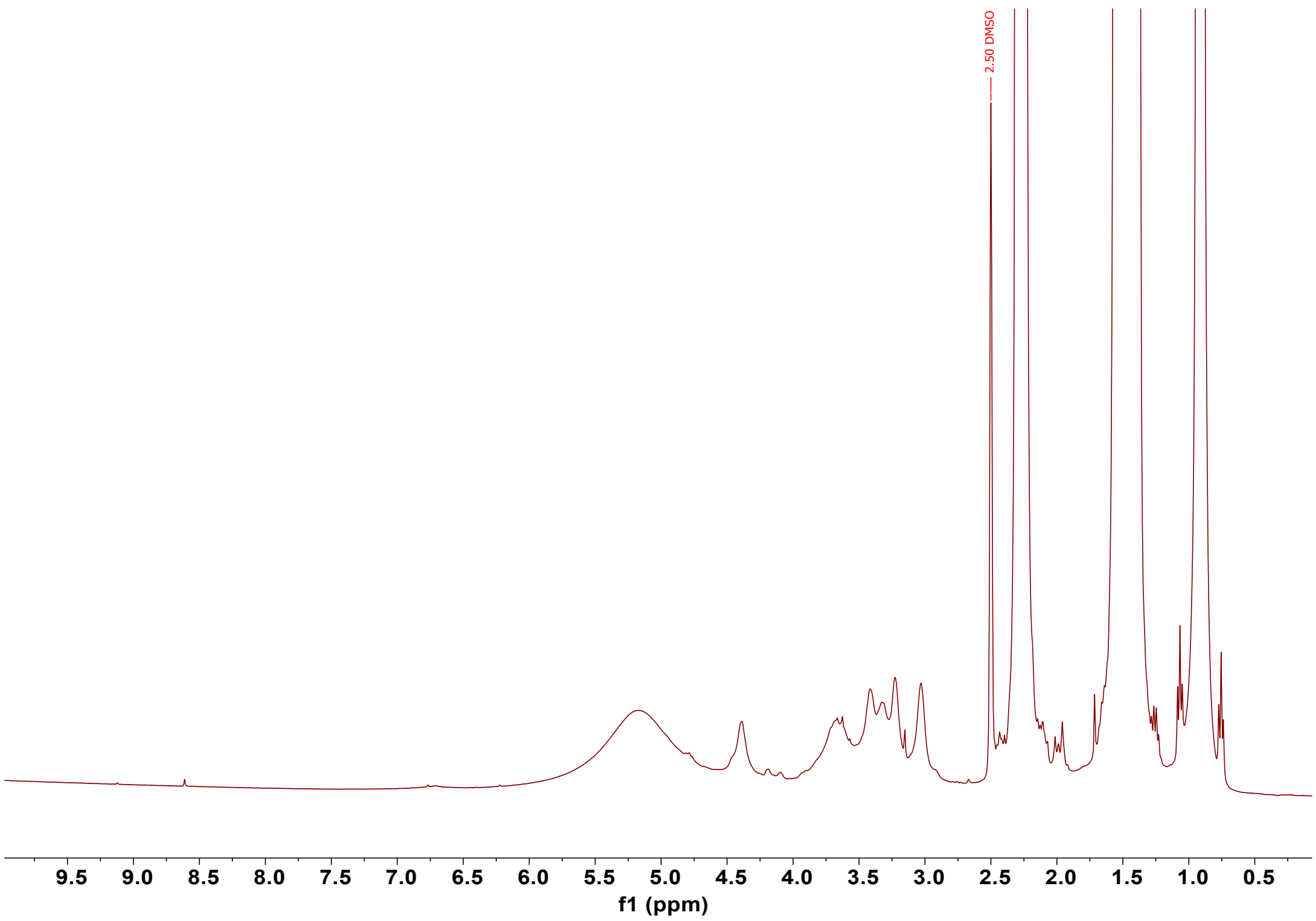

Figure S25. Quantitative ^{1}H NMR spectrum ($[P_{4444}][OAc]$:DMSO-d_6, 1:4 wt%; 400 MHz; 65°C) of CS-4 before bleaching (2.5 wt%). Some fractions remained insoluble.

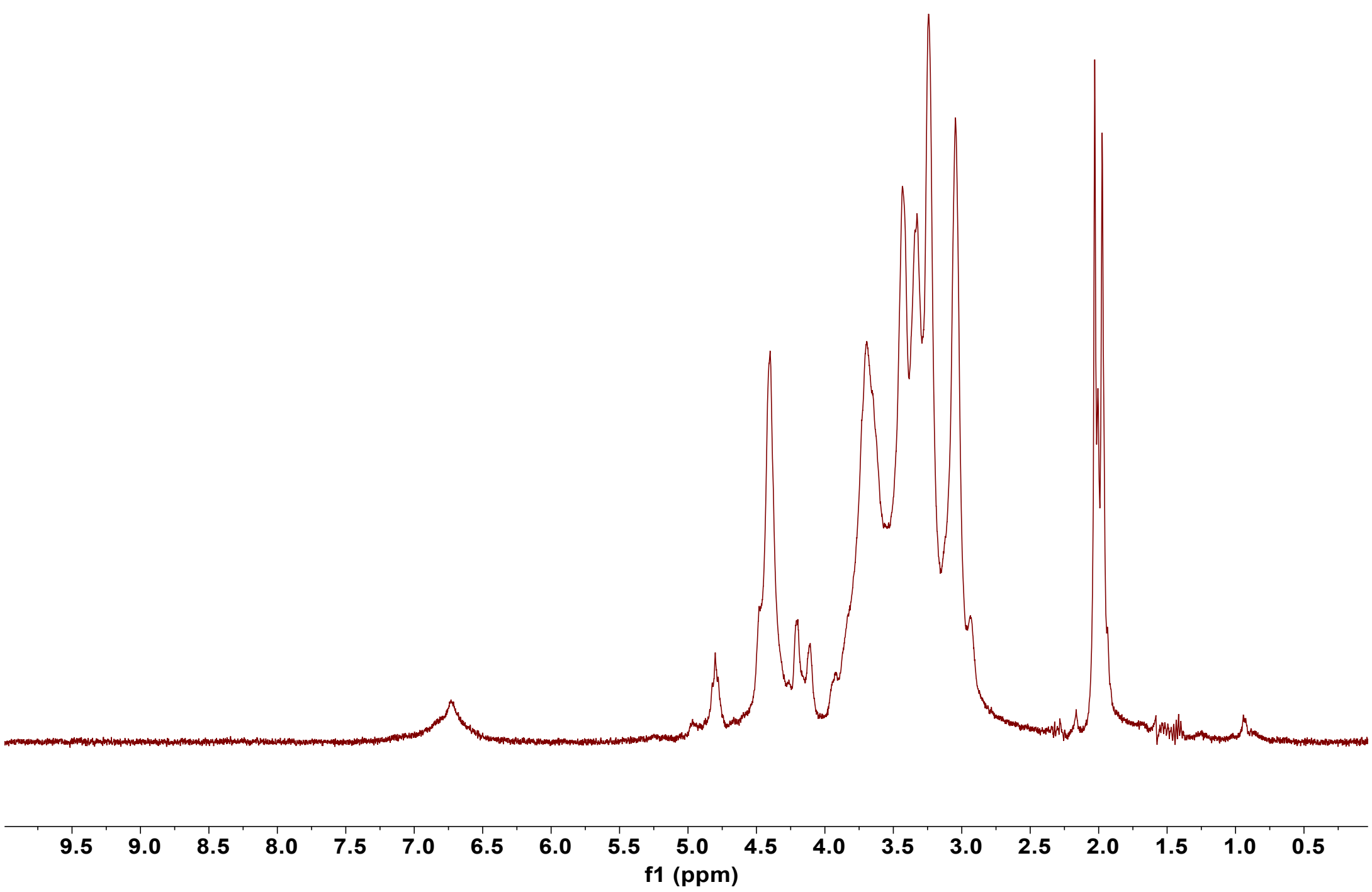

Figure S26. Diffusion edited ^{1}H NMR spectrum ($[P_{4444}][OAc]$:DMSO-d_6, 1:4 wt%; 400 MHz; 65°C) of CS-4 before bleaching (2.5 wt%). Some fractions remained insoluble.

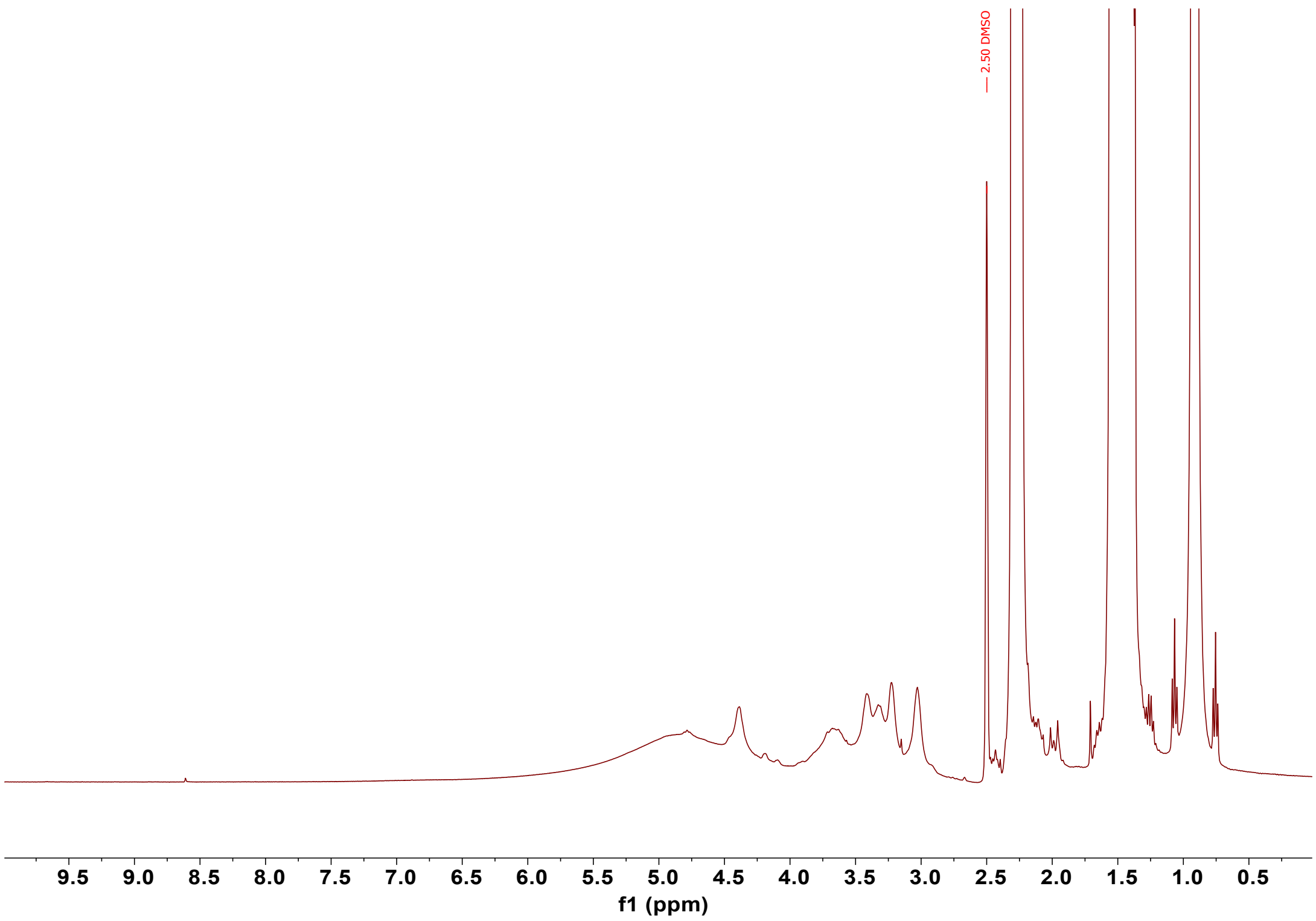

Figure S27. Quantitative ^{1}H NMR spectrum ([P_{4444}][OAc]:DMSO-d_6, 1:4 wt%; 400 MHz; 65°C) of CS-4 after bleaching (2.5 wt%).

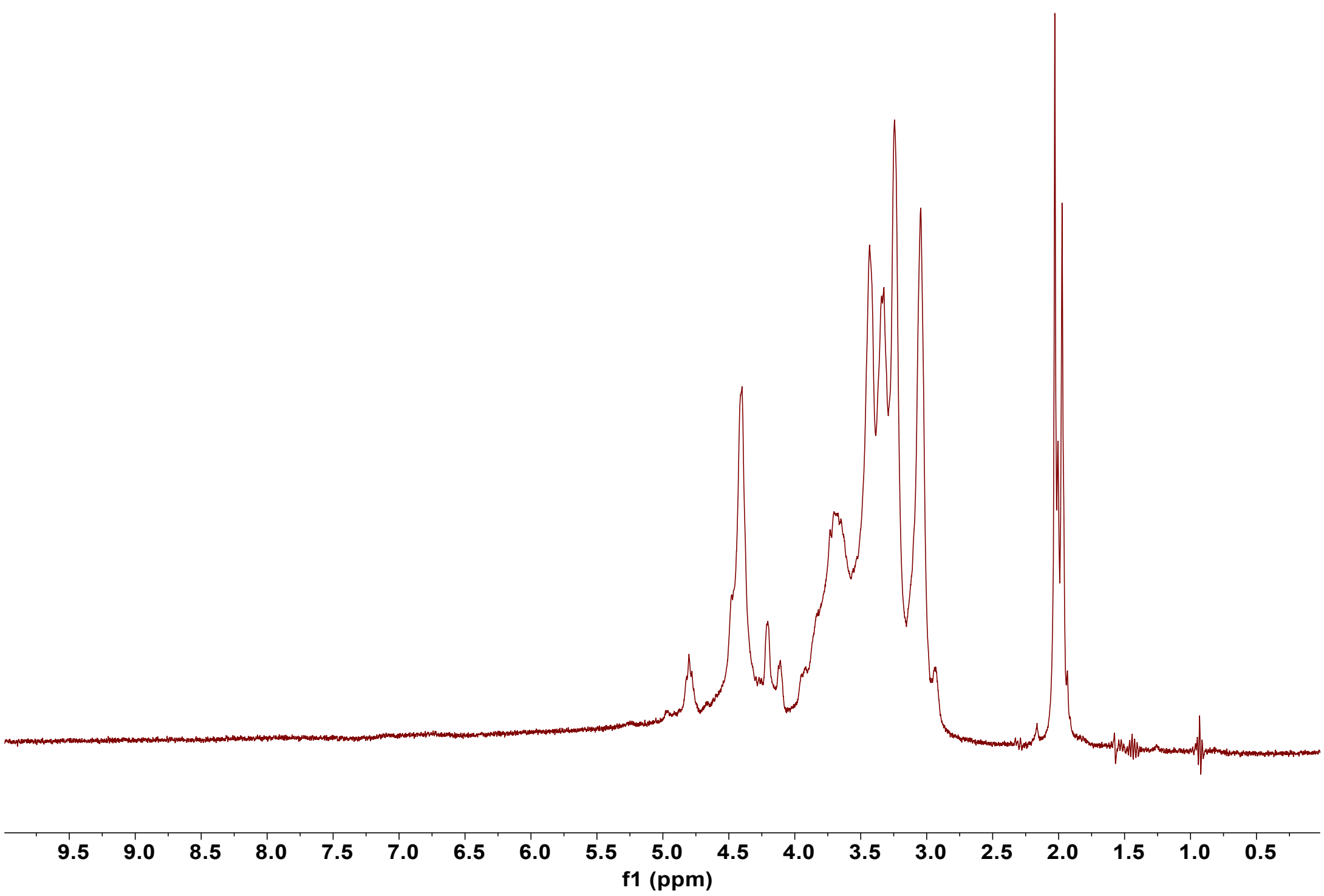

Figure S28. Diffusion edited ^{1}H NMR spectrum ([P_{4444}][OAc]:DMSO-d_6, 1:4 wt%; 400 MHz; 65°C) CS-4 after bleaching (2.5 wt%).

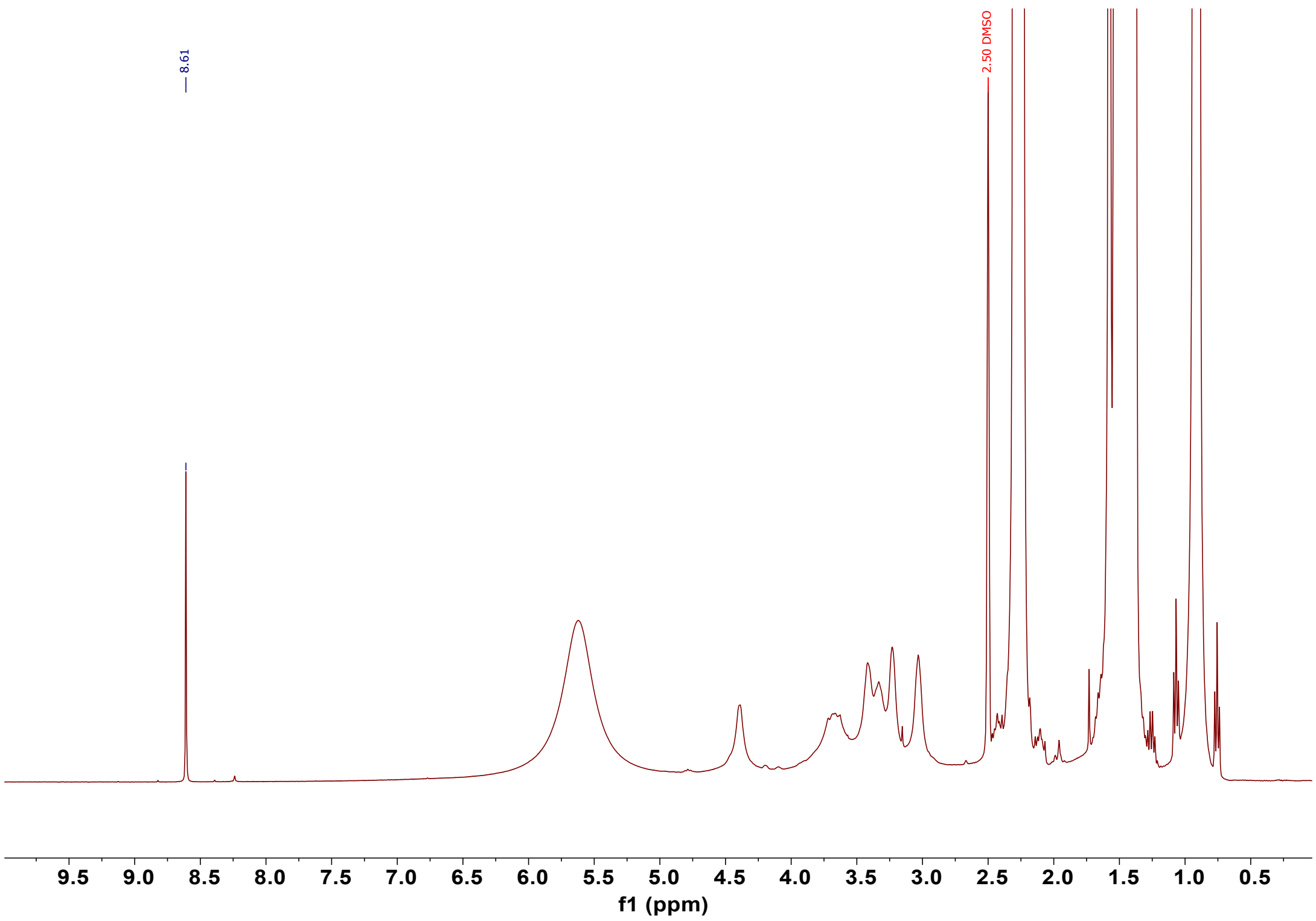

Figure S29. Quantitative ^{1}H NMR spectrum ($[P_{4444}][OAc]$:DMSO-d_6, 1:4 wt%; 400 MHz; 65°C) of CS-5 before bleaching (2.5 wt%). Some fractions remained insoluble.

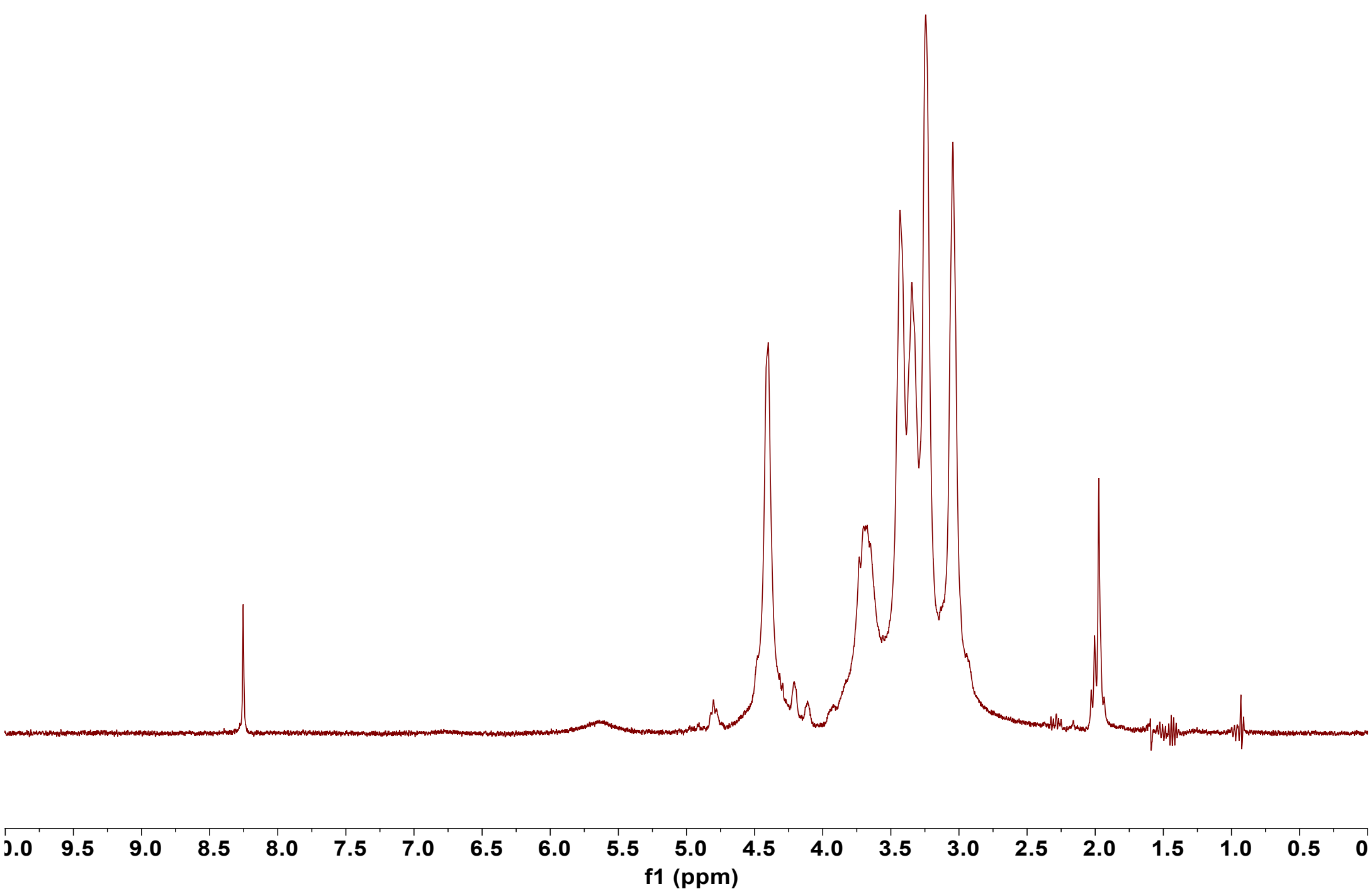

Figure S30. Diffusion edited ^{1}H NMR spectrum ($[P_{4444}][OAc]$:DMSO-d_6, 1:4 wt%; 400 MHz; 65°C) of CS-5 before bleaching (2.5 wt%). Some fractions remained insoluble.

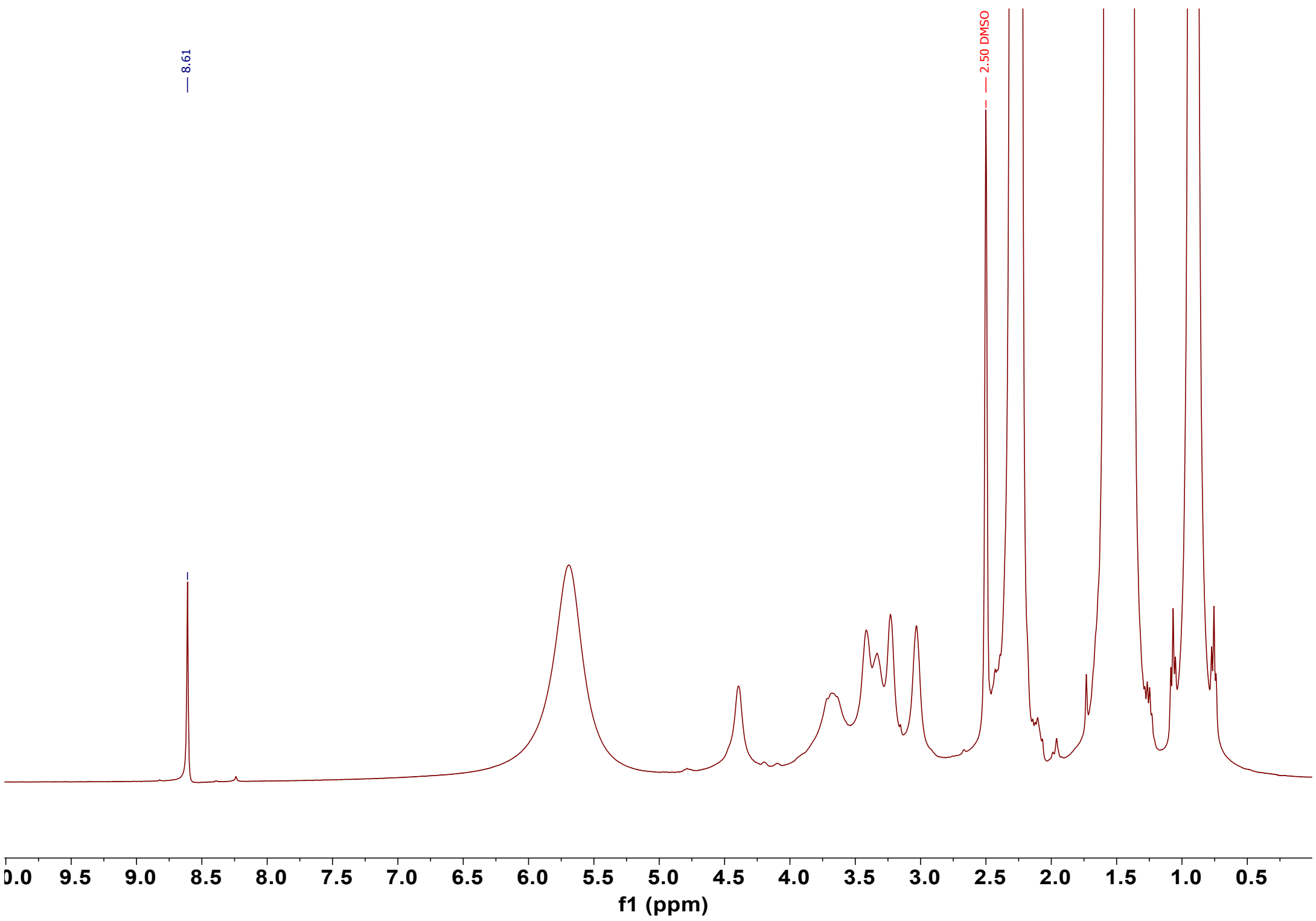

Figure S31. Quantitative ^{1}H NMR spectrum ([P_{4444}][OAc]:DMSO-d_6, 1:4 wt%; 400 MHz; 65°C) of CS-5 after bleaching (2.5 wt%).

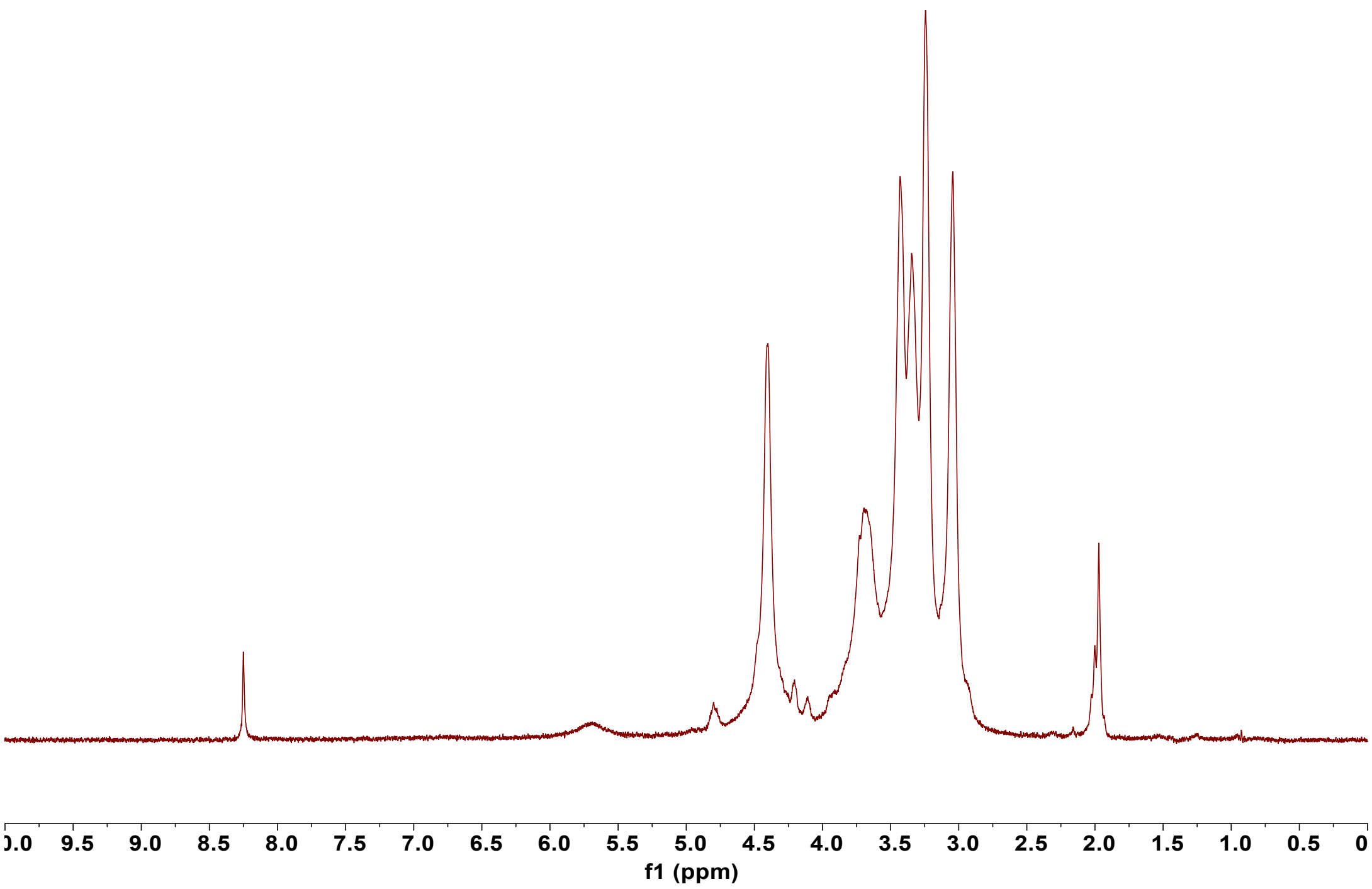

Figure S32. Diffusion edited ^{1}H NMR spectrum ([P_{4444}][OAc]:DMSO-d_6, 1:4 wt%; 400 MHz; 65°C) CS-5 after bleaching (2.5 wt%).

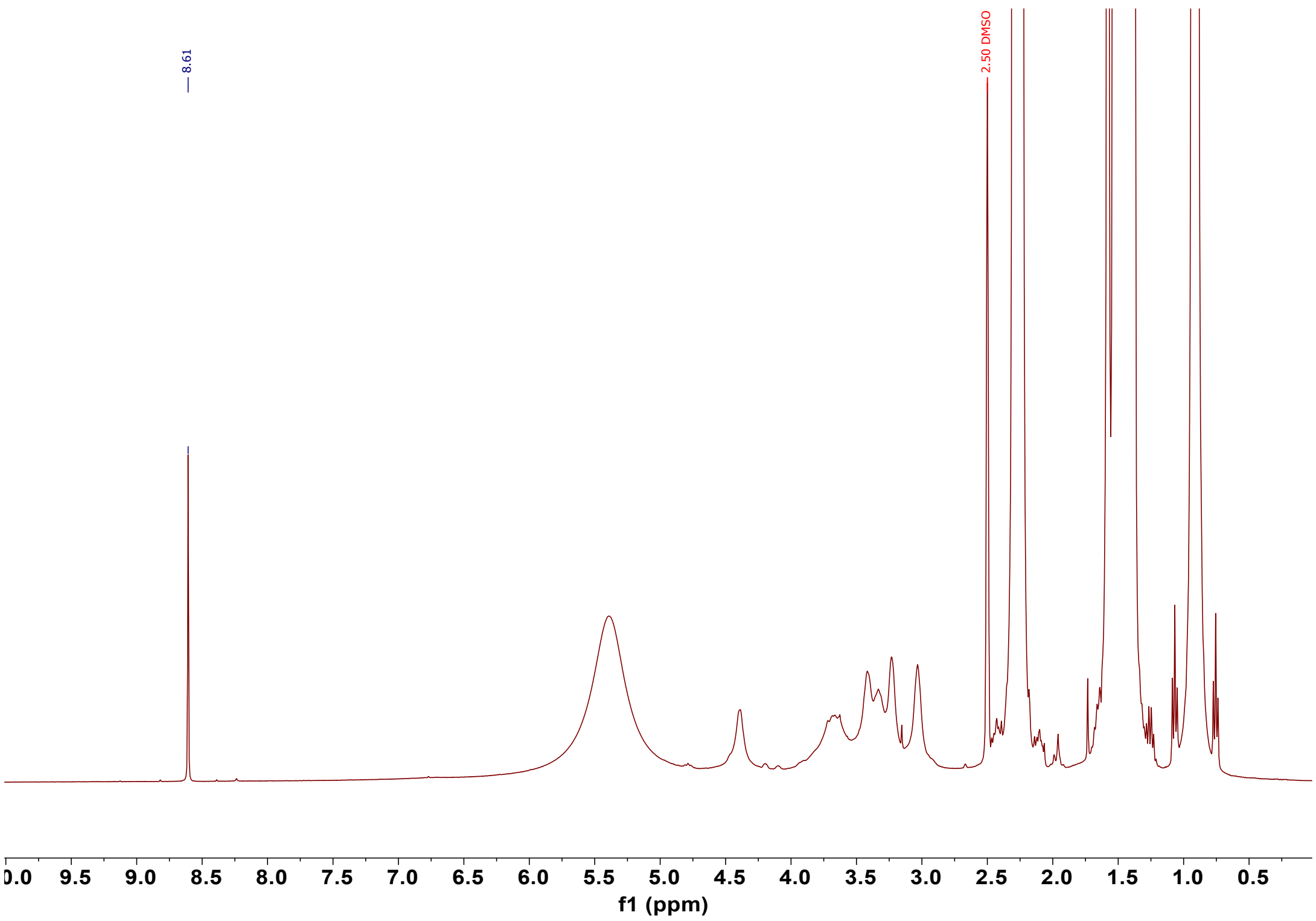

Figure S33. Quantitative ^{1}H NMR spectrum ([P_{4444}][OAc]:DMSO-d_6, 1:4 wt%; 400 MHz; 65°C) CS-6 before bleaching (2.5 wt%). Some fractions remained insoluble.

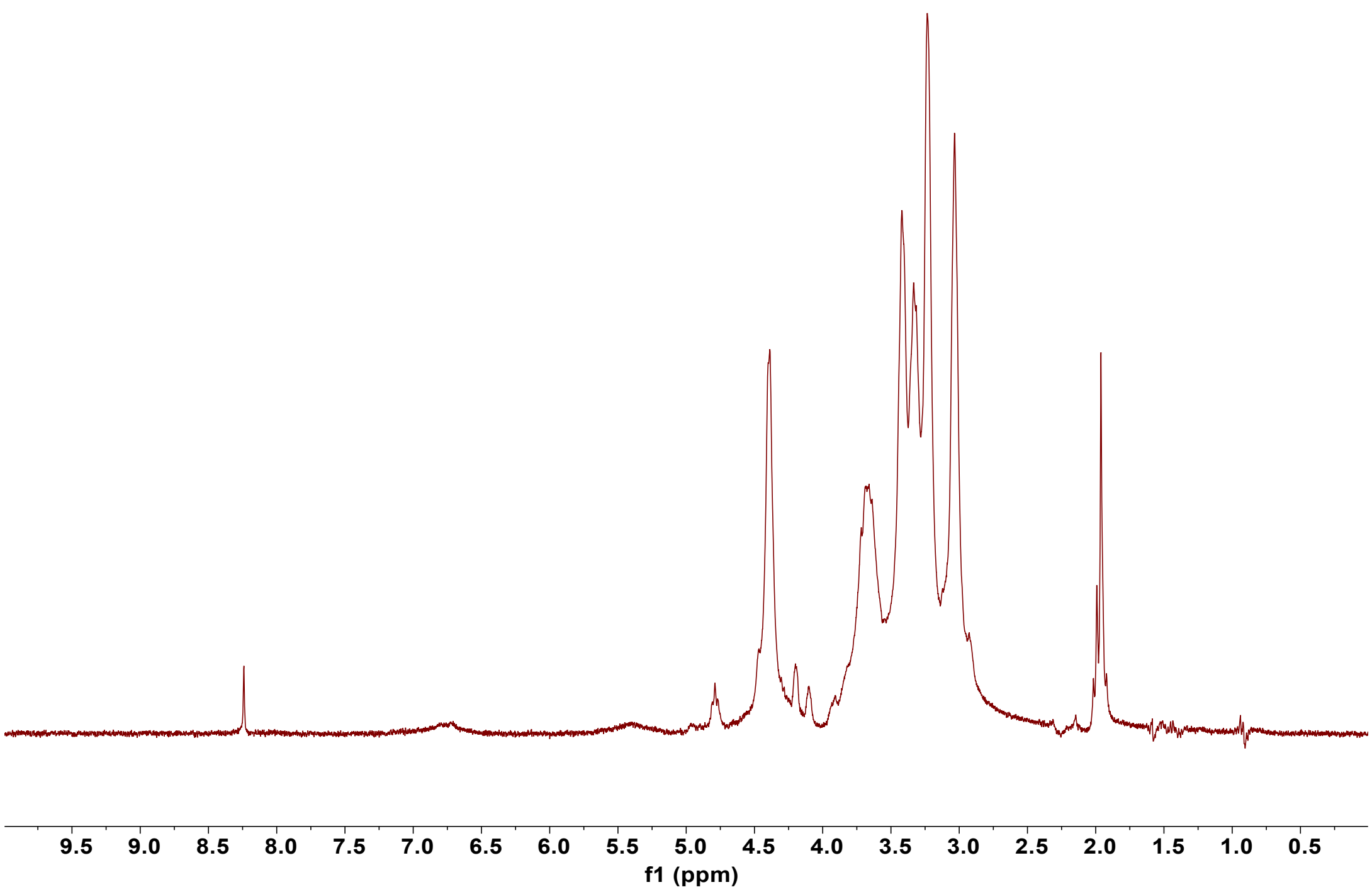

Figure S34. Diffusion edited ^{1}H NMR spectrum ([P_{4444}][OAc]:DMSO-d_6, 1:4 wt%; 400 MHz; 65°C) of CS-6 before bleaching (2.5 wt%). Some fractions remained insoluble.

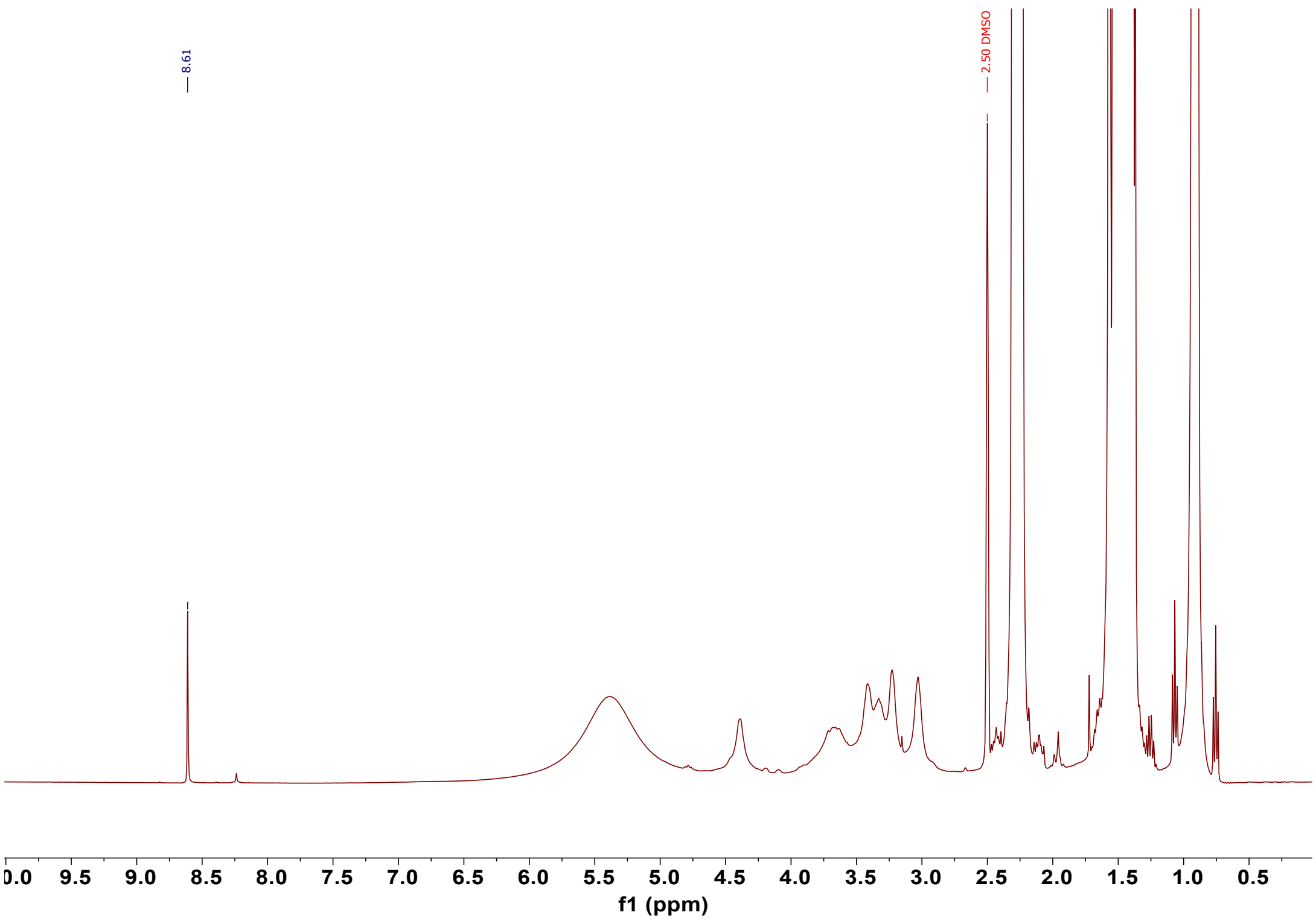

Figure S35. Quantitative ^{1}H NMR spectrum ([P_{4444}][OAc]:DMSO-d_6, 1:4 wt%; 400 MHz; 65°C) of CS-6 after bleaching (2.5 wt%).

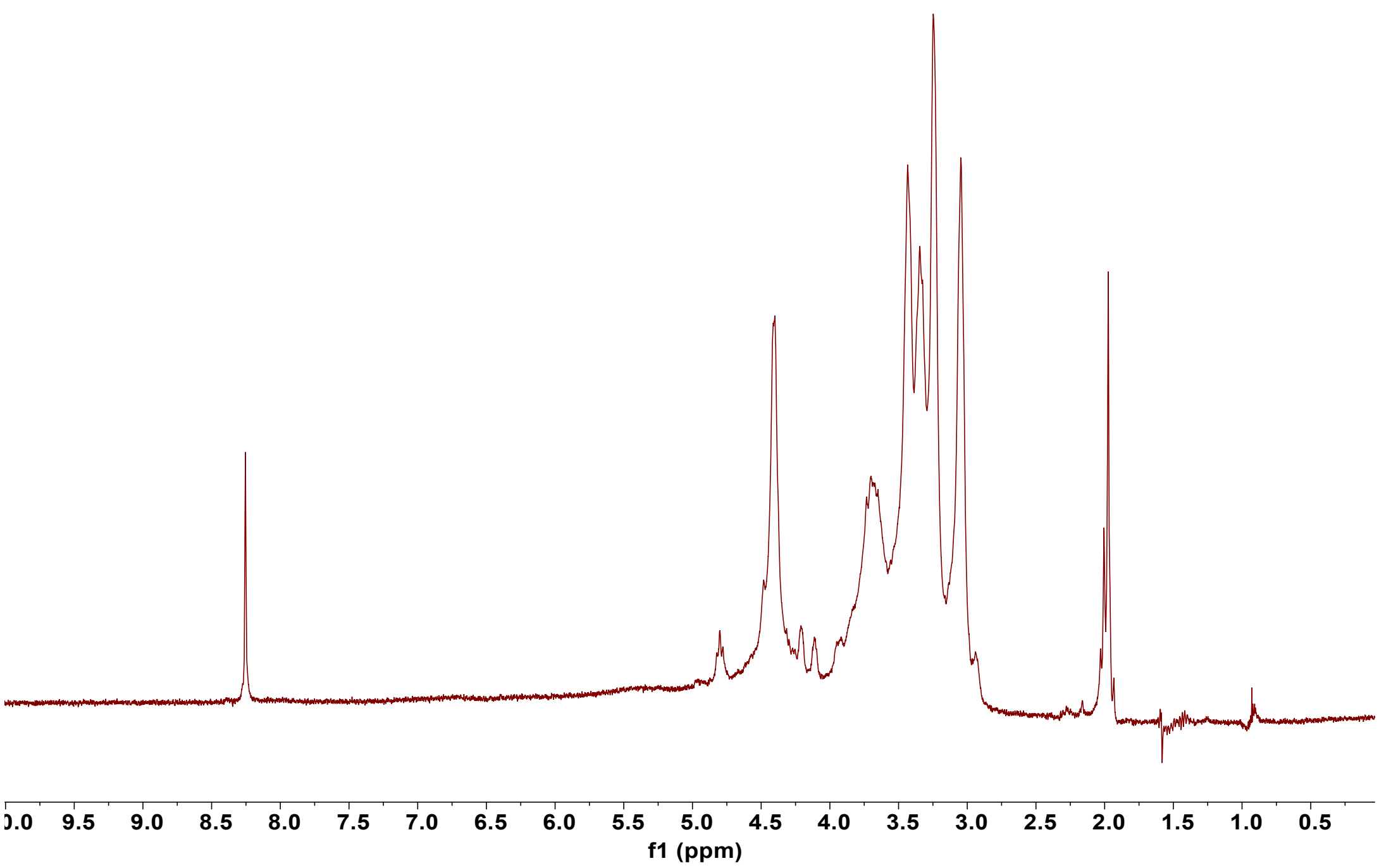

Figure S36. Diffusion edited ^{1}H NMR spectrum ([P_{4444}][OAc]:DMSO-d_6, 1:4 wt%; 400 MHz; 65°C) of CS-6 after bleaching (2.5 wt%).

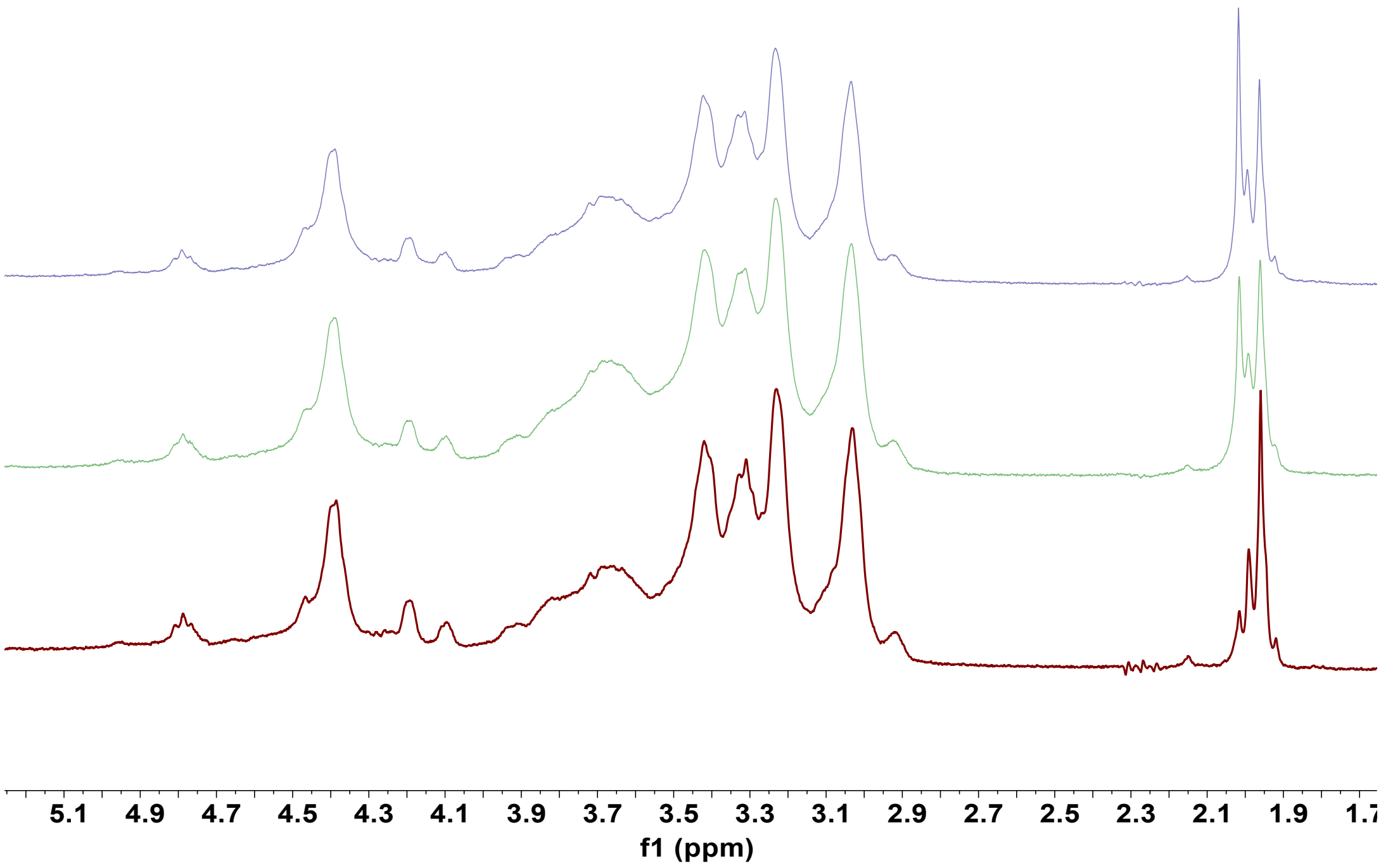

Figure S37. Comparison of the diffusion edited ^{1}H spectra of the bleached residual solids obtained from the treatment with AcOH. **Bottom**: Blank (no treatment); **Middle**: AcOH only; **Top**: AcOH with catalyst.

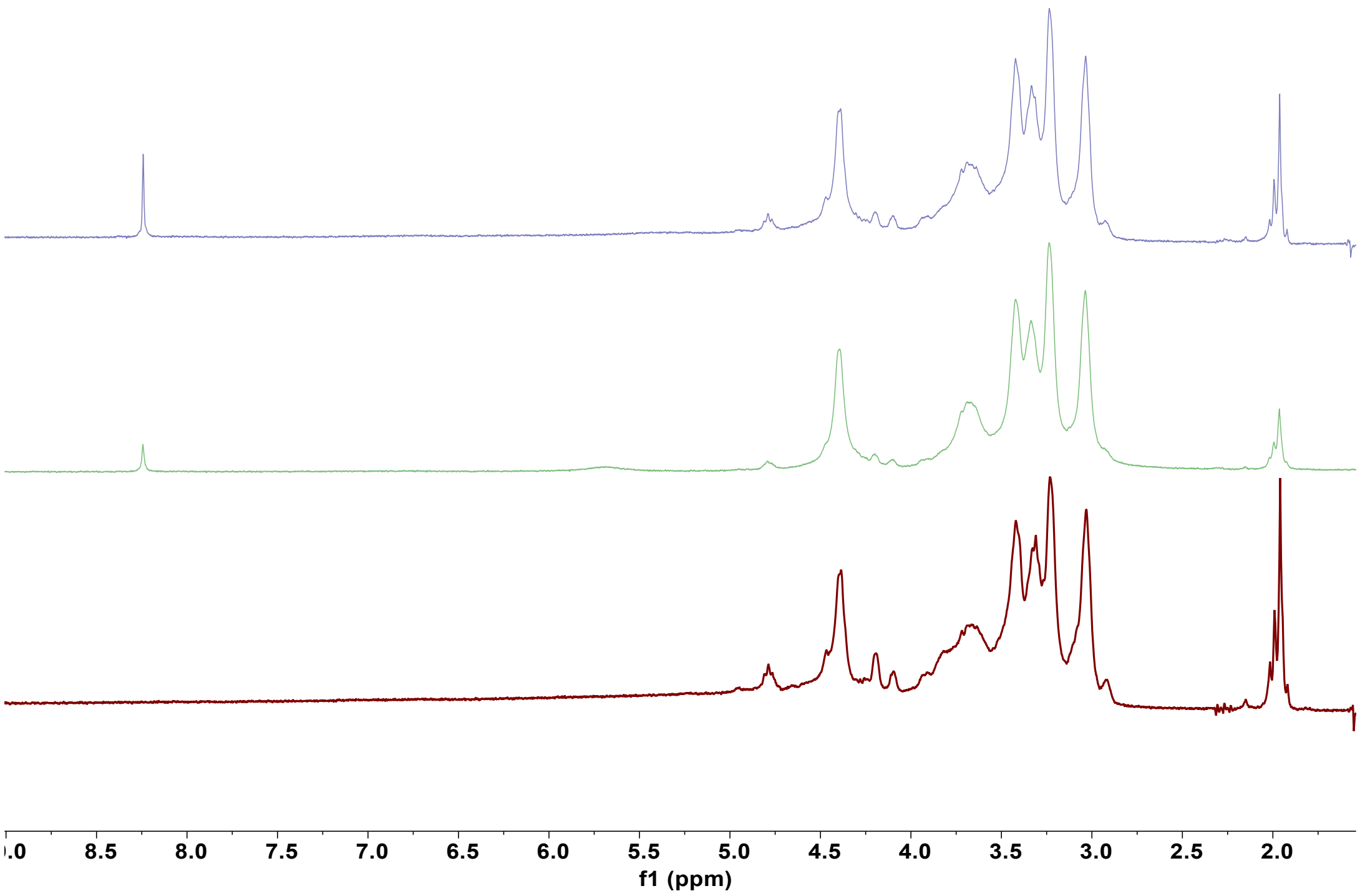

Figure S38. Comparison of the diffusion edited ^{1}H spectra of the bleached residual solids obtained from the treatment with HCOOH. **Bottom**: Blank (no treatment); **Middle**: HCOOH only; **Top**: HCOOH with catalyst.

1.6. Carbohydrate content and composition by GC–FID after acidic methanolysis

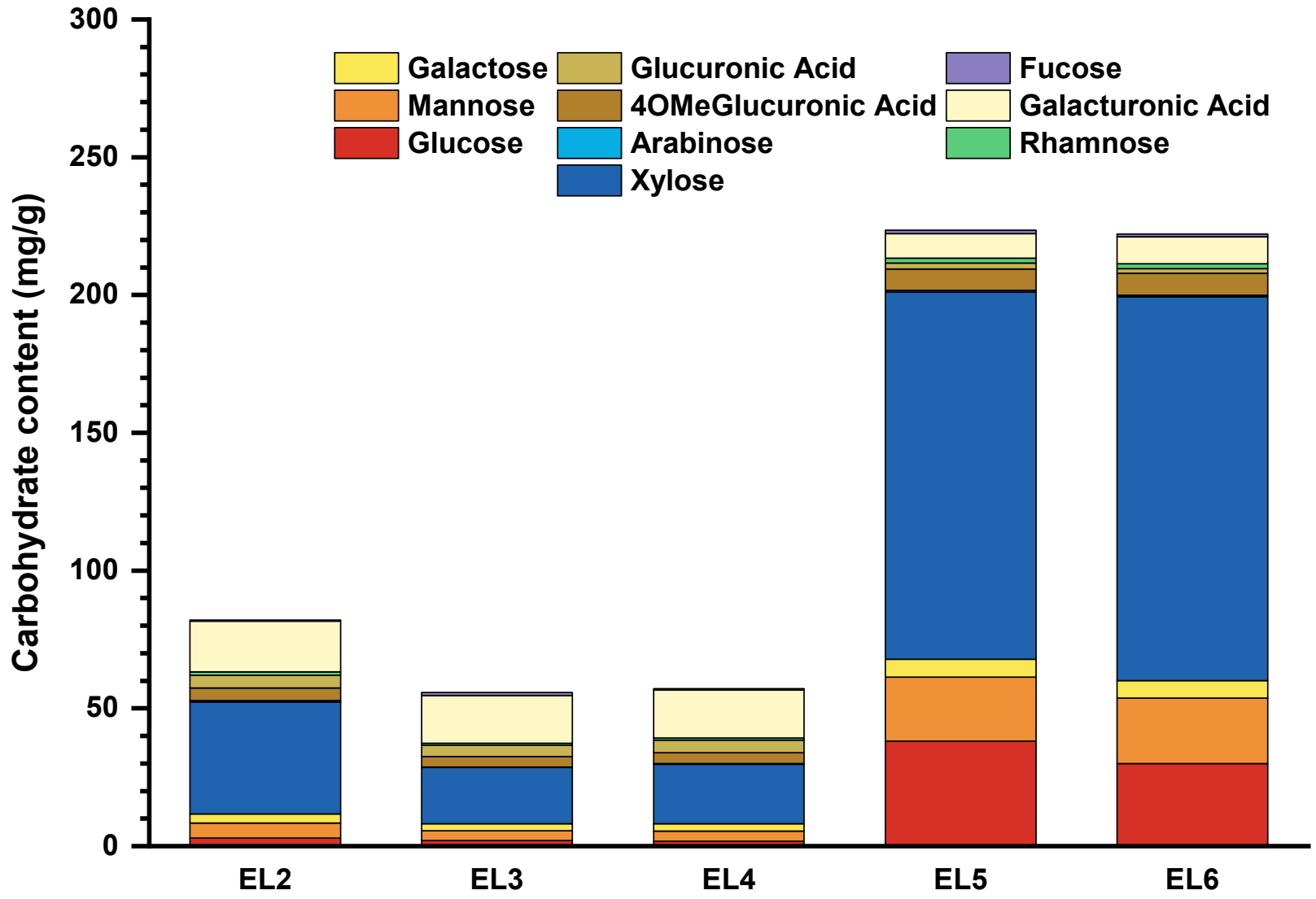

Figure S39. Monosaccharide contents after acidic methanolysis for the lignin samples EL2-6.

Table S3. Data for the monosaccharide contents after acidic methanolysis

Sample	Total sugar content	Glucose	Mannose	Galactose	Xylose	Arabinose	Rhamnose	4OMe-Glucuronic acid	Glucuronic acid	Galacturonic acid	Fucose
	[mg/g]	[mg/g]	[mg/g]	[mg/g]	[mg/g]	[mg/g]	[mg/g]	[mg/g]	[mg/g]	[mg/g]	[mg/g]
EL2	82.02 (± 2.38)	2.98 (± 0.13)	5.4 (± 0.17)	3.3 (± 0.11)	40.69 (± 1.67)	0.49 (± 0.03)	1.2 (± 0.08)	4.58 (± 0.17)	4.58 (± 0.17)	18.4 (± 0.76)	0.4 (± 0.02)
EL3	55.75 (± 3.06)	2.03 (± 0.12)	3.55 (± 0.65)	2.5 (± 0.18)	20.43 (± 1.45)	0.16 (± 0.15)	0.79 (± 0.06)	3.84 (± 0.16)	4.01 (± 0.5)	17.33 (± 1.36)	1.1 (± 1.14)
EL4	57.11 (± 6.26)	1.82 (± 0.02)	3.6 (± 1.4)	2.67 (± 0.4)	21.62 (± 0.9)	0.29 (± 0.08)	0.82 (± 0.01)	3.89 (± 0.19)	4.56 (± 0.8)	17.43 (± 2.92)	0.4 (± 0.08)
EL5	223.48 (± 5.37)	38.12 (± 1.17)	23.23 (± 0.52)	6.48 (± 0.17)	133.22 (± 3.08)	0.6 (± 0.03)	1.79 (± 0.04)	7.85 (± 0.28)	2.07 (± 0.59)	8.96 (± 0.5)	1.17 (± 0.08)
EL6	222.11 (± 5.65)	29.91 (± 0.56)	23.77 (± 0.31)	6.41 (± 0.13)	139.26 (± 4.37)	0.53 (± 0.05)	1.79 (± 0.05)	7.98 (± 0.27)	1.71 (± 0.52)	9.76 (± 1.63)	1.0 (± 0.52)

Note: STDEV in brackets; n = 3

1.7. Corrected lignin mass balances and yields

Table S4. The yields of extracted lignin (EL), residual solids (RS), and residual lignin (RL) obtained via the Amb-15 catalyzed REx

Entry	Label	Conditions		Yield (wt% of original lignin)[b]	
		Solvent	Catalyst	Non corrected	Corrected for carbohydrate content
2	EL2	EtOH	Yes	45.6	41.8
3	EL3	AcOH	No	58.6	55.3
4	EL4		Yes	69.6	65.6
5	EL5	HCOOH	No	136.0	105.5
6	EL6		Yes	140.2	109.1

[a]wt% yield of lignin with respect to the initial lignin content in the biomass (S-500).

1.8. Functionalization reactions during REx

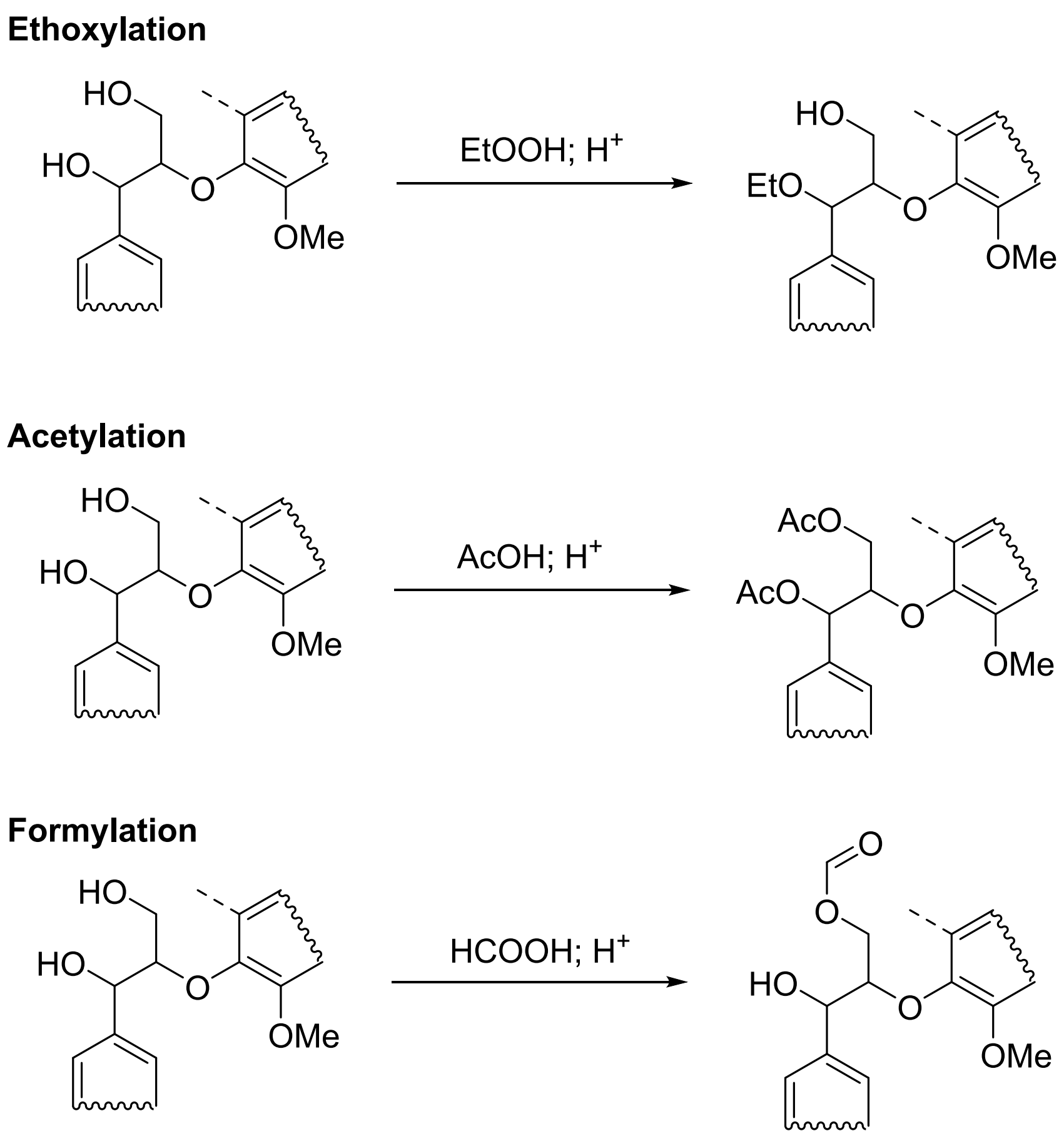

Figure S41. Occurring functionalization reactions during reactive extraction (REx).
